# Interim analysis from a phase 2 randomized trial of EuCorVac-19: a recombinant protein SARS-CoV-2 RBD nanoliposome vaccine

**DOI:** 10.1186/s12916-022-02661-1

**Published:** 2022-11-30

**Authors:** Jonathan F. Lovell, Yeong Ok Baik, Seuk Keun Choi, Chankyu Lee, Jeong-Yoon Lee, Kazutoyo Miura, Wei-Chiao Huang, Young-Shin Park, Sun-Je Woo, Sang Hwan Seo, Jae-Ouk Kim, Manki Song, Chung-Jong Kim, Jae-Ki Choi, Jieun Kim, Eun Ju Choo, Jung-Hyun Choi

**Affiliations:** 1 Department of Biomedical Engineering, University at Buffalo, Buffalo, NY USA; 2 Eubiologics, R&D Center, EuBiologics Co., Ltd., Chuncheon, South Korea; 3 National Institute of Allergy and Infectious Diseases, National Institutes of Health, Rockville, MD USA; 4 POP Biotechnologies, Buffalo, NY USA; 5 International Vaccine Institute, Gwanak-Gu, Seoul, South Korea; 6 Department of Internal Medicine, Ewha Womans University Seoul Hospital, Seoul, South Korea; 7 Catholic University of Korea, Bucheon St. Mary’s Hospital, Bucheon, South Korea; 8 Hanyang University College of Medicine, Seoul, South Korea; 9 Soonchunhyang University Bucheon Hospital, Bucheon, South Korea; 10 Department of Infectious Diseases, College of Medicine, Eunpyeong St. Mary’s Hospital, The Catholic University of Korea, Seoul, South Korea

**Keywords:** COVID-19, SARS-CoV-2, RBD, Vaccine, Liposome, Adjuvant

## Abstract

**Background:**

Numerous vaccine strategies are being advanced to control SARS-CoV-2, the cause of the COVID-19 pandemic. EuCorVac-19 (ECV19) is a recombinant protein nanoparticle vaccine that displays the SARS-CoV-2 receptor-binding domain (RBD) on immunogenic nanoliposomes.

**Methods:**

Initial study of a phase 2 randomized, observer-blind, placebo-controlled trial to assess the immunogenicity, safety, and tolerance of ECV19 was carried out between July and October 2021. Two hundred twenty-nine participants were enrolled at 5 hospital sites in South Korea. Healthy adults aged 19–75 without prior known exposure to COVID-19 were vaccinated intramuscularly on day 0 and day 21. Of the participants who received two vaccine doses according to protocol, 100 received high-dose ECV19 (20 μg RBD), 96 received low-dose ECV19 (10 μg RBD), and 27 received placebo. Local and systemic adverse events were monitored. Serum was assessed on days 0, 21, and 42 for immunogenicity analysis by ELISA and neutralizing antibody response by focus reduction neutralization test (FRNT).

**Results:**

Low-grade injection site tenderness and pain were observed in most participants. Solicited systemic adverse events were less frequent, and mostly involved low-grade fatigue/malaise, myalgia, and headache. No clinical laboratory abnormalities were observed. Adverse events did not increase with the second injection and no serious adverse events were solicited by ECV19. On day 42, Spike IgG geometric mean ELISA titers were 0.8, 211, and 590 Spike binding antibody units (BAU/mL) for placebo, low-dose and high-dose ECV19, respectively (*p* < 0.001 between groups). Neutralizing antibodies levels of the low-dose and high-dose ECV19 groups had FRNT_50_ geometric mean values of 129 and 316, respectively. Boosting responses and dose responses were observed. Antibodies against the RBD correlated with antibodies against the Spike and with virus neutralization.

**Conclusions:**

ECV19 was generally well-tolerated and induced antibodies in a dose-dependent manner that neutralized SARS-CoV-2. The unique liposome display approach of ECV19, which lacks any immunogenic protein components besides the antigen itself, coupled with the lack of increased adverse events during boosting suggest the vaccine platform may be amenable to multiple boosting regimes in the future. Taken together, these findings motivate further investigation of ECV19 in larger scale clinical testing that is underway.

**Trial registration:**

The trial was registered at ClinicalTrials.gov as # NCT04783311.

**Supplementary Information:**

The online version contains supplementary material available at 10.1186/s12916-022-02661-1.

## Background

Severe acute respiratory syndrome coronavirus 2 (SARS-CoV-2) is the etiological agent of the coronavirus disease 2019 (COVID-19) pandemic that triggered an estimated 18 million deaths in 2020 and 2021 based on excess morality [1] and has re-emerged in waves of new variants [2]. In a remarkable display of the power of modern biotechnology, many safe, effective, and novel vaccines have effectively been rapidly developed and deployed [3]. As the human global population becomes exposed to COVID-19 viral infection and vaccines, the pandemic is expected to shift from pandemic to endemic, and many questions remain on how COVID-19 vaccines will be used in future years [4]. Vaccines are poised to remain a key part of mitigation strategies. How boosting regimes function in this context of emerging strains remains to be seen over coming years. Approved vaccines to date include those based on mRNA, viral-vectors, attenuated SARS-CoV-2, and recombinant proteins [4]. Most approved vaccines are based on the large Spike protein of the viral surface. On the other hand, numerous preclinical vaccine candidates have focused on the receptor-binding domain (RBD) portion of the Spike, which has the theoretical advantage of being the antigen mostly responsible for neutralizing antibody generation, although its smaller size may provide fewer T cell epitopes [5]. Several RBD vaccines have also moved into clinical testing including RBD dimers (V-01 [6] and FINLAY-FR [7]) as well as an adjuvanted, protein-scaffold displaying the RBD [8].

EuCorVac-19 (ECV-19) is an RBD-based vaccine that displays the RBD on immunogenic liposomes that also contain monophosphoryl lipid A (MPLA) derived from genetically engineered *Escherichia coli* (EcML). EcML is a vaccine adjuvant produced in *E. coli* featuring a modulated lipopolysaccharide biosynthetic pathway [9]. The RBD antigen is displayed on the surface of EcML-containing liposomes via interaction between the RBD polyhistidine-tag and liposome-embedded cobalt-porphyrin-phospholipid (CoPoP), an approach which has been shown to enhance antigen immunogenicity in preclinical studies [10]. Some of the additional unique features of ECV-19 include co-delivery (as opposed to co-administration) of adjuvant and antigen, and making use of a protein-free scaffold that would not distract the immune response in scenarios involving multiple boosting regimes. Here, the interim results of a phase 2 clinical study are reported.

## Methods

### Study design and participants

Initial study of a phase 2 randomized, observer-blind, placebo-controlled study in healthy adults between 19 and 75 years old, who did not have known prior COVID-19 infection or vaccination (based on interviews, and not any specific screening for pre-existing SARS-CoV-2 antigens or antibodies), was carried out between July and October 2021 at 5 hospital sites in South Korea. Written informed consent was obtained from all participants. The trials were done according to the principles of the Declaration of Helsinki and Good Clinical Practice. This study was approved in South Korea by the Korea Ministry of Food and Drug Safety (approval #33,475) and Institutional Review Board at 5 sites; Catholic University of Korea, Eunpyeong St. Mary’s Hospital (approval #PC21BDDF0015), Soonchunhyang University Bucheon Hospital (approval #SCHBC 2021–03-017), Hanyang University College of Medicine (approval #GURI 2021–03-044), Catholic University of Korea, Bucheon St. Mary’s Hospital (approval #HC21BDDS0027), and Ewha Womans University Seoul Hospital (approval #SEUMC 2021–03-038). The study was registered with ClinicalTrials.gov (NCT04783311). The primary objective was to confirm the SARS-CoV-2 specific immune response when administering ECV19 to healthy adults. The secondary objective was to confirm the safety and tolerance to SARS-CoV-2. A total of 270 adults were screened, and 229 participants were enrolled. Twenty-four exclusion criteria for the trial were used and are listed in Additional file 1: Table S1.

### Randomization and masking

This study used the Interactive Web Response System (IWRS) for randomization. Subjects were assigned to each treatment group in a ratio of 10:10:3 (low dose group to high dose group to placebo comparator group) and stratified by age (19 to 50 years of age versus 51 to 75 years of age). The randomization manager randomly chose a block size among the multiples of the number of treatment groups and generated randomization tables using SAS software (version 9.4 or above). Individuals who provided written informed consent to participation were given a screening number first. Afterwards, those who met the inclusion criteria were randomized by the central randomization plans. Allocation of unique codes by group was managed by IWRS at the central enrollment center. Pharmacists identified the subject’s randomization number via IWRS to release the appropriate investigational product.

### Investigational product

The GMP-grade investigational product, ECV19, was manufactured by Eubiologics. ECV19 comprises the SARS-CoV-2 RBD antigen with a poly-histidine tag produced by a stable Chinese Hamster Ovary cell line. The sequence for the his-tagged RBD (Wuhan-Hu-1 strain, GenBank: MT380724.1) was cloned into the pcDNA3.4 expression plasmid to generate the stable cell line used to generate the purified RBD (Additional file 1: Figure S1). The RBD is displayed on nanoliposomes that include EcML and CoPoP, along with the inactive carrier lipids dioleoylphosphatidylcholine and cholesterol. The high-dose group received 20 μg RBD antigen, 20 μg EcML, and 40 μg CoPoP. The low-dose group received 10 μg RBD along with 10 μg EcML and 20 μg CoPoP. The placebo group received a saline injection (Daihan Isotonic Sodium Chloride).

### Clinical procedures

The overall schedule for safety and immunogenicity assessment is shown in Additional file 1; Table S2. This study provides an interim analysis of safety and immunogenicity through 8 weekly visits during the study. Intramuscular vaccination with the investigational product occurred on Visit 2 (also referred to as day 0 herein) and Visit 5 (referred to as day 21), and serum was collected on visit 8 (referred to as day 42). No testing for COVID-19 was carried out during this period. Visit 2, 5, and 8 were conducted between July 27, 2021, and October 15, 2021. While the trial was designed as an observer-blind study, participants were not informed of which vaccine or placebo they received until 4 weeks after visit 8. All medical events occurring before the 1st dose of the investigational product were documented as medical history. Assessment of adverse events and severity of solicited or unsolicited adverse events in subjects enrolled in this study were classified based on “MFDS, Guidelines for the assessment of severity of adverse events in vaccine trials” [11]. Severities of unsolicited AEs including immediate adverse events (anaphylaxis related) observed within 30 min of dosing were classified based on the same guidance. Adverse events of special interest were considered according to the guidance document “MFDS, Considerations in COVID-19 vaccines development” [12]. The clinical protocol is included in Additional file 2.

### Assessment of antibodies at the International Vaccine Institute (IVI)

The titer of the antibody binding to the spike protein (Acro Biosystems, cat# SPN-C52H9) (anti-S titer) was assessed by ELISA at IVI (Seoul, Korea) using qualified methods as previously described [13]. The antibody titer was expressed as binding antibody unit per mL (BAU/mL) based on a standard curve generated from dilutions of COVID-19 convalescent serum provided by Korea National Institutes of Health, which was calibrated to the unit of WHO international standard (NIBSC code 20/136). If at least two dilutions of each sample were not included within the standard curve due to low OD value, then the sample was considered as 0.5 BAU/mL. To measure the SARS-CoV-2-specific neutralizing antibody activity induced by the vaccine, a FRNT was performed using Wuhan strain of SARS-CoV-2 using qualified methods as described previously [14].

### Assessment of virus neutralization by microneutralization assay (MNA)

A subset of samples was sent to Vismederi (Siena, Italy) for assessment of MNA of the Wuhan, Delta and Omicron strains. MNA was carried out as reported [15].

### Assessment of RBD antibodies

SARS-CoV-2 RBD-specific antibody responses were determined by ELISA at Eubiologics. Briefly, ELISA plates (NUNC Maxisorp, Thermo Scientific) were coated with 100 ng/well of purified SARS-CoV-2 RBD protein in 100 μL of PBS overnight at 4 °C. Each antigen-coated well was blocked with PBS containing 2% non-fat milk for 1 h at 37 °C and washed with PBS containing 0.05% Tween-20 (PBST). Then, each serum was serially diluted and incubated for 1.5 h at 37 °C. The plates were washed, HRP Anti-human IgG (BD Pharmingen) was added, and the plates were incubated for 1 h at 37 °C. After incubation, the plates were washed six times with PBST and tetramethylbenzidine peroxidase substrate (TMB) was added to develop the color. The reaction was stopped by 0.5 M H_3_PO_4_, and the optical density (OD) was measured at a wavelength of 450 nm using ELISA plate reader. The RBD-ELISA titers were converted to binding antibody unit (BAU) using WHO international standard (NIBSC code: 20/136) and compared with the in-house standard serum (COVID-19 positive human convalescent serum panel obtained from Access biologicals).

### Assessment of cellular response for a subset of samples from Eunpyeong St. Mary’s Hospital

Cellular response was assessed by analyzing the vaccine antigen-specific T cell count in peripheral blood mononuclear cells (PBMCs) (without proliferation by additional culture) in the blood of the participants treated with SARS-CoV-2 spike RBD vaccine antigens (low dose group, *n* = 17; high dose group, *n* = 21; placebo group, *n* = 7). After separation of PBMCs from blood samples of study subjects (treated with heparin), the PBMCs were slowly frozen at − 70 °C, and after 3 days, they were stored at <  − 190 °C in liquid nitrogen. After thawing, 2.5 × 10^5^ cells were split into each well of a 96-well plate which was coated with anti-human IFNγ or anti IL-4 monoclonal antibody, and then, a SARS-CoV-2 S1 peptide pool (total 166 peptide) in RPMI (with 100 units/mL penicillin, 1 mg/mL streptomycin, 10% heat inactivated fetal calf serum) was added for 18–20 h. Then, detection monoclonal antibody and streptavidin alkaline phosphatase were cultured and treated with 5-bromo-4-chloro-3-indolyl phosphate/nitro blue tetrazolium (BCIP/NBT) substrate, and expression was measured. Spot forming cells (SFC)/10^5^ cells were counted in each sample by an ELISpot counter.

### Assessment of anti-S, anti-RBD, IgG subclass (anti-RBD) and anti-his-tag responses for a subset of samples from Eunpyeong St. Mary’s Hospital

A subset of samples was sent to the National Institutes of Health (Rockville, Maryland, USA) for exploratory analysis. The basic methodology for ELISA was carried out as described [16]. For anti-S and anti-RBD antibody measurement, the same spike and RBD proteins used at IVI and Eubiologics were utilized to coat ELISA plates, and ELISA units were determined for each test serum collected from this phase 2 study and post-immune Pfizer or Johnson & Johnson vaccination serum purchased from Access Biologicals (Vistas, USA). The WHO International Standards (National Institute for Biological Standards and Control, catalog #20/268, Hertfordshire, England) were also evaluated in the same assays, and the ELISA units of test sera were converted to BAU/mL concentrations.

For IgG subclass ELISA against RBD protein, all test samples were diluted to 2 anti-RBD BAU/mL total antibody level.

For anti-his-tag ELISA, serum samples were tested at 1:200 dilution using ELISA plates coated with 200 ng/well of 10 × His peptide (Abcam, Catalog # ab14943). As a standard, anti-His-Tag chimeric human monoclonal antibody (Sigma, Catalog # SAB5600096) was included at fourfold serial dilutions from 250 ng/mL.

### Statistical analyses

Chi-square tests were utilized to compare demographic characteristics. For adverse event (AE), the difference in total number of AEs (including all grade 1, 2, and 3 AEs) among the placebo, low-dose, and high-dose group was compared by a chi-square test first. If significant, the difference between placebo and low-dose group and between placebo and high-dose group was evaluated by Fisher’s exact tests, and Bonferroni corrected *p*-values are shown. To compare ELISA and FRNT results among different groups at the same time, a Mann–Whitney test (to compare two groups) or a Kruskal–Wallis test followed by Dunn’s multiple comparison tests (to compare more than two groups) were used. The comparison among days 0, 21, and 42 within the group was done by a Friedman test followed by Dunn’s multiple comparison tests. Correlations between anti-RBD and anti-S titers and between anti-RBD titer and FRNT were determined by a Pearson test using log-transformed data. Statistical analysis was carried out using the GraphPad Prism software version 9.3.

### Role of funding source

This research was supported by a grant of the Korea Health Technology R&D Project through the Korea Health Industry Development Institute, funded by the Ministry of Health & Welfare, Republic of Korea (grant number: HQ20C0076), and by Eubiologics. The Ministry of Health & Welfare had no role in the study design, data collection, data analysis, data interpretation, or writing of the report. Staff at Eubiologics were involved in preparing and releasing the investigational product, coordinating contract research organizations, and assessing RBD antibody titers (Fig. 5).

## Results

A randomized phase 2 trial was designed and carried out after enrolling 229 healthy adults. Participants received the initial vaccine or placebo injection between July 7 and August 24, 2021. The demographics of the participants is shown in Table 1, and in general, they were similar across the groups, except for the male to female ratio between high- and low-dose ECV-19 groups, which was barely significant (*p* = 0.0474 by Fisher’s exact test with Bonferroni correction). The mean age of participants was between approximately 40–42 years in age, with a standard deviation of 11–13 years. All participants were ethnic Korean (Asian). The body mass average of the various groups ranged from 23 to 24.

**Table 1:** Participant baseline demographic characteristics

	**High-dose ECV19**	**Low-dose ECV19**	**Placebo**
**Characteristics: *N***	100	100	29
**Sex**			
Male: no. (%)	44 (44.0)	62 (62.0)	18 (62.1)
Female: no. (%)	56 (56.0)	38 (38.0)	11 (37.9)
**Age** years (y)			
All (19–67 y): mean (SD)	40.8 (12.5)	42.0 (13.1)	39.6 (10.9)
19–34 y: no. (%)	29 (29.0)	36 (36.0)	11 (37.9)
35–64 y: no. (%)	69 (69.0)	62 (62.0)	18 (62.1)
65–67 y: no. (%)	2 (2.0)	2 (2.0)	0 (0.0)
**BMI**: mean (SD)	23.8 (2.8)	23.8 (3.0)	22.8 (2.6)

The race or ethnic group of all participants was Korean (Asian)

**Fig. 1:**
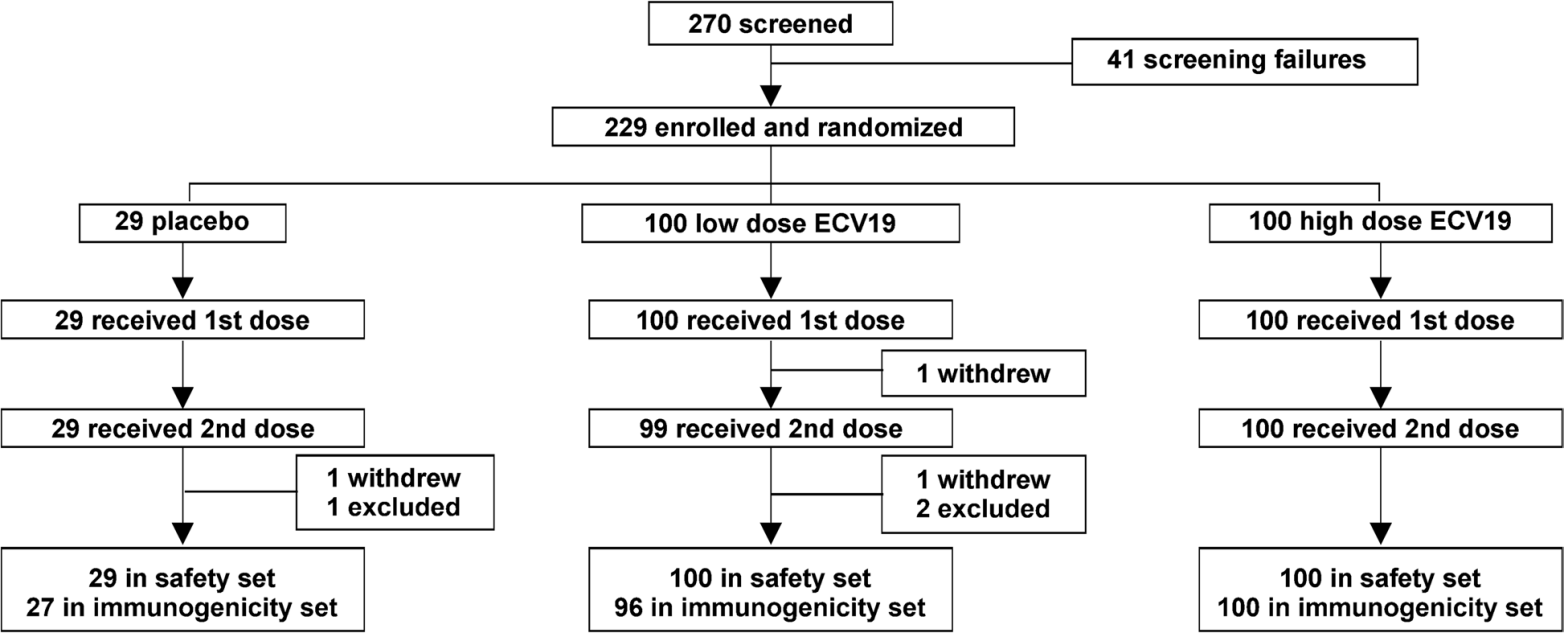
ECV19 clinical trial design

As shown in Fig. 1, 100 volunteers were allocated to the low-dose group of ECV19 (10 μg of RBD, 10 μg EcML, and 20 μg CoPoP), another 100 were allocated to the high-dose group (20 μg of RBD, 20 μg EcML and 40 μg CoPoP), and 29 were allocated to receive a saline placebo injection. Participants received intramuscular vaccine injections on day 0 and day 21. All 229 participants were included in the safety analysis. Two hundred twenty-three participants were included in the immunogenicity analysis. Three participants were excluded from subsequent immunogenicity analysis since their final serum collection occurred on days outside the permissible limit of one week following the day 42 collection time point. Two participants withdrew consent after the first injection. There was one fatality in the low-dose ECV19 group in a participant 19 days after the 2nd dose. The participant was found to have underlying hyperlipidemia, and based on cardiovascular angiography, arteriosclerosis was determined to be the underlying cause, and not vaccination-induced ischemia. The clinical investigator determined this severe adverse event was unrelated to the investigational product and the independent Data and Safety Monitoring Board concurred with the investigator opinion after reviewing all safety data, determining it did not match criteria for study discontinuation.

**Fig. 2:**
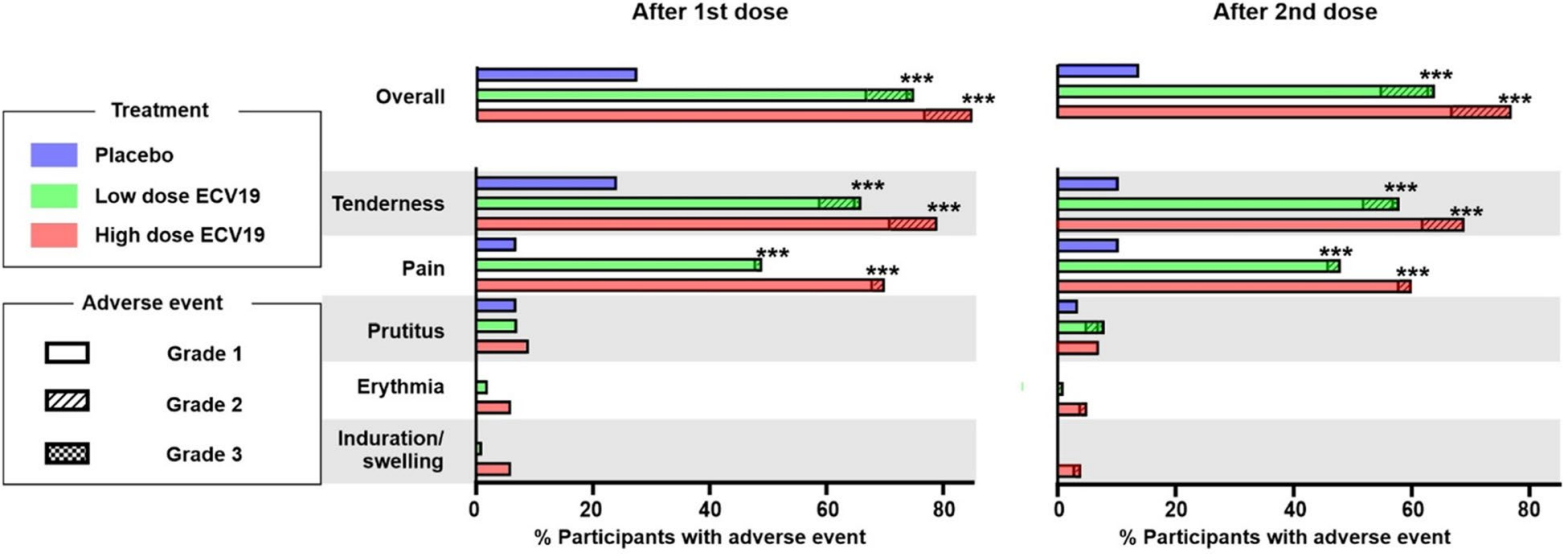
Summary of local adverse events in the trial participants. Participants in indicated group experiencing indicated adverse events are shown. Asterisks show statistical difference between placebo arm based on Fisher’s exact test with Bonferroni correction (***p < 0.001)

Solicited adverse events were recorded among all participants. No immediate adverse events (within 30 min of immunization) occurred during the study. As shown in Fig. 2, local adverse events were frequently observed. Tenderness and pain at the injection site was observed in the majority of participants and at a higher rate in participants receiving ECV19 compared to placebo. These were classified mostly as grade 1, along with a smaller number of grade 2 adverse events. The incidence of other local reactions including pruritus, erythema, and induration/swelling was less common. In all groups, most local adverse events resolved within 2 days, and all grade 2 adverse events fully resolved within 6 days. In the high-dose ECV19 group, following boosting, all grade 2 adverse events fully resolved within 2 days. Two grade 3 local reactions were recorded in two different participants, both in the low-dose ECV19 group, after the 2nd injection. One was grade 3 pruritus which fully resolved after 1 day, and the other was tenderness, which improved to grade 2 after 1 day and fully resolved after 2 days. All solicited grade 3 adverse events resolved without intervention. No solicited laboratory (blood count and chemistry) adverse events were observed. There were no increased solicited local adverse events following boosting, relative to the initial immunization, and to the contrary, adverse events trended towards diminished frequency.

**Fig. 3:**
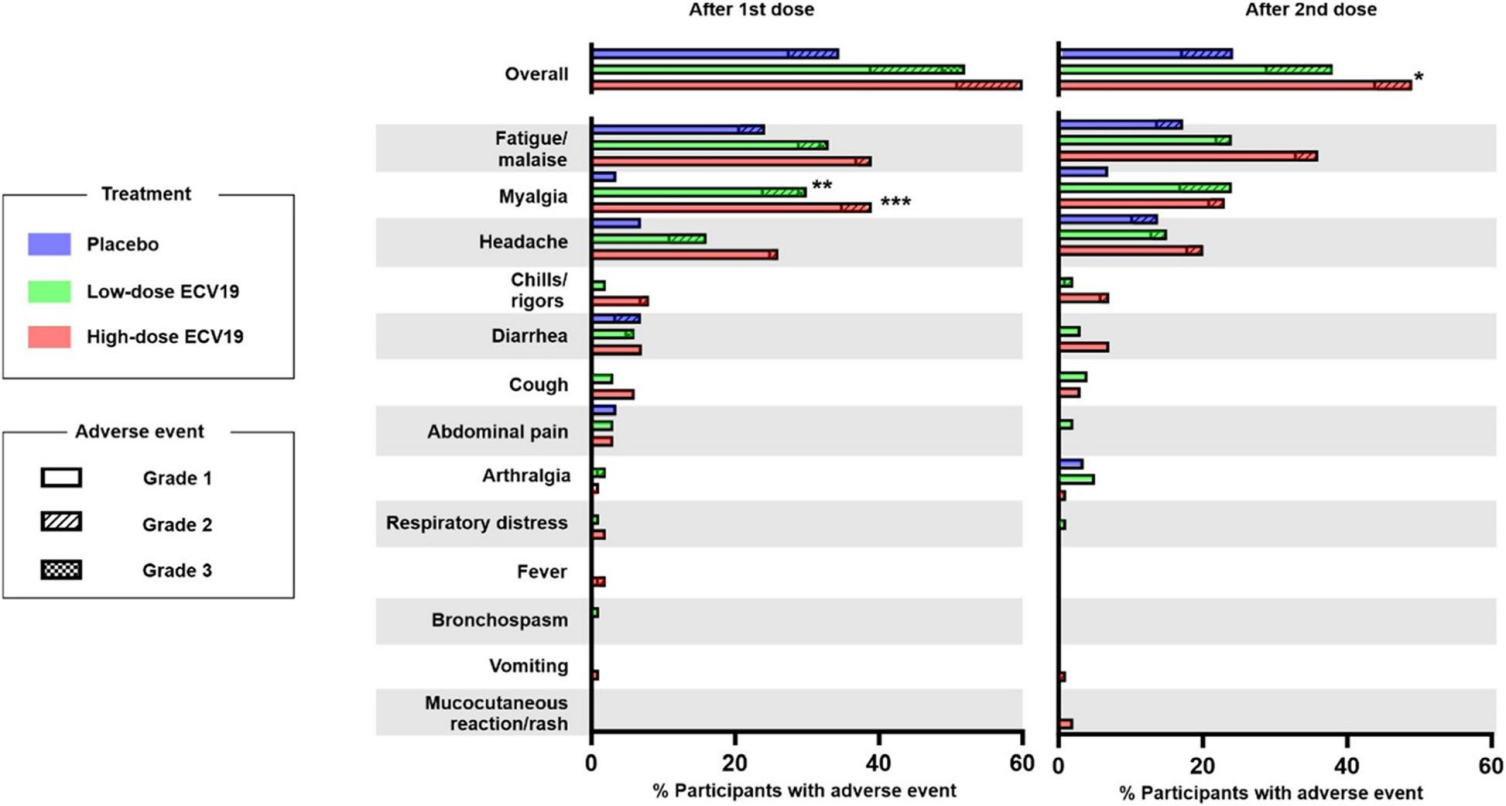
Summary of systemic adverse events in the trial participants. Participants in indicated group experiencing indicated adverse events are shown. Asterisks show statistical difference between placebo arm based on Fisher’s exact test with Bonferroni correction (*p < 0.05; **p < 0.01; ***p < 0.001)

Solicited systemic adverse events were also examined. As shown in Fig. 3, systemic adverse events were not as common as local ones, occurring in less than 60% of participants overall. The most common solicited adverse events in participants receiving ECV19 as well as the placebo were low grade fatigue/malaise, myalgia, and headaches. Other adverse events including chills/rigors, diarrhea, cough, abdominal pain, and arthralgia occurred with diminished frequency. Other systemic adverse events occurred only in a one or two participants included respiratory distress, fever, bronchospasm, vomiting, and mucocutaneous reaction/rash. Most solicited systemic adverse events were grade 1 and to a lesser degree grade 2, and most resolved within 2 days. All grade 2 adverse events fully resolved within 6 days. There were three grade 3 solicited adverse events all of which occurred after the initial immunization and all in the low-dose ECV19 group. One was grade 3 diarrhea in the low-dose ECV-19 group which occurred on the day after immunization and resolved by the next day. The next was grade 3 fatigue/malaise that onset a day after immunization and decreased to grade 2 by the next day and to grade 1 2 days after that. No other adverse reactions to the vaccine occurred in that participant. The third was grade 3 myalgia that occurred on the same day of immunization and was downgraded to grade 1 by the next day. Similar to the local adverse events, systemic ones also did not increase following boosting, relative to the initial immunization, and appeared to trend lower. No solicited grade 3 adverse events occurred following the 2nd dose.

**Fig. 4:**
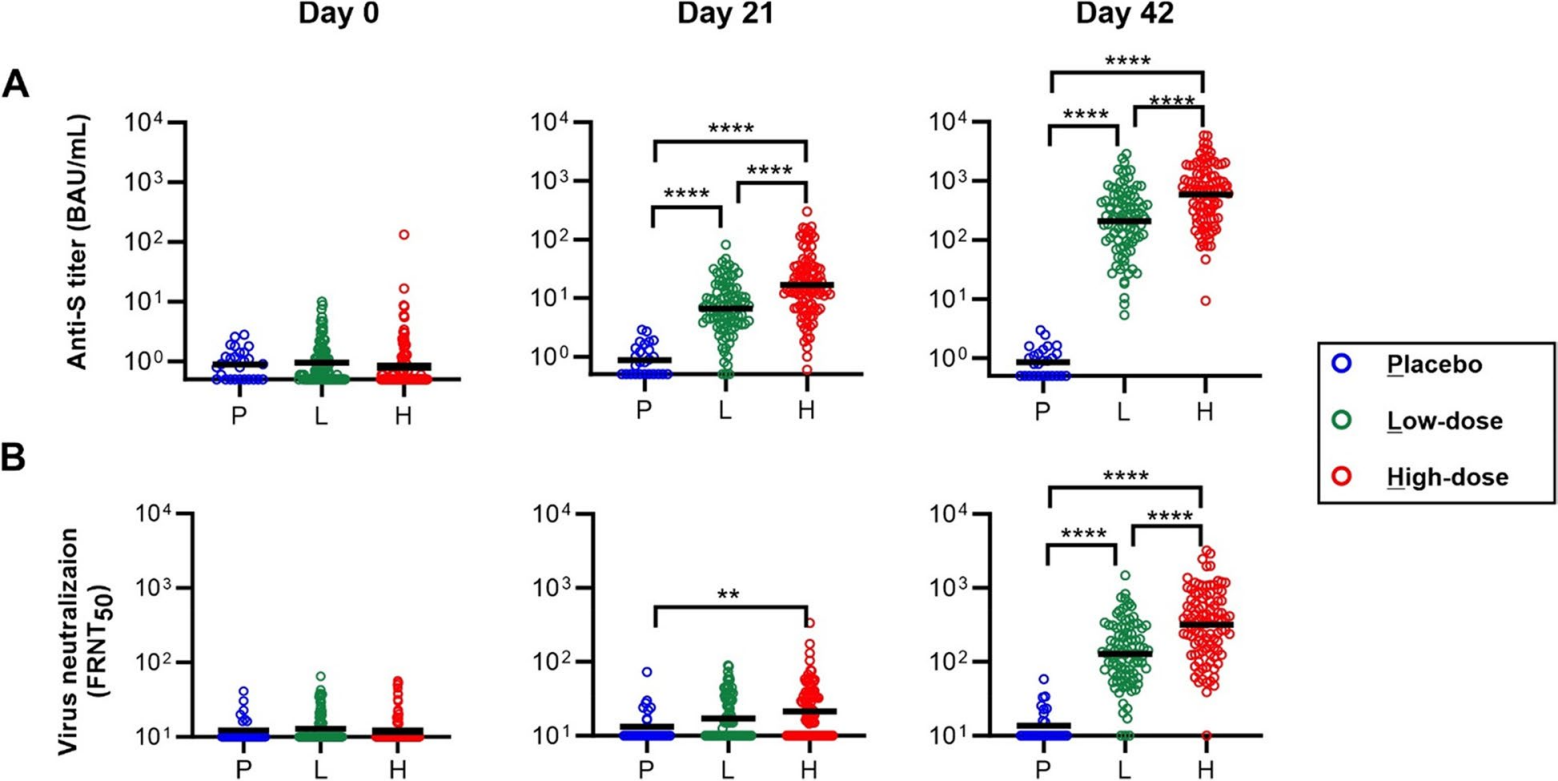
Induction of neutralizing antibodies by ECV19. **A** Anti-Spike (S) IgG titers and **B** FRNT neutralization of SARS-CoV-2 of serum sampled from participants in the indicated groups at the indicated time point. *N* = 27 for placebo, 96 for low-dose ECV19 and 100 for high-dose ECV19. Bars show geometric mean values within groups. Indicated **** and ** correspond to *p* values of < 0.0001 and < 0.01, respectively

The immunogenicity of ECV19 was assessed by serum antibody analysis, as shown in Fig. 4A. Although ECV19 utilizes only the RBD antigen, a qualified ELISA method was used to detect IgG antibodies against an independently-produced trimeric Spike ectodomain protein. Twenty-one days following initial immunization with low- or high-dose ECV19, elevated Spike antibodies were observed with geometric mean Spike antibody levels (anti-S IgG titer) rising from less than 1 BAU/mL to 7 and 17 BAU/mL, respectively. Following boosting, geometric mean anti-S IgG titers further increased to 211 and 590 BAU/mL in low- and high-dose ECV19, respectively, while the placebo group remained below 1 BAU/mL. Statistical analysis showed after following the 2nd injection, low-dose ECV19 had greater antibody levels relative to placebo and that high-dose ECV19 induced more antibodies compared to low-dose ECV19. There was also a significant increase in antibody levels between the 1st and 2nd ECV19 vaccine injection (Additional file 1: Figure S2). There were no significant differences in anti-Spike antibody levels between the 5 different trial sites (Additional file 1: Figure S3).

Induced antibodies were able to neutralize the Wuhan strain of SARS-CoV-2, based on FRNT as shown in Fig. 4B. At day 21, there was a significant increase in neutralizing activity only in the high-dose ECV-19 group, where geometric mean FRNT_50_ value reached 21, from the baseline value of 12. Following boosting, participants in both low- and high-dose ECV19 groups had significantly increased FRNT values, rising to geometric mean levels of 129 and 316, respectively. There was a significant difference between the placebo and low- and high-dose ECV19 groups for the neutralizing antibody titer on day 42.

When the impact of age (19–34 year vs. older groups) and sex on immunogenicity was evaluated, while there was no significant difference by age, female showed significant higher anti-S IgG titer (both on days 21 and 42) and FRNT_50_ values (day 42) than male (Additional file 1: Figure S4).

**Fig. 5:**
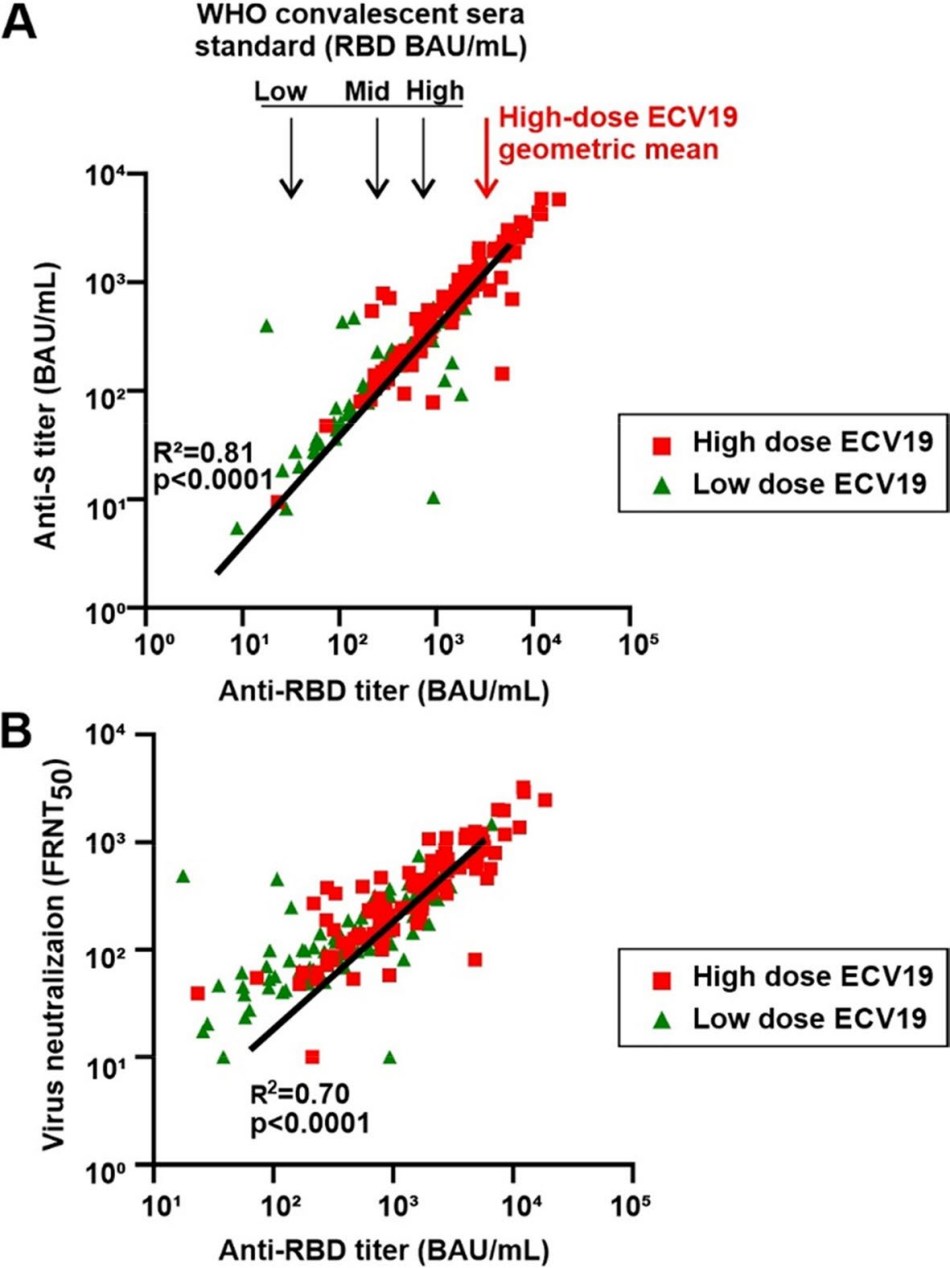
Antibody correlation analysis. **A** Anti-S IgG titer correlation with anti-RBD antibody levels. **B** Anti-RBD IgG titer correlation with neutralizing antibody levels. The best-fit line, R^2^ and *p* value for each panel were determined by a Pearson test using log-transformed data

Next, anti-RBD IgG titers were measured for day 42 sera. The titers for both high- and low-dose ECV19 correlated well with the corresponding anti-S IgG titers (Fig. 5A). Likewise, there was general correlation between anti-RBD IgG titers and neutralizing antibody titers (Fig. 5B).

Additional exploratory analysis was carried out with the antibodies induced by ECV19. For the analysis, serum samples (*n* = 45) from the Eunpyeong St. Mary’s Hospital site were utilized. Given that only the RBD is used as the immunogen for EVC19, the RBD to Spike antibody ratio would be expected to be higher compared to other vaccines using Spike as an immunogen. Using commercially available post-immune sera from the Pfizer mRNA vaccine and the Johnson & Johnson (JnJ) vaccine, it was apparent that ECV19 induced a substantially higher proportion of anti-RBD antibodies when plotted as a function of the anti-S antibody level (Additional file 1: Figure S5). This is not surprising considering the only antigenic component of ECV19 is the RBD, whereas the JnJ and Pfizer vaccine’s antigen is the entire Spike (of which the RBD only comprises ~ 15% of the total Spike length).

Exploratory T cell analysis was also carried out with a subset of participant samples (Additional file 1: Figure S6). Although this analysis was limited to *n* = 7 in the placebo group, *n* = 17 in the low-dose ECV19 group, and *n* = 21 in the high-dose ECV19 group, there was a significant difference between placebo group (median = 0 SFU per 10^5^ cells) and high-dose group (median = 44) for IFN-γ responses (*p* = 0.021) on day 42. While IL-4 response in high-dose group (median = 24) was also higher than that for placebo group (median = 8) on day 42, the difference did not reach significance. There was no significant difference between placebo and low-dose groups for either IFN-γ or IL-4 responses.

The RBD of ECV19 contains a six-residue histidine tag. We developed an assay to assess anti-his-tag response based on a chimeric anti-his tag antibody with mouse variable region and human constant region. When pre-immune sera from the high-dose group (*n* = 21) were tested at 1:200 dilution, the OD values in ECV19 immunized subjects were lower than that at 4 ng/mL of the chimeric antibody, which was barely above background signal of assay (Additional file 1: Figure S7). Similarly, post-immune sera from the same 21 vaccinees showed lower OD values than that at 4 ng/mL of the chimeric antibody.

Subclass analysis of ECV19 sera of the high-dose ECV19 group showed that IgG1 antibodies dominated the response and indeed were the only detectable antibodies (Additional file 1: Figure S8) when the assay was conducted with dilution to 2 anti-RBD BAU/mL total antibody level.

Since ECV19 entered clinical testing, multiple waves of variants have emerged, notably the Delta and Omicron waves. The ability of ECV19 post-immune sera to neutralize those variants of concern was assessed in a microneutralization assay. Against the parent Wuhan strain, FRNT and microneutralization titers correlated well (Additional file 1: Figure S9A, *p* < 0.0001). In the assay conditions, ECV19 induced comparable neutralizing antibodies to the parental strain (geometric mean titer of 117) and Delta strain (geometric mean of 171, *p* = 0.86), Additional file 1: Figure S9B). On the other hand, neutralization of Omicron was substantially diminished compared to either of the other two strains (geometric mean of 24, *p* < 0.0001).

## Discussion

The design of COVID-19 vaccines and clinical trials is rapidly evolving in face of pre-existing vaccination, widespread infection, and new variants. The interim portion of this phase 2 trial evaluation of ECV19 was carried out in South Korea between July and October 2021. Six-month and 12-month follow-up safety and immunogenicity analysis is pending and is not reported here. At the time of immunization and initial sera collection (which forms the basis of the analysis reported here), South Korea had a relatively low cumulative SARS-CoV-2 infection rate of ~ 0.5% (compared to a year later, when the cumulative infection rate reached 40%) [17].

Overall, the solicited adverse events of ECV19 were not particularly unusual and the vaccine was generally well-tolerated. Both high-dose and low-dose ECV19 solicited more low-grade injection site pain and tenderness (local) and low-grade myalgia (only after 1st dose, systemic) adverse events than the placebo group. Those were the only solicited adverse events that were significantly more frequent in the vaccine groups relative to the placebo arm. Local adverse events were more common than systemic ones. Most adverse events resolved within a couple of days. A recent meta-analysis of adverse events in two-dose COVID-19 vaccines (including mRNA-, protein-, and vector-based vaccines) and their placebos was reported, along with associated confidence intervals (CI) [18]. For the placebo group, the percentage of participants experiencing any local AE was reported as 16.2 (95% CI: 11.3 to 21.1) and 11.8 (95% CI: 6.6 to 17.1) after first and second doses, respectively, and those for any systemic AE was 35.2 (95% CI: 26.7 to 43.7) and 31.8 (95% CI: 28.7 to 35.0). As expected, the vaccine group experienced more AE overall; the percentage of any local AE was 66.7 (95% CI: 53.2 to 80.3) after 1st and 72.8 (95% CI: 57.4 to 88.2) after 2nd doses, and that for any systemic AE was 46.3 (95% CI: 38.2 to 54.3) after 1st and 61.4 (95% CI: 47.4 to 75.4) after 2nd doses. In general, those numbers were similar to the proportions of AEs seen in this ECV19 study for any combination of the placebo or either vaccine arm, after first or second injection, for local or systemic adverse events. However, in this study, adverse events were not higher with the second ECV19 injection, which bodes well for future possibilities of multiple boosting shots with this vaccine platform technology. One limitation of this study is that how the various components of ECV19 (e.g., EcML, RBD, CoPoP, other lipids, etc.) individually contributed to the solicitation of adverse events was not assessed.

ECV19 induced strong RBD-specific immune responses. Serological analysis revealed dose-dependent and boosting-dependent antibody responses among both high- and low-dose ECV19 groups. Although an obvious increase in antigen-specific antibodies occurred following boosting, it should be noted that there was no control group that received a single vaccine injection; therefore, it is not possible to rule out that antibodies may have increased from day 21 to day 42 without the boost. The use of the compact RBD antigen led to an immune response that focused antibody response specifically on the RBD more so than whole Spike vaccines. RBD antibody levels correlated with whole Spike antibodies and virus neutralization. T cell responses assessed with a subset group revealed that the high-dose ECV19 induced significant higher IFN- γ responses with IL-4 responses trending higher. A further study with a larger sample size is required to fully assess the cellular immunity induced by ECV19 vaccine.

As is the case with COVID-19 vaccine development, it is difficult for vaccine design to keep up with emergent strains. ECV19 was designed and the trial approved prior to the emergence of SARS-CoV-2 variants, including the Delta and Omicron strains, the latter which features a highly mutated RBD. Cursory examination of a subset of the trial samples showed that ECV19 post-immune sera maintained comparable neutralization efficacy against Delta, but Omicron levels were approximately sixfold lower, which is consistent with the literature values for Omicron [19]. In the future, ECV19 could be updated to better match the RBD of the current circulating SARS-CoV-2 strain. This should be feasible since the second dose was well-tolerated, showing no escalation in adverse events upon boosting. Furthermore, the liposome scaffold itself is protein-free and antibodies against the short his-tag were not detected. The performance of ECV19 in phase 3 trials now being carried out, along with the variant landscape once those results are available, could influence whether an Omicron-specific booster would be developed in the future.

Several limitations of this study should be noted. The ratio of females was higher in the high-dose ECV19 group compared to the low-dose ECV19 group. Based on meta-analysis studies, in the case of COVID-19 vaccines, women tend to show the same or slightly better vaccine efficacy than men and also report more adverse reactions [20–22]. This study also showed higher immunogenicity in females than male; thus, a part of higher titers in high-dose ECV19 group compared to low-dose group might partially be explained by the different sex ratio (although there were significant differences in immunogenicity between high- and low-dose groups in each sex on day 42). For the adverse reactions, since overall adverse events of this study were low grade and tentative, we did not further investigate sex differences, which will be better evaluated in a larger phase 3 study. Another limitation of this study report is that it is only an interim analysis and immunogenicity was only assessed until the day 42 time point. Further serum collection and analysis is planned at the 6- and 12-month follow-up and will be interesting to determine how antibody ELISA and FRNT values change in that period. Finally, T cell responses were evaluated only from a limited subset of volunteers, although cellular immunity also plays an important role against SARS-CoV-2.

## Conclusions

ECV19 represents a novel vaccine technology that involves antigen display on the surface of immunogenic liposomes. This is enabled by combining EcML and CoPoP liposomal technologies. As such, it is significant that this phase 2 clinical trial demonstrated that ECV19 was well-tolerated and induced functional antibodies in a dose- and boosting-specific manner. No increased adverse events at the boosting immunization suggest potential compatibility for incorporation into future multi-injection boosting regimes. An ongoing phase 3 trial (ClinicalTrials.Gov Identifier: NCT05572879) should provide further insight into the efficacy of this unique nanoparticle-based vaccine approach.

## Supplementary Information

**Additional file 1: Table S1.** Exclusion criteria. **Table S2.** Schedule of safety and immunological assessment. **Fig S1.** RBD antigen used. **Fig S2.** Induction of neutralizing antibodies by ECV19. **Fig S3.** Immunogenicity by site comparison. **Fig S4.** Effect of age and sex on immunogenicity. **Fig S5.** Anti-RBD ratio of ECV19 compared to other vaccines. **Fig S6.** Antigen specific T cell response. **Fig S7.** EVC19 did not induce detectable level of anti-His antibody responses. **Fig S8.** Subclass analysis. **Fig S9.** Impact of ECV19 on variants of concern.

**Additional file 2. **Clinical protocol.

## Data Availability

All data are available from the authors upon reasonable request.

## Abbreviations

AE: Adverse event
BAU: Binding antibody unit
CI: Confidence interval
COVID-19: Coronavirus disease 2019
CoPoP: Cobalt porphyrin-phospholipid
EcML: *Escherichia Coli*-produced monophosphoryl lipid A
ECV19: EuCorVac-19
ELISA: Enzyme-linked immunosorbent assay
FRNT: Focus reduction neutralization test
IFNγ: Interferon-gamma
IgG: Immunoglobulin G
IL-4: Interleukin-4
IWRS: Interactive Web Response System
MNA: Microneutralization assay
mRNA: Messenger ribonucleic acid
PBMC: Peripheral blood mononuclear cell
RBD: Receptor-binding domain
S: Spike
SARS-CoV-2: Severe acute respiratory syndrome coronavirus 2
SFC: Spot forming cells
WHO: World Health Organization
