# Supplementary material for: Interim analysis from a phase 2 randomized trial of EuCorVac-19: a recombinant protein SARS-CoV-2 RBD nanoliposome vaccine
Source: BMC Med. 2022 Nov 30;20:462. doi: 10.1186/s12916-022-02661-1 (PMC9708508; doi:10.1186/s12916-022-02661-1)
Supplement: Supplementary file 1 — Additional file 1: Table S1. Exclusion criteria. Table S2. Schedule of safety and immunological assessment. Fig S1. RBD antigen used. Fig S2. Induction of neutralizing antibodies by ECV19. Fig S3. Immunogenicity by site comparison. Fig S4. Effect of age and sex on immunogenicity. Fig S5. Anti-RBD ratio of ECV19 compared to other vaccines. Fig S6. Antigen specific T cell response. Fig S7. EVC19 did not induce detectable level of anti-His antibody responses. Fig S8. Subclass analysis. Fig S9. Impact of ECV19 on variants of concern. [file 12916_2022_2661_MOESM1_ESM.pdf]

**Supplementary data: Interim Analysis from a Phase 2 Randomized Trial of EuCorVac-19; a Recombinant Protein SARS-CoV-2 RBD Nanoliposome Vaccine.**

**Table S1: Exclusion criteria**

| | |
|---|---|
| **1** | COVID-19 positive based on RT-PCR diagnosis with upper respiratory tract(nasopharyngeal, oropharyngeal) or lower respiratory tract(sputum) sampling or COVID-19 antibody (Immunoglobulin M [IgM] and/or Immunoglobulin G [IgG]) positive. |
| **2** | History of SARS-CoV, MERS-CoV or SARS-CoV-2 infection. |
| **3** | Increased risk of exposure to SARS-CoV-2 (e.g., healthcare workers in direct contact with patients with COVID-19). |
| **4** | History of vaccination against SARS-CoV, MERS-CoV, or SARS-CoV-2. |
| **5** | Immune system disorders including immunodeficiency disease. |
| **6** | Planned blood donation or transfusion during the study period. |
| **7** | Planned administration of other vaccines from 4 weeks before the 1st dose to 4 weeks after the 2nd dose of the IP. |
| **8** | Height and/or weight measurements at Screening:<br>① Body weight <40 kg or ＞100 kg.<br>② Body mass index <18 kg/m2 or ＞30 kg/m2. |
| **9** | Clinically significant abnormalities in clinical laboratory tests, ECGs and chest X-ray during Screening in the opinion of the Investigator (e.g., Wolff-Parkinson-White syndrome). |
| **10** | Any planned surgery during the study period. |
| **11** | Fever (≥37.5°C) within 3 days prior to Screening or serious acute (acute fever, cough, respiratory distress, chills, myalgia, headache, sore throat, anosmia, or ageusia) or chronic infection within 7 days prior to screening requiring systemic antibiotics or antivirals. |
| **12** | Evidence or history of serious acute, chronic, or progressive disease (e.g., cancer, diabetes, chronic pulmonary disease, acquired immune deficiency syndrome(AIDS), blood dyscrasias, or immune system, urinary system, mental, musculoskeletal system, cardiovascular system, respiratory system, endocrine, nervous system, hepatobiliary system, or renal disorders, etc.) which, in the opinion of the Investigator, makes the individual ineligible for the study. |
| **13** | Positive serum tests during screening (type B hepatitis, human immunodeficiency virus [HIV], type C hepatitis). |
| **14** | History of treatment with antipsychotics or opioid analgesic dependence within 6 months prior to IP dosing. |
| **15** | History of severe allergic reactions (e.g., anaphylaxis, Guillain-Barré Syndrome) or severe hypersensitivity reactions to the IP or any of its components. |
| **16** | History of therapy that might affect immunity: treatment with immunosuppressants or immune modifying drugs, anticancer therapy, or radiotherapy within 3 months prior to screening. |
| **17** | History of systemic steroids (prednisone ≥10mg/day for ＞14 consecutive days) within 3 months prior to screening. Topical, inhaled, and intranasal corticosteroids are allowed regardless of dose. |
| **18** | Past treatment within 3 months prior to screening, or planned treatment during the study period, with immunoglobulin or blood derivatives. |
| **19** | Individual with thrombocytopenia or other coagulation disorders for whom intramuscular (IM) injections are contraindicated or individual who is on anticoagulant therapy.*<br>* Anticoagulant therapy: continuous use of anticoagulants such as coumarin/warfarin or new oral anticoagulants/antiplatelets. |
| **20** | History of excessive alcohol consumption or drug addiction. |
| **21** | Women of childbearing potential who do not agree to use a medically accepted contraception* or to be heterosexually inactive for up to 60 days after the last dose of IP |

| | |
|---|---|
| | * Hormonal contraceptive, intrauterine device (IUD) or intrauterine system (IUS), tubal ligation, double-blocking method (condom for male and female, cervical cap or diaphragm, complex method such as contraceptive sponge), single-blocking method using spermicide Pregnant or lactating women |
| **22** | Treatment with other IPs within 6 months prior to participation in this study. |
| **23** | The Investigator who is directly related to this study or sub-Investigator/study coordinator who is supervised by the Investigator or their family member. |
| **24** | Other reasons including medical reasons based on which the individual is considered to be ineligible for this study in the opinion of the Investigator. |

**Table S2: Schedule of safety and immunological assessment**

| | **Screening** | **Baseline / 1st dose** | | | **2nd dose** | | | |
|---|---|---|---|---|---|---|---|---|
| Visit | V1 | V2 | V3 | V4 | V5 | V6 | V7 | V8[a] |
| Study day | -7 to -1 | 0 | 2 to 5 | 7 | 21 | 23 to 26 | 28 | 42 |
| AE checking | | X | X | X | X | X | X | X |
| Vital signs | X | X | | X | X | | X | X |
| Physical examination | X | X | | X | X | | X | X |
| Clinical laboratory test[b] | X | | | X | | | X | |
| Telephone monitoring | | | X | | | X | | |
| Subject diary retrieving | | | | X | X | | X | X |
| ECG and chest X-ray | X | | | | | | | |
| Pregnancy test | X | | | | | | | |
| Medical and Surgical history | X | X | | | | | | |
| COVID-19 test | X | X | | | | | | |
| Immunological assessment | | X | | | X | | | X |

[a] Three more visits were planned at study days 49 (V9), 203 (V10) and 385 (V11). Analyses for those have not yet been completed and are not included in this report.

[b] Clinical laboratory tests included hematology (RBC, hemoglobin, hematocrit, platelets count, WBC and WBC differential count), blood chemistry (glucose, BUN, creatinine, uric acid, total protein, total bilirubin, albumin, ALP, ALT, AST, γ-GT, total cholesterol, Ca, Na, P, Cl and K), urinalysis (albumin, glucose, WBC, ketone and RBC), blood coagulation (PT and aPTT), and virus test (HBs Ag, HIV Ag/Ab and HCV Ab). The blood coagulation and virus tests were performed only at Visit 1.

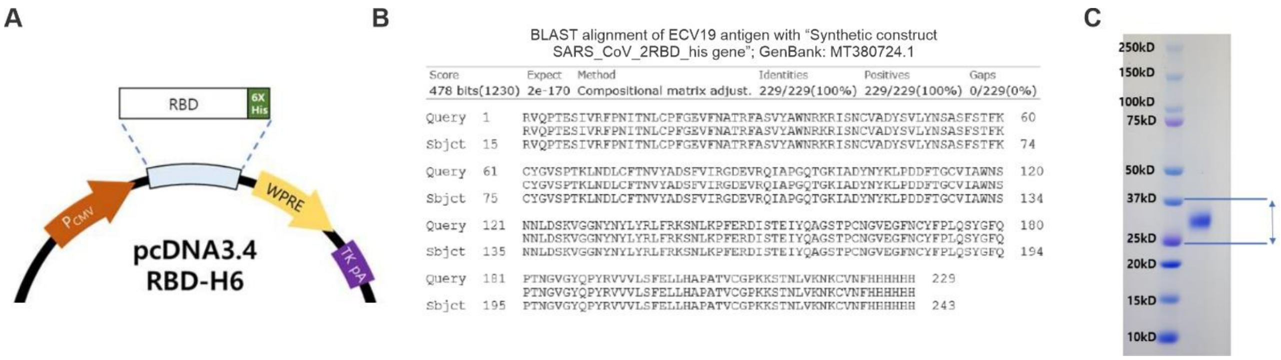


**Figure S1: RBD antigen used. A)** Plasmid and cloning strategy to generate a stable CHO cell line expressing the **B)** his-tagged RBD sequence resulting for generating the **C)** purified recombinant his-tagged RBD visualized with SDS-PAGE and Coomassie staining.

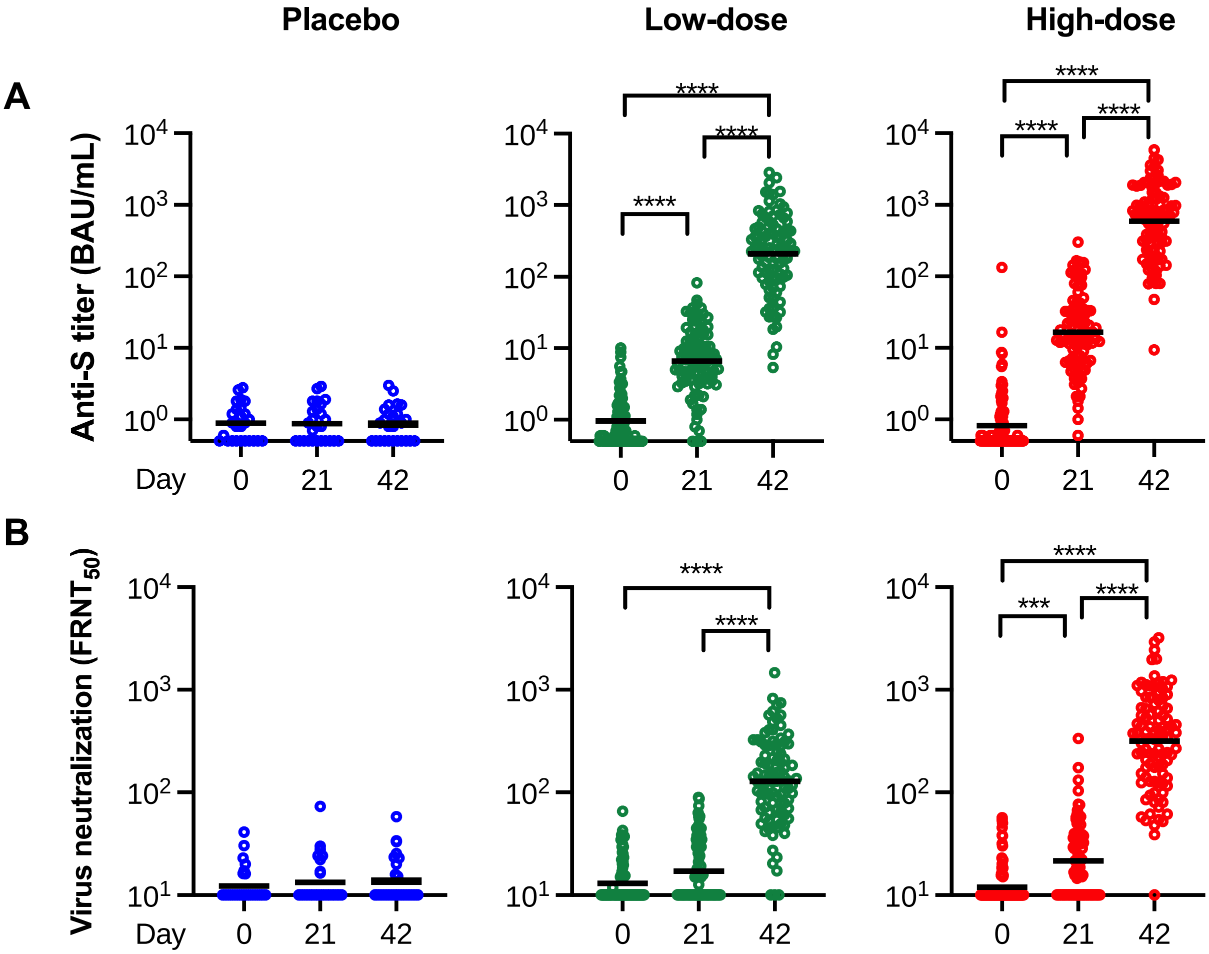


**Figure S2: Induction of neutralizing antibodies by ECV19 .** Figure 4 data were replotted by immunization groups, instead of study days. Indicated **** and *** correspond to P values of < 0.0001 and < 0.001, respectively.

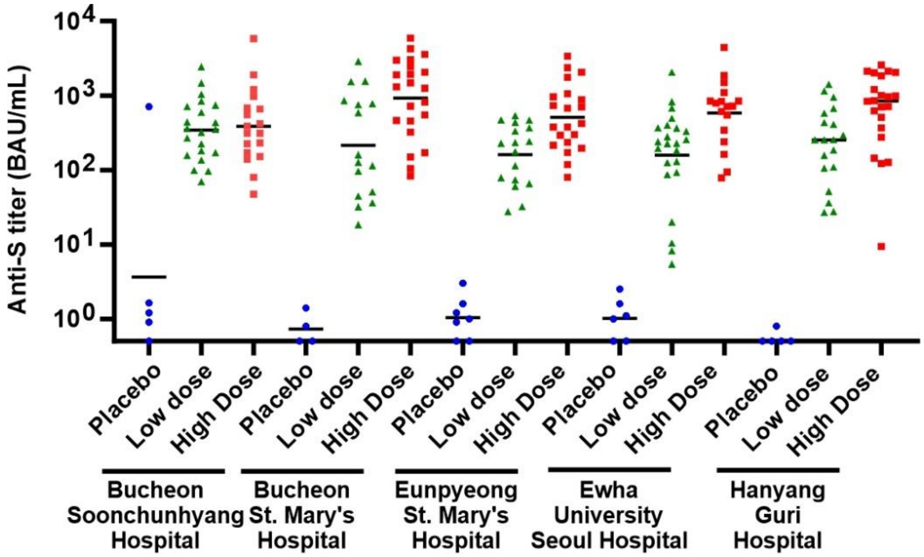


**Figure S3: Immunogenicity by site comparison.** No significant differences between the different sites was observed by Kruskal-Wallis multiple comparison test.

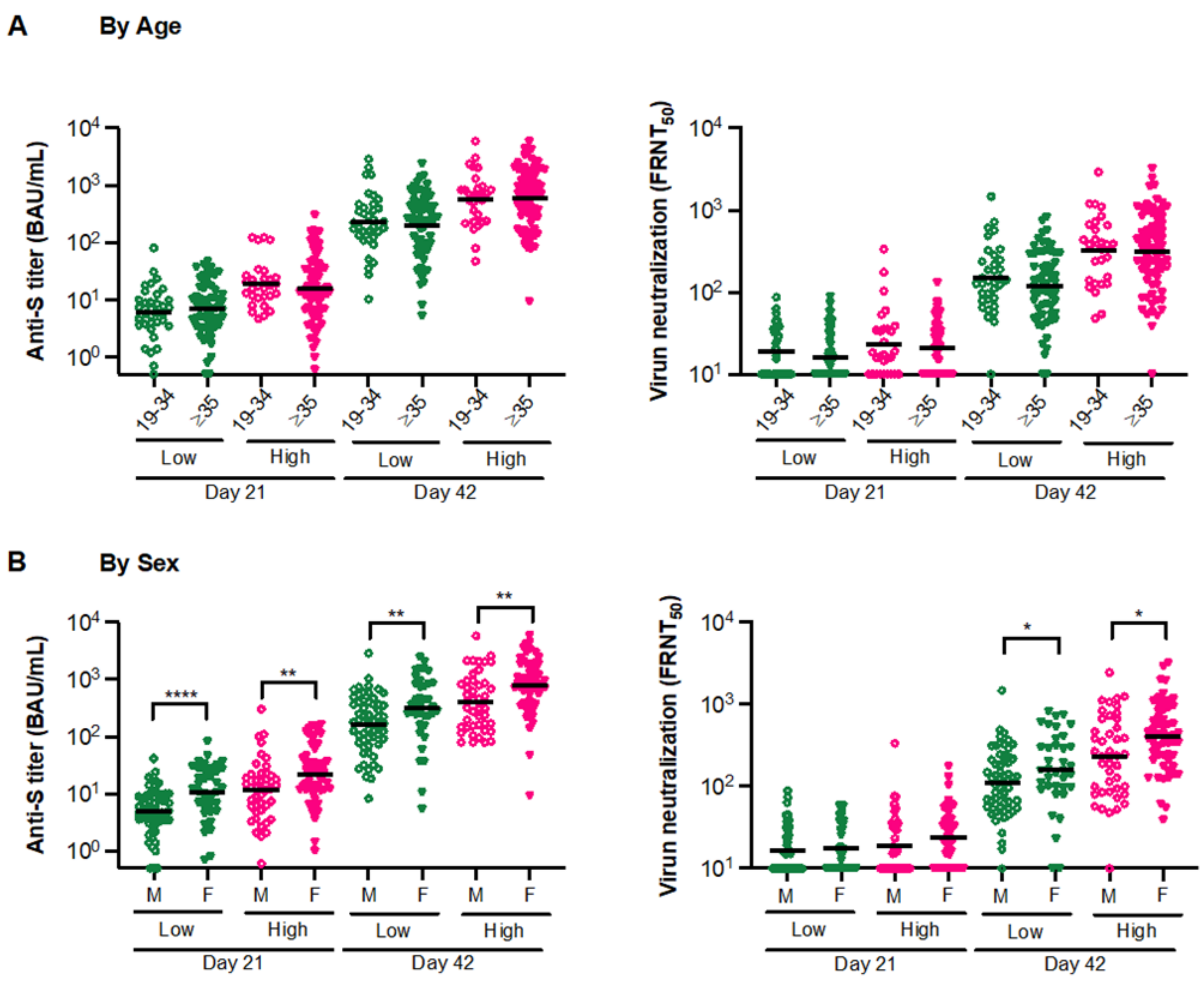


**Figure S4: Effect of age and sex on immunogenicity**. Low- and High-dose group data shown in Figure 4 were replotted by age (**A**) or sex (**B**). Indicated ****, ** and * correspond to P values of $< 0.0001$, $<0.01$ and $< 0.05$, respectively, based on Mann-Whitney test.

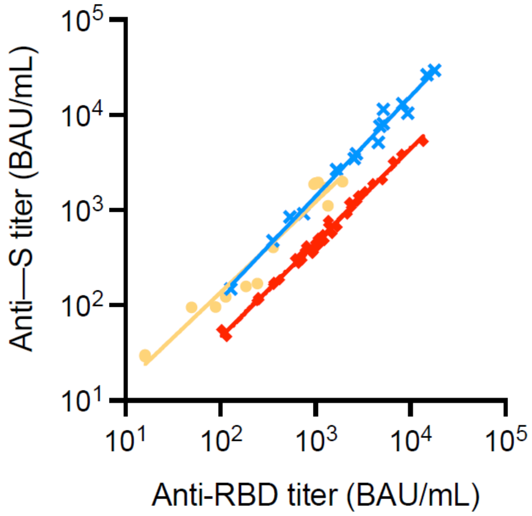


**Figure S5: EVC19 induces a higher ratio of anti-RBD to anti-S antibodies, compared to JnJ and Pfizer sera.** The anti-RBD and anti-S antibody levels of post-immune ECV19 (red, both high and low-dose groups, n=38), Pfizer (blue, n=15), JnJ (yellow, n=14) were plotted with the best-fit lines ECV19, each of three data set fits linear regressions well ($R^2 > 0.95$).

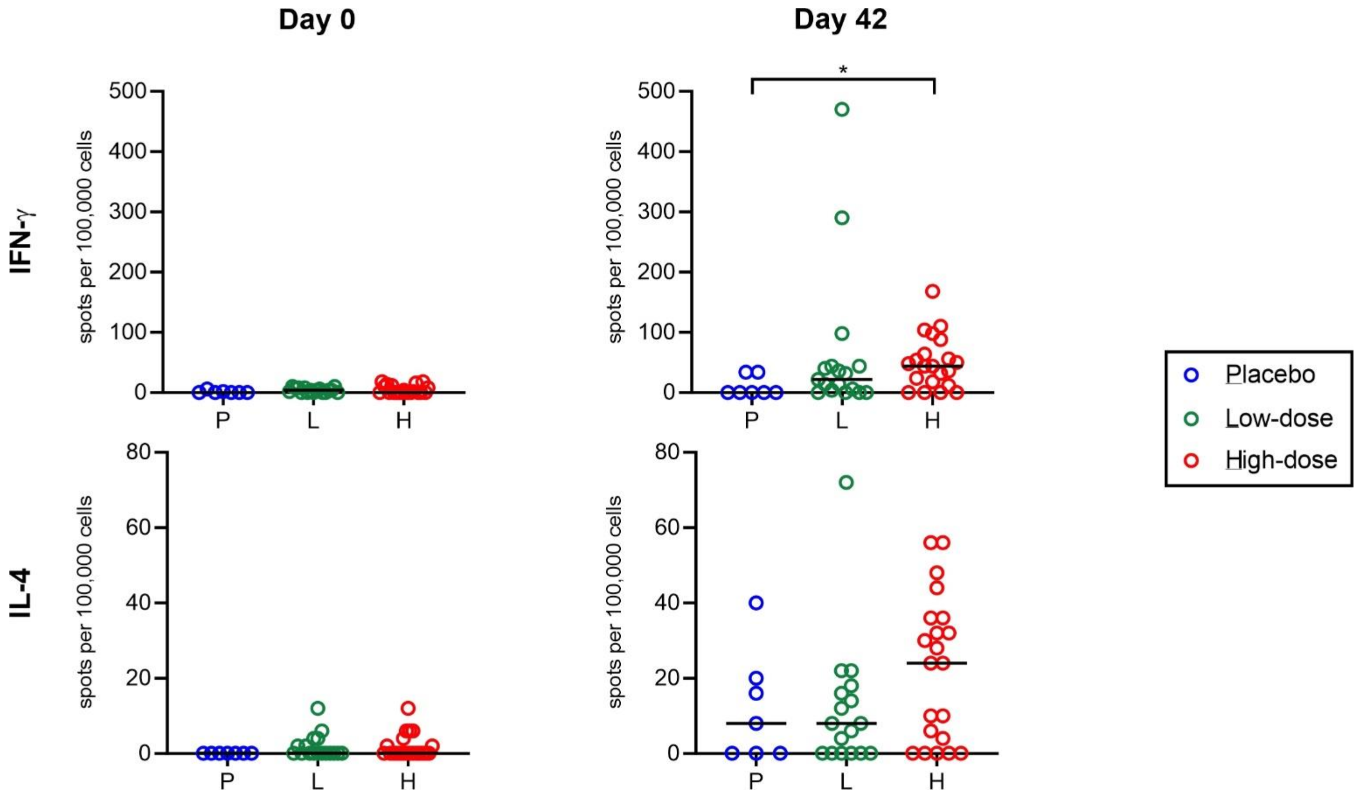


**Figure S6: Antigen specific T cell response.** The T cell immune response index (spot forming counts per $10^5$ cells) in peripheral blood cells was analyzed by IFN-γ or IL-4 ELISpot. A subset of participants was assessed for this exploratory analysis (n=7 in the placebo, n=17 in low-dose ECV19, and n=21 in high-dose ECV19). Lines show median and P < 0.5 (*), based on Kruskal–Wallis and Dunn's test .

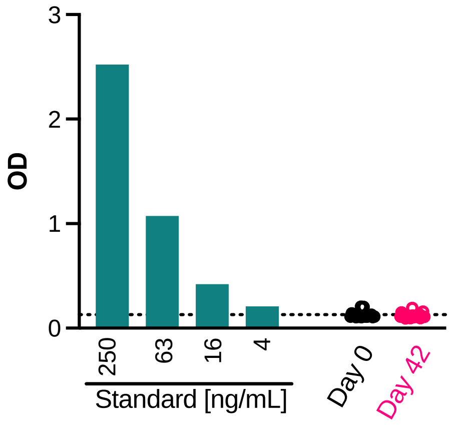


**Figure S7: EVC19 did not induce detectable level of anti-His antibody responses**. Days 0 and 42 sera from high-dose group (n=21 each) collected at Eunpyeong St. Mary's Hospital were tested at 1:200 dilution using ELISA plates coated with 200 ng/well of 10 x His peptide. As a standard, anti-His-Tag chimeric human monoclonal antibody (Sigma, Catalog # SAB5600096) was included at indicated concentrations. The dotted line shows average OD value of blank wells (OD=0.128).

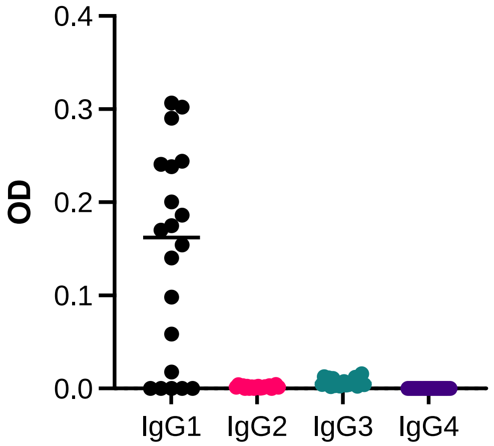


**Figure S8: Subclass analysis.** Day 42 sera from high-dose ECV19 group collected at Eunpyeong St. Mary's Hospital (n=20, one sample was not tested due to the limitation of available) were diluted to 2 anti-RBD BAU/mL total antibody, then IgG subclass responses were determined using subclass-specific secondary antibodies. Individual (dot) and median (bar) values are shown.

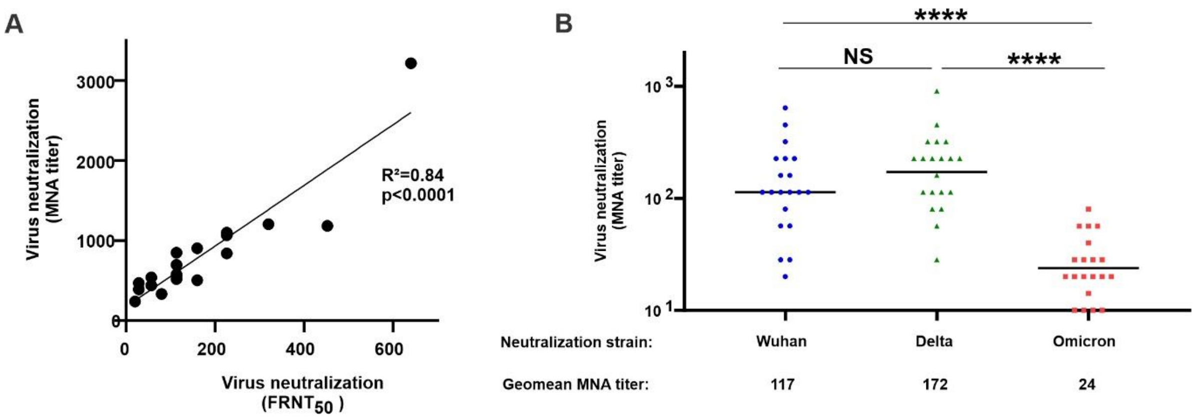


**Figure S9: Impact of ECV19 on variants of concern.** 20 high-dose ECV19 post-immune sera samples were tested by MNA. **A)** Correlation of FRNT and MNA values. Pearson correlation P value is shown. **B**) Neutralization of indicated strains by ECV19 post-immune sera. NS = not significant; ****, P<0.0001; by Kruskal-Wallis test followed by Dunn's multiple comparison test.
