# Supplementary material for: Interim analysis from a phase 2 randomized trial of EuCorVac-19: a recombinant protein SARS-CoV-2 RBD nanoliposome vaccine
Source: BMC Med. 2022 Nov 30;20:462. doi: 10.1186/s12916-022-02661-1 (PMC9708508; doi:10.1186/s12916-022-02661-1)
Supplement: Supplementary file 2 — Additional file 2. Clinical protocol. [file 12916_2022_2661_MOESM2_ESM.pdf]

Clinical Trial Protocol

A phase I/II dose-exploration, randomized, observer-blind, placebo-controlled study to determine safety, tolerance and immunogenicity of EuCorVac-19, a recombinant protein vaccine, for the prevention of COVID-19 in healthy adults

**Protocol No.:**	EuSNAP_COV101
**Development phase:**	Phase 1/2
**Test drug:**	EuCorVac-19
**Target indication:**	Prevention of COVID-19 (SARS-CoV-2)
**Sponsor:**	EuBiologics Co., Ltd.<br>8th Floor, Seongdo Building, 207, Dosandae-ro, Gangnam-gu, Seoul, South Korea
**Protocol version:**	Version 2.1 dated 09Feb2021

Confidential

This document is the property of EuBiologics Co., Ltd. and may not – in full or in part – be passed on, reproduced, published or otherwise used without the express permission of EuBiol ogics Co., Ltd..

▣ Document revision history

No.	Version No.	Version date	Major changes
1	1.0	11Dec2020	Original protocol
2	2.0	08Jan2021	As per the request of MFDS, the following items were amended.<br>1. Change of title<br>2. [Section 2] Change of study objectives<br>3. [Section 3.1] change of study design(addition of placebo group and procedures for safety evaluation, clarification on initiation procedure of Part B and addition of Schedule of Assessment)<br>4. [Section 4.1.2] Specified immunity test methods<br>5. [Section 5.1, 5.2] Change of inclusion/exclusion criteria<br>6. [Section 5.4] Addition of phrase on the recording of reasons and actions taken for the discontinuation and withdrawal on relevant document and CRF<br>7. [Section 5.5] Change of study suspension criteria<br>8. [Section 6.1.1] Specified the volume of vial<br>9. [Section 6.2] Specified the details on the administration of placebo, dosage and discard of remaining investigational drug<br>10. [Section 7.1] Change of sample size based on the placebo group and drop-out rate<br>11. [Section 7.2.2] Addition of humoral immune response as an analysis item and change of schedule of cell-mediated immune response analysis<br>12. [Section 7.3.1] Change of definition of FAS analysis group<br>13. [Section 6.5, 7.3.2] Change on unblinding after primary analysis<br>14. [Section 8.2] Addition of reporting procedure of adverse effect above Grade 3<br>15. [Attachment 3] Addition of central lab<br>16. Change of visit window<br>17. Addition of attachment(Human-derived material ICF)
3	2.1	09Feb2021	1. [Schedule of Assessment] Addition of pregnancy test at Visit 9(end of main study

			visit)<br>2. [Section 3.3] Specified the number representing dose group in Part A<br>3. [Section 4.2.5] Specified the procedure of drop-out due to contraindication for the 2nd dose<br>4. [Section 5.3] Specified the procedure of subjects drop-out due to contraindication for the 2nd dose<br>5. [Section 7.3.8] Specified the procedure of DSMB opening<br>6. Specification of other phrase and correction of typographical error

Table of contents

▣ **Document revision history** ······ 2

▣ **Glossary of terms and abbreviations** ······ 8

▣ **Synopsis** ······ 11

▣ **Schedule of Assessments (Part A & B)** ······ 21

1. Introduction ······ 25

1.1 Background of study ······ 25
1.2 Study rationale ······ 26
1.2.1 Rationale for study design ······ 26
1.2.2 Rationale for dose selection ······ 27
1.2.3 Potential risk-benefit assessment ······ 27

2. Study objectives ······ 29

2.1 Part A ······ 29
2.2 Part B ······ 29

3. Overall study design and plans ······ 30

3.1 Study design ······ 30
3.1.1 Part A ······ 30
3.1.2 Part B ······ 31
3.2 Participating institutions ······ 32
3.3 Assigning to treatment groups and assigning procedures ······ 32
3.3.1 Part A ······ 32
3.3.2 Part B ······ 32

4. Study assessment methods and procedures ······ 34

4.1 Assessment methods ······ 34
4.1.1 Safety assessment ······ 34
4.1.2 Immunogenicity assessment ······ 35

4.1.3 Others ············ 38
4.2 Procedures by visit ············ 41
4.2.1 Visit 1 (screening, Day -7 ~ Day -1) ············ 41
4.2.2 Visit 2 (1st dose of the IP, Day 0) ············ 42
4.2.3 Visit 3 (telephone visit, 2 ~ 5 days after the 1st dose) ············ 43
4.2.4 Visit 4 (interim visit for the main study, 7 + 3 days after the 1st dose) ············ 43
4.2.5 Visit 5 (2nd dose of the IP, 21 + 5 days after the 1st dose) ············ 43
4.2.6 Visit 6 (telephone visit, 2 ~ 5 days after the 2nd dose) ············ 44
4.2.7 Visit 7 (interim visit for the main study, 7 + 3 days after the 2nd dose) ············ 44
4.2.8 Visit 8 (interim visit for the main study, 21 + 5 days after the 2nd dose) ············ 45
4.2.9 Visit 9 (end of main study visit, 28 + 7 days after the 2nd dose) ············ 45
4.2.10 Visit 10 (interim visit for follow-up, 26 ± 2 weeks after the 2nd dose) ············ 46
4.2.11 Visit 11 (end of follow-up visit, 52 ± 4 weeks after the 2nd dose) ············ 46
4.2.12 Unscheduled visits ············ 46

5. Subject selection and withdrwawl criteria ············ 47

5.1 Inclusion criteria ············ 47
5.2 Exclusion criteria ············ 47
5.3 Contraindications for the 2nd dose ············ 49
5.4 Criteria for subject discontinuation and withdarwawl ············ 49
5.5 Criteira for study suspension ············ 50
5.6 Study compliance and handling of protocol deviations ············ 51

6. Investigational product ············ 52

6.1 Overview of investigational product ············ 52
6.1.1 Test drug ············ 52
6.1.2 Placebo (for Part B only) ············ 53
6.2 Dosage, method, and duration of treatment ············ 54
6.3 Manufacturing, packaging, and labeling of the investigational product ············ 54
6.4 Management of the investigational product ············ 55
6.5 Mantaining and breaking the blind ············ 55
6.6 Concomitant medication and concomitant therapy ············ 56

7. Statistical considerations ············ 58

7.1 Sample size and rationale for sample size calcuiaton 58
7.1.1 Sample size 58
7.1.2 Rationale for sample size calculation 58
7.1.3 Replacement of Study Subject 59
7.2 Endpoints 59
7.2.1 Safety endpoints 59
7.2.2 Immunogenicity endpoints 59
7.3 Statistical analysis plan 61
7.3.1 Analysis sets 61
7.3.2 General principles of statistics 62
7.3.3 Analysis of demographics and baseline characteristics 62
7.3.4 Safety analysis (Part A & Part B) 62
7.3.5 Immunogenicity analysis 63
7.3.6 Handling of drop-outs or missing data 66
7.3.7 Planned interim analysis and safety monitoring 66
7.3.8 Data and safety monitoring board 67

8. Adverse events 69

8.1 Definition of adverse events 69
8.2 Collection and documentation of adverse events 70
8.3 Assessment of adverse events 70
8.3.1 Severity assessment 71
8.3.2 Assessment of causal relationship 75
8.4 Reporting serious adverse events 77
8.5 Reporting suspected unexpected serious adverse reactions and actions to be taken 77
8.6 Pregnancy 78

9. Ethical considerations and administrative procedures 79

9.1 Protocol compliance 79
9.2 Protocol approval and amendment 79
9.3 Consenting procedures 79
9.4 Measures to protect the safety of subjects 80
9.5 Measures to protect vulnerable subejcts for study enrollment 80
9.6 Criteria for post-study medical examination and treatment for subjects 81
9.7 Indemnification provisions for subjects 82

9.8 Study related documents and record keeping ··· 82
9.8.1 Case report forms and source documents ··· 82
9.8.2 Access to source documents ··· 82
9.8.3 Archiving study data ··· 83
9.8.4 Audits and inspections ··· 83
9.9 Confidentiality of study documents and subject records ··· 84
9.10 Monitoring of institutions ··· 84
9.11 Early termination or suspension of the study ··· 84
9.11.1 Sponsor ··· 84
9.11.2 Investigator ··· 85
9.12 Clinical study report and publication ··· 85

10. References ··· 87

11. List of attachments ··· 89

▣ Glossary of terms and abbreviations

Ab	: Antibody
ACE2	: Angiotensin-Converting Enzyme 2
ADE	: Antibody-Dependent Enhancement
ADR	: Adverse Drug Reaction
AE	: Adverse Event
AESI	: Adverse Events of Special Interest
AIDS	: Acquired Immune Deficiency Syndrome
ALT	: Alanine Aminotransferase
ALP	: Alkaline phosphatase
AST	: Aspartate Aminotransferase
aPTT	: Activated Partial Thromboplastin Time
BCIP/NBT	: 5-bromo-4-chloro-3-indolyl phosphate/nitro blue tetrazolium
BMI	: Body Mass Index
BUN	: Blood Urea Nitrogen
CRF	: Case Report Form
CRO	: Contract Research Organization
CoV	: Coronavirus
CoPoP	: Cobalt-Porphyrin-Phospholipid
COVID-19	: Coronavirus Disease-19
CTL	: Cellular Technology Limited
DNA	: Deoxyribo Nucleic Acid
DOPC	: Dipalmitoylphosphatidylcholine
DSMB	: Data and Safety Monitoring Board
ECG	: Electrocardiogram
EcML	: E. coli produced Monophosphoryl Lipid A
e-CRF	: Electronic-Case Report Form
EDC	: Electronic Data Capture
ELISA	: Enzyme-Linked Immunosorbent Assay
ELISpot	: Enzyme-Linked Immune absorbent Spot
ERD	: Enhanced Respiratory Disease
FAS	: Full Analysis Set
FcγR	: Fc-gamma Receptor
FCS	: Fetal Calf Serum
FDA	: Food and Drug Administration

FRNT	: Focus Reduction Neutralization Test
GCP	: Good Clinical Practice
GMFR	: Geometric Mean Fold Rise
GMT	: Geometric Mean Titer
HBsAg	: Hepatitis B Virus Surface Antigen
hCG	: Human Chorionic Gonadotropin
HIV	: Human Immunodeficiency Virus
HRP	: Horseradish Peroxidase
IgG	: Immunoglobulin G
IFNγ	: Interferon gamma
IL-2, IL-4, IL6	: Interleukin-2, Interleukin-4, Interleukin-6
IRB	: Institutional Review Board
IUD	: Intrauterine device
IUS	: Intrauterine system
IWRS	: Interactive Web Response System
MedDRA	: Medical Dictionary for Regulatory Activities
MERS	: Middle East Respiratory Syndrome
NP	: Nucleocapsid Protein
PBMC	: Peripheral Blood Mononuclear Cells
pH	: Negative logarithm of the hydrogen ion concentration
PPS	: Per Protocol Set
PT	: Preferred Term
PT	: Prothrombin Time
RBC	: Red Blood Cell
RBD	: Receptor-Binding Domain
RNA	: RiboNucleic Acid
RPMI	: Roswell Park Memorial Institute
RSV	: Respiratory Syncytial Virus
RT-PCR	: Reverse Transcription-Polymerase Chain Reaction
SAE	: Serious Adverse Event
SARS	: Severe Acute Respiratory Syndrome
SAS	: Statistical Analysis System
SCR	: Seroconversion Rate
SFC	: Spot Forming Cell
SOC	: System Organ Class

SOP	: Standard Operating Procedure
SUSAR	: Suspected Unexpected Serious Adverse Reaction
Th2	: Type 2 Helper T
TNF-α	: Tumor Necrosis Factor-α
TLR4	: Toll-like receptor 4
TMB	: Tetramethylbenzidine
WBC	: White Blood Cell
WHO	: World Health Organization

▣ Synopsis

**Title of study**	A phase I/II dose-exploration, randomized, observer-blind, placebo-controlled study to determine safety, tolerance and immunogenicity of EuCorVac-19, a recombinant protein vaccine, for the prevention of COVID-19 in healthy adults
**Sponsor**	EuBiologics Co., Ltd.<br>8th Floor, Seongdo Building, 207, Dosandae-ro, Gangnam-gu, Seoul, Republic of Korea
**Institutions and principal investigators**	Coordinating investigator: Professor Jeong-Hyeon Choi, Department of Infectious Diseases, the Catholic University of Korea Eunpyeong St. Mary's Hospital<br><br>For the full list of institutions, see Attachment 3. 'Study institutions and the sponsor's organizations'.
**Duration of study**	18 months from the approval of the Institutional Review Board
**Target indication**	Prevention of Coronavirus Disease-19 (COVID-19; SARS-CoV-2)
**Study objectives**	• **Part A**<br>1) Primary Objective<br>To evaluate the safety and tolerability of EuCorVac-19 at all dose levels in healthy adults aged 19 to 50.<br><br>2) Secondary Objective<br>To evaluate the immune response of EuCorVac-19 at all dose levels in healthy adults aged 19 to 50.<br><br>• **Part B**<br>1) Primary Objective<br>To evaluate the immune response of EuCorVac-19 against SARS-COV-2 in healthy adults aged 19 to 75.<br><br>2) Secondary Objective<br>To evaluate safety and tolerability of EuCorVac-19 against SARS-CoV-2 in healthy adults aged 19 to 75.
**Development phase and study design**	• **Part A:** dose-escalation, single-center, randomized, observer-blind, placebo-controlled Phase 1 study

- **Part B:** multi-center, randomized, observer-blind, placebo-controlled Phase 2 study

Study methodology

This study consists of two parts: Part A to evaluate the safety and tolerability of EuCorVac-19 in healthy adults and Part B to evaluate the safety, tolerability, and immune response of EuCorVac-19 in healthy adults.

Amongst individuals who voluntarily provide written informed consent to participation in this study, those eligible based on the inclusion/exclusion criteria will be randomized to a dose level to receive two doses of the investigational product (IP) with 21 days apart and be assessed for safety, tolerability, and immunogenicity according to the following schedules. The study schedules for both Part A and B are same.

<table>
<tr><th>Visit 1</th><th>Visit 2</th><th>Visit 3</th><th>Visit 4</th><th>Visit 5</th><th>Visit 6</th><th>Visit 7</th><th>Visit 8</th><th>Visit 9</th><th>Visit 10</th><th>Visit 11</th></tr>
<tr><td>Day -7~-1</td><td>Day 0</td><td>Day 2~5</td><td>Day 7</td><td>Day 21</td><td>Day 2~5 after the 2nd dose</td><td>Day 7 after the 2nd dose</td><td>Day 21 after the 2nd dose</td><td>Day 28 after the 2nd dose</td><td>Week 26 after the 2nd dose</td><td>Week 52 after the 2nd dose</td></tr>
<tr><td>-</td><td>-</td><td>-</td><td>+3D</td><td>+5D</td><td>-</td><td>+3D</td><td>+5D</td><td>+7D</td><td>±2W</td><td>±4W</td></tr>
<tr><td rowspan="3">Screening</td><td rowspan="2">Randomization 1st dose</td><td colspan="6">Main study</td><td rowspan="2">End of main study visit</td><td rowspan="2">Follow-up interim visit</td><td rowspan="2">End of follow-up visit</td></tr>
<tr><td>Telephone visit</td><td>Interim visit</td><td>2nd dose</td><td>Telephone visit</td><td>Interim visit</td><td>Interim visit</td></tr>
<tr><td>Safety and immunogenicity assessment</td><td colspan="2">Safety assessment</td><td>Safety and immunogenicity assessment</td><td colspan="2">Safety assessment</td><td>Safety and immunogenicity assessment</td><td>Safety assessment</td><td colspan="2">Safety and immunogenicity assessment</td></tr>
</table>

- **Part A**

Part A consists of two dose levels(low dose, high dose) of EuCorVac-19. Each dose group consists of 15 subjects in test group and 10 subjects in placebo group.

The first 5 subjects will be randomized and enrolled to the low dose group (Sentinel group: 3 subjects in Test group, 2 subjects in Placebo group); once the safety of the IP is confirmed in these subjects over 7 days after the 1st dose of the IP, the study will proceed to enroll the rest of 20 subjects to the low dose group. The rest of 20 subjects will be randomized to 12 subjects in Test group and 8 subjects in Placebo group. Also, the first 5 subjects will be randomized and

	enrolled to the high dose group (Sentinel group: 3 subjects in Test group, 2 subjects in Placebo group); If no Grade ≥3 adverse drug reactions (ADRs) are reported within 7 days (Day 0 ~ Day 6) after the 1st dose of the IP, the rest of 20 subjects to the high dose group will be enrolled. The 20 subjects in the high dose group will be randomized to 12 subjects in Test group and 8 subjects in Placebo group.<br><br>If Grade ≥3 ADRs are reported within 7 days after the 1st dose of the IP in any of the first 5 subjects(Sentinel group) in each dose groups, the Data and Safety Monitoring Board (DSMB) will review the safety of the IP and determine whether to continue the study. If applicable, subject enrollment will be stopped and additional dosing in the existing subjects suspended until the decision is made on study continuation.<br><br>Once the safety results up to 28 days after the 2nd dose are collected from all subjects in Part A, the DSMB will review the safety data from all subjects enrolled in the high and low dose groups in Part A. The DSMB will assess if the event of ADRs in all subjects meet the criteria for study suspension and figure out the relationship between ADR and IP, based on which proceeding to Part B will be decided.<br><br>An interim analysis will be conducted using the immunogenicity data collected up to 21 days after the 2nd dose and the safety data collected up to 28 days after the 2nd dose in all subjects enrolled in Part A. Additional interim analysis will be performed using the safety and immunogenicity data collected up to 26 weeks after the 2nd dose in all subjects enrolled in Part A.

CONFIDENTIAL

<table>
<tr>
<td></td>
<td>
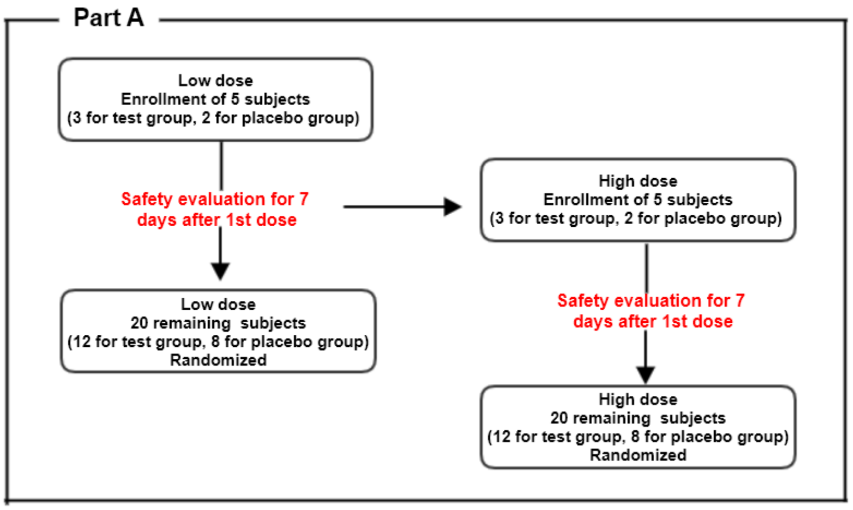

• **Part B**

Once the safety of the IP is confirmed in Part A, the study will proceed to Part B consisting of 2 dose levels of EuCorVac-19 and placebo. A total of 230 eligible subjects based on the inclusion/exclusion criteria will be randomized to the low dose group, the high dose group, and the placebo group in a ratio of 100 subjects: 100 subjects: 30 subjects.

An interim analysis will be conducted using the immunogenicity and safety data collected up to 21 days and 28 days, after the 2nd dose in all subjects in Part B. A final analysis will be performed on the immunogenicity and safety data collected up to 52 weeks after the 2nd dose in Part A and Part B.

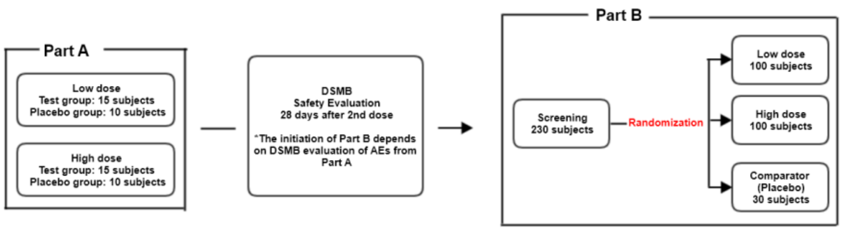

</td>
</tr>
<tr>
<td>**Investigational product**</td>
<td>
• **Test drug**: EuCorVac-19 (COVID-19 vaccine), EuBiologics Co., Ltd.
- Low dose: Antigen bulk 10 µg/vial
- High dsose: Antigen bulk 20 µg/vial
• **Comparator** (Placebo): Dai Han isotonic sodium chloride inj.
</td>
</tr>
<tr>
<td>**Dosage, route of**</td>
<td>Investigational product and comparator: Two intramuscular (IM) doses, 0.5 mL</td>
</tr>
</table>

<table>
<tr><td>administration, and method of administration</td><td>per dose, will be administered to the deltoid muscle with 3-week interval.</td></tr>
<tr><td>Sample size</td><td>Since this exploratory study is not intended to test a statistical hypothesis, sample size was not statistically calculated.

Part A
Target sample size is based on subjects who have at least one dose of the IP after randomization.
<table>
<tr><th></th><th>Low dose group</th><th>High dose group</th></tr>
<tr><td>Number of test group subjects</td><td>15 subjects</td><td>15 subjects</td></tr>
<tr><td>Number of placebo group subjects</td><td>10 subjects</td><td>10 subjects</td></tr>
<tr><td>Total number of subjects</td><td>25 subjects</td><td>25 subjects</td></tr>
</table>

Part B
Target sample size is based on subjects who have at least one dose of the IP after randomization and are available for immune response analysis.
At least 15% of overall subjects will be recruited from those between 51 and 75 years of age.
<table>
<tr><th></th><th>Low dose group</th><th>High dose group</th><th>Placebo comparator group</th></tr>
<tr><td>Number of subjects for immune response analysis</td><td>85 subjects</td><td>85 subjects</td><td>25 subjects</td></tr>
<tr><td>Total number of subjects considering 15% of drop-out rate</td><td>100 subjects</td><td>100 subjects</td><td>30 subjects</td></tr>
</table>
</td></tr>
<tr><td>Inclusion criteria</td><td>1) Individuals who voluntarily decide to participate in this study and provide written informed consent
- Healthy male and female adult at the age of 19 to 50 years (Part A)</td></tr>
</table>

	- Healthy male and female adult at the age of 19 to 75 years (Part B)<br>2) Individuals who, after receiving detailed explanations about the study, voluntarily decide to participate in this study and provide written informed consent<br>3) Individuals who are available for all visit procedures including telephone visits during the study period
**Exclusion criteria**	1) COVID-19 positive based on reverse transcription-polymerase chain reaction (RT-PCR) using upper respiratory tract (oropharyngeal, nasopharyngeal) or lower respiratory tract (sputum) sampling or COVID-19 antibody (IgM and/or IgG) positive<br>2) History of SARS-CoV, MERS-CoV or SARS-CoV-2 infection<br>3) Increased risk of exposure to SARS-CoV-2 (e.g., healthcare workers in direct contact with patients with COVID-19)<br>4) History of vaccination against SARS-CoV, MERS-CoV, or SARS-CoV-2<br>5) Immune system disorders including immunodeficiency disease<br>6) Planned blood donation or transfusion during the study period<br>7) Planned administration of other vaccines from 4 weeks before the 1st dose to 4 weeks after the 2nd dose of the IP<br>8) Height and/or weight measurements at screening:<br>① Body weight <40 kg or > 100 kg<br>② Body mass index <18 kg/m^2 or > 30 kg/m^2<br>9) Clinically significant abnormalities in clinical laboratory test, ECGs and chest X-ray during screening in the opinion of the investigator (e.g., Wolff-Parkinson-White syndrome)<br>10) Any planned surgery during the study period<br>11) Fever (≥ 37.5°C) within 3 days prior to screening or serious acute (acute fever, cough, respiratory distress, chills, myalgia, headache, sore throat, anosmia, or ageusia) or chronic infection within 7 days prior to screening requiring systemic antibiotics or antivirals<br>12) Evidence or history of serious acute, chronic, or progressive disease (e.g., cancer, diabetes mellitus, chronic pulmonary disease, acquired immune deficiency syndrome, blood dyscrasias, or immune system, urinary system, mental, musculoskeletal system, cardiovascular system, respiratory system, endocrine, nervous system, hepatobiliary system, or renal disorders, etc.) which, in the opinion of the investigator, makes the individual ineligible for

	the study<br>13) Positive serum tests during screening [type B hepatitis, human immunodeficiency virus (HIV), type C hepatitis]<br>14) History of treatment with antipsychotics or opioid analgesic dependence within 6 months prior to IP dosing<br>15) History of severe allergic reactions (e.g., anaphylaxis, Guillain-Barré Syndrome) or severe hypersensitivity reactions to the IP or any of its components<br>16) History of therapy that might affect immunity: treatment with immunosuppressants or immune modifying drugs, anticancer therapy, or radiotherapy within 3 months prior to screening<br>17) History of systemic steroids (prednisone ≥10mg/day for > 14 consecutive days) within 3 months prior to screening. Topical, inhaled, and intranasal corticosteroids are allowed regardless of dose.<br>18) Past treatment within 3 months prior to screening, or planned treatment during the study period, with immunoglobulin or blood derivatives<br>19) Individual with thrombocytopenia or other coagulation disorders for whom intramuscular (IM) injections are contraindicated or individual who is on anticoagulant therapy*<br>* Anticoagulant therapy: continuous use of anticoagulants such as coumarin/warfarin or new oral anticoagulants/antiplatelets<br>20) History of excessive alcohol consumption or drug addiction<br>21) Women of childbearing potential who do not agree to use medically allowed methods of contraception* or to be heterosexually inactive until 60 days after the last dose of the IP<br>* Hormonal contraceptive, intrauterine device(IUD(intrauterine device) or IUS(intrauterine system)), tubal ligation double-blocking method(condom for male or female), cervical cap or diaphragm, complex method such as contraceptive sponge), single-blocking method using spermicides<br>22) Pregnant or breastfeeding woman<br>23) Treatment with other IPs within 6 months prior to participation in this study<br>24) The investigator who is directly related to this study or sub-investigator/study coordinator who is supervised by investigator or their family member<br>25) Other reasons including medical reasons based on which the individual is

	considered to be ineligible for this study in the opinion of the investigator
**Safety assessment**	• Adverse events (AEs)<br>① Immediate AEs occurring within 30 minutes after each IP dosing<br>② Solicited local and systemic AEs occurring for 7 days(Day 0~Day 6) after each IP dosing<br>✓ Local AEs : pain, tenderness, erythema/redness, induration/swelling, itchiness<br>✓ Systemic AEs : fever, fatigue/malaise, chills/rigors, headache, myalgia, joint pain, diarrhea, vomiting, abdominal pain, mucocutaneous reaction/rash, cough, acute bronchospasm, respiratory distress<br>③ Unsolicited AEs occurring within 28 days after the last IP dosing<br>④ Serious AEs (SAEs) occurring within 52 weeks after the last IP dosing<br>⑤ Adverse events of special interest (AESIs) occurring within 52 weeks after the last IP dosing<br>• Clinical laboratory tests (hematology/blood chemistry, urinalysis)<br>• Vital signs<br>• Physical examination
**Immunogenicity assessment**	**<Part A>**<br>① Geometric mean titer (GMT) and geometric mean fold rise (GMFR) compared to the baseline(Visit 2) of antigen protein-specific antibodies as measured by enzyme-linked immunosorbent assay (ELISA) at pre-dose (Visit 2), on Day 21 (Visit 5) after the 1st dose, and on Day 21 (Visit 8), Week 26 (Visit 10), and Week 52 (Visit 11) after the 2nd dose of the IP<br>② Proportion of subjects with seroconversion to antigen protein-specific antibodies as measured by ELISA at pre-dose(Visit 2), on Day 21 (Visit 5) after the 1st dose and on Day 21 (Visit 8), Week 26 (Visit 10), and Week 52 (Visit 11) after the 2nd dose of the IP (seroconversion rate [SCR])†<br>†Seroconversion rate is defined as a ≥4-fold increase in GMT post-dose of the IP from pre-dose(Visit 2).<br>③ GMT and GMFR compared to the baseline(Visit 2) of neutralizing antibodies to SARS-CoV-2 as analyzed by Focus Reduction Neutralization Test (Wild-type SARS-CoV-2) at pre-dose (Visit 2), on Day 21 (Visit 5) after the 1st dose, and on Day 21 (Visit 8), Week 26 (Visit 10), and Week 52 (Visit 11) after the 2nd dose of the IP<br>④ Proportion of subjects with seroconversion to neutralizing antibodies to

	SARS-CoV-2 as analyzed by Focus Reduction Neutralization Test (Wild-type SARS-CoV-2) at pre-dose(Visit 2), on Day 21 (Visit 5) after the 1st dose and on Day 21 (Visit 8), Week 26 (Visit 10), and Week 52 (Visit 11) after the 2nd dose of the IP (seroconversion rate [SCR]) ‡<br>‡Seroconversion rate is defined as a ≥4-fold increase in GMT post-dose of the IP from pre-dose(Visit 2).<br><br>**<Part B>**<br>① GMT and GMFR compared to the baseline(Visit 2) of antigen protein-specific antibodies as measured by ELISA at pre-dose (Visit 2), on Day 21 (Visit 5) after the 1st dose, and on Day 21 (Visit 8), Week 26 (Visit 10), and Week 52 (Visit 11) after the 2nd dose of the IP<br>② Proportion of subjects with seroconversion to antigen protein-specific antibodies as measured by ELISA on Day 21 (Visit 5) after the 1st dose and on Day 21 (Visit 8), Week 26 (Visit 10), and Week 52 (Visit 11) after the 2nd dose of the IP (SCR)†<br>†Seroconversion rate is defined as a ≥4-fold increase in GMT post-dose of the IP from pre-dose(Visit 2).<br>③ GMT and GMFR compared to the baseline(Visit 2) of neutralizing antibodies to SARS-CoV-2 as analyzed by Focus Reduction Neutralization Test (Wild-type SARS-CoV-2) at pre-dose (Visit 2), on Day 21 (Visit 5) after the 1st dose, and on Day 21 (Visit 8), Week 26 (Visit 10), and Week 52 (Visit 11) after the 2nd dose of the IP<br>④ Proportion of subjects with seroconversion to neutralizing antibodies to SARS-CoV-2 as analyzed by Focus Reduction Neutralization Test (Wild-type SARS-CoV-2) at pre-dose(Visit 2), on Day 21 (Visit 5) after the 1st dose and on Day 21 (Visit 8), Week 26 (Visit 10), and Week 52 (Visit 11) after the 2nd dose of the IP (seroconversion rate [SCR])‡<br>‡Seroconversion rate is defined as a ≥4-fold increase in GMT post-dose of the IP from pre-dose(Visit 2).
**Exploratory assessment**	**<Part A & Part B>**<br>T cell immune response markers as analyzed by Ex vivo IFNγ and IL-4 ELISpot at pre-dose (Visit 2), on Day 21(Visit 5) after the 1st dose and on Day 21 (Visit 8) after the 2nd dose of the IP

	* Exploratory assessment will be done on all subjects in Part A and 45 subjects in Part B

CONFIDENTIAL

▣ Schedule of Assessments (Part A & B)

**Period**	**Screening**	**Baseline/ 1st dose**	**Main study**						**End of main study visit**[11]	**Follow-up interim visit**	**End of follow-up visit**[11]
			**Telephone visit**	**Interim visit**	**2nd dose**	**Telephone visit**	**Interim visit**	**Interim visit**			
**Visit**	**V1**	**V2**	**V3**	**V4**	**V5**	**V6**	**V7**	**V8**	**V9**	**V10**	**V11**
**Schedule**	Day -7 ~ -1	Day 0	Day 2~5	Day 7	Day 21	Day 2~5 after the 2nd dose	Day 7 after the 2nd dose	Day 21 after the 2nd dose	Day 28 after the 2nd dose	Week 26 after the 2nd dose	Week 52 after the 2nd dose
**Visit window**	-	-	-	+ 3 D	+ 5 D	-	+ 3 D	+ 5 D	+ 7 D	± 2 W	± 4 W
Informed consent[1]	X										
Demographics and baseline information	X										
Medical history/surgical history	X	X									
Prior/concomitant medication	X	X	X	X	X	X	X	X	X	X[12]	X[12]
Vital signs[2]	X	X		X	X		X	X	X	X	X
Physical examination	X	X		X	X		X	X	X		
Weight and height	X										
ECG and chest X-ray[3]	X										
COVID-19 diagnostic test and antibody test[4]	20 µL	20 µL									
Clinical laboratory tests[5]	10 mL			10 mL			10 mL		10 mL		
Pregnancy test[6]	X				X				X		X

**Period**		**Screening**	**Baseline/ 1st dose**	**Main study**						**End of main study visit [11)]**	**Follow-up interim visit**	**End of follow-up visit[11)]**
				**Telephone visit**	**Interim visit**	**2nd dose**	**Telephone visit**	**Interim visit**	**Interim visit**			
**Visit**		**V1**	**V2**	**V3**	**V4**	**V5**	**V6**	**V7**	**V8**	**V9**	**V10**	**V11**
**Schedule**		Day -7 ~ -1	Day 0	Day 2~5	Day 7	Day 21	Day 2~5 after the 2nd dose	Day 7 after the 2nd dose	Day 21 after the 2nd dose	Day 28 after the 2nd dose	Week 26 after the 2nd dose	Week 52 after the 2nd dose
**Visit window**		-	-	-	+ 3 D	+ 5 D	-	+ 3 D	+ 5 D	+ 7 D	± 2 W	± 4 W
Inclusion/exclusion criteria checking		X	X									
Randomization			X									
Telephone monitoring[7)]				X			X					
Immunogenicity blood sampling[8)]	ELISA, FRNT test		8 mL			5 mL			5 mL		5 mL	5 mL
	T cell immune response test		20 mL			20 mL			20 mL			
IP dosing			X			X						
AE checking			X[9)]	X	X	X[9)]	X	X	X	X	X[13)]	X[13)]
Subject diary dispensing[10)]			X		X	X		X	X			
Subject diary retrieving					X	X		X	X	X		

1) Written informed consent may be obtained prior to screening visit procedures. For those who meet all of the inclusion/exclusion criteria at screening, Visits 1 and 2 can simultaneously be conducted with overlapping procedures done only once. In this case, baseline test results will be replaced with screening test results for the purpose of analysis.

2) Blood pressure, pulse, respiratory rate, and tympanic temperature will be measured. If possible, vital signs will be measured prior to any planned tests. Measurements will be taken in a sitting position after 5 minutes of resting.
3) Posterior-anterior (PA) test will be performed for chest X-ray. During the screening period, one re-test may be performed at the discretion of the investigator.
4) At Visit 1 (screening), both RT-PCR using upper respiratory tract sampling (oropharyngeal/ nasopharyngeal swab), and an additional lower respiratory tract sampling (sputum) in the presence of symptoms such as cough and sputum, and antibody test (IgG/IgM; 20 µL of blood) will be performed. At Visit 2, RT-PCR or antibody test will be performed. Antibody test will be performed at institution at Visit 1 (screening), and kit for COVID-19 diagnostic test will be provided by institution or sponsor to perform antibody test at Visit 2. Subjects who have the symptoms of COVID-19 infection (fever or respiratory symptoms) after randomization should inform investigator of their symptoms as followed by COVID-19 guidelines of Central Disease Control Headquarters·Central Disaster Management Headquarters, and will be instructed to visit study institution to be treated, observed and assessed.
5) Subject should keep fasting(prohibition of food or beverage except water for 8 hours) if possible before blood samples collection for clinical laboratory tests, and the following clinical laboratory tests will be done. For screening tests, test results obtained within 4 weeks prior to the screening visit may be used instead, if available. During the screening period, one re-test may be performed at the discretion of the investigator. Virus and blood coagulation tests will be performed only at Visit 1.
   - Hematology: RBC, Hemoglobin, Hematocrit, Platelets count, WBC, WBC Differential count (neutrophil, lymphocyte, monocyte, eosinophil, basophil)
   - Blood chemistry: Glucose, BUN, Creatinine, Uric acid, Total protein, Total bilirubin, Albumin, ALP, ALT, AST, GGT (γ-GT), Total Cholesterol, Ca, Na, P, Cl, K
   - Urinalysis: Protein (Albumin), Glucose, WBC, Ketone, Blood (RBC)
   - Blood coagulation: PT, aPTT (at Visit 1 only)
   - Virus test: HBsAg, HIV Ag/Ab, anti-HCV (at Visit 1 only)
6) All women of childbearing potential will be tested for pregnancy by urine or serum (approximately 5 mL of blood) testing. Women of childbearing potential are defined as those who had menarche and are not surgically sterile (hysterectomy, bilateral tubal ligation, or bilateral ovariectomy) or postmenopausal. Menopause is defined as having no menses for ≥12 months from the final menstrual period without an alternative cause. If necessary, an additional pregnancy test can be done at other visits unscheduled for the test. A subject with a positive pregnancy outcome will be withdrawn from the study.
7) A trained sub-investigator will call the subjects to instruct them to collect AEs in their subject diaries and to schedule their next visits.
8) Subject has to keep fasting(Prohibition of food or beverage except water for 8 hours) before blood samples collection for immunogenicity analysis. Blood samples for immunogenicity analysis will be collected prior to IP dosing. Immunogenicity will collectively be tested by the central laboratory.
9) The subject will be monitored for at least 30 minutes post-dose for immediate AEs (anaphylaxis associated).
10) Subject diaries will be completed as follows. Subject diary which was distributed at Visit 7 will be collected at Visit 8 to identify occurred Adverse Events and then re-distribute (But, subject diary will be newly distributed once remaining column in diary is insufficient.)
    ① IP dosing day (Visit 2, Visit 5) ~ Day 7 after dosing (Visit 4, Visit 7): solicited local/systemic AEs and unsolicited AEs
    ② Day 7 after IP dosing (Visit 4, Visit 7) ~ Day 28 after IP dosing (Visit 7, Visit 9): unsolicited AEs

11) If possible, subjects who are withdrawn from the study will visit the institution for required procedures. Those who are withdrawn prior to Visit 9 (end of main study visit) will perform Visit 9 procedures. Those who are withdrawn after Visit 9 will perform Visit 11 procedures (end of follow-up visit) except for blood sampling for immunogenicity testing. If consent is obtained from the subject, telephone visits will be conducted by a trained sub-investigator who will call the subject at the time points of Visit 10 (Week 26 after the 2nd dose of the IP) and Visit 11 (Week 52 after the 2nd dose of the IP) for serious ADRs and AESIs. Subject who has not performed administration of 2nd dose investigational product will conduct Visit 10 and Visit 11 on the basis of scheduled date of 2nd dose administration.
12) Concomitant drugs administered for SAEs and AESIs will be collected.
13) Status of SAEs and AESIs will be checked. In the event of an AESI, additional tests may be conducted as necessary at the discretion of the investigator, and if necessary, the DSMB may meet to review the case for status of ADR and enhanced disease.

1. Introduction

1.1 Background of study

Severe acute respiratory syndrome coronavirus 2 (SARS-CoV-2) is the pathogen of Coronavirus Disease-19 (COVID-19) that may result in acute respiratory distress syndrome in children and adults. SARS-CoV-2 is associated with a variety of signs and symptoms ranging from asymptomatic to mild symptoms such as fever, chills, cough, respiratory distress, fatigue, myalgia, headache, ageusia, anosmia, pharyngolaryngitis, nausea, vomiting, and diarrhea to severe diseases such as pneumonia and sepsis. Starting in China in December 2019, the novel virus has been fast spreading across the globe, within 3 months resulting in 113,702 infected cases and around 4,000 deaths in 114 countries as of 10 Mar 2020, which led to the declaration of COVID-19 pandemic by the World Health Organization as of 11 Mar 2020. Vaccines are considered to be the most effective tool to end COVID-19 by vaccination-induced herd immunity. Efforts to develop COVID-19 vaccine candidates by many developers and organizations are ongoing.

Developed by EuBiologics Co., Ltd. to prevent COVID-19, EuCorVac-19 is a recombinant protein vaccine in which a receptor-binding domain (RBD) antigen is bound to liposomes consisting of E. coli produced Monophosphoryl Lipid A (EcML), Cobalt-Porphyrin-Phospholipid (CoPoP), Dipalmitoylphosphatidylcholine (DOPC) and cholesterol.

EuCorVac-19 was found to have sufficient immunogenicity and safety in animal studies including efficacy, safety pharmacology, toxicity, genetic toxicity, and distribution studies. In the efficacy study with EuCorVac-19, titers of both anti-RBD Immunoglobulin G (IgG) and neutralizing antibodies increased more than 10 folds after the 2nd dose in comparison with the 1st dose, indicating an increase in efficacy in proportion to dose. Results from the analysis of cellular immunity 2 weeks after the 2nd dose showed that IFNγ, TNF-α, IL-2, and IL-4 levels tended to increase in proportion to dose, with a significant rise observed particularly in high-dose groups. Given Th1 biased immune response, antibody-dependent adverse events (AEs) are unlikely in humans.

Results from the safety pharmacology study also demonstrated that there were no impacts of EuCorVac-19 on respiratory system, central nervous system, and cardiovascular system. In single-dose toxicity and 2-weeks repeat-dose toxicity studies to evaluate the potential toxicity of EuCorVac-19 in rodents, none of the animals died due to EuCorVac-19 and there were no toxicological findings of EuCorVac-19 in general symptoms, weight change, body temperature,

and macroscopic necropsies.

Based on these non-clinical study results, a Phase 1/2 clinical trial is planned to be conducted to evaluate the safety and immunogenicity of EuCorVac-19 in healthy adults.

Although no vaccines have yet been approved in South Korea for the prevention of COVID-19, a number of research institutes across the world including vaccine developers are currently developing COVID-19 vaccine candidates using different technologies and platforms including deoxyribonucleic acid (DNA), ribonucleic acid (RNA), virus, viral vector, and protein vaccines. Recently, the United Kingdome granted world's first emergency-use authorization to Pfizer's COVID-19 RNA vaccine. Phase 3 clinical trials are ongoing for the RNA vaccines of Pfizer and Moderna. Additionally, Phase 3 trials are also being conducted for AstraZeneca's inactivated virus vaccine using adenovirus vectors as well as for Novavax's recombinant protein vaccine using the same platform technology as EuCorVac-19.

1.2 Study rationale

1.2.1 Rationale for study design

A number of research institutes across the world including vaccine developers are currently developing COVID-19 vaccine candidates using different technologies and platforms including DNA, RNA, virus, viral vector, and protein vaccines. EuBiologics Co.'s EuCorVac-19 is a recombinant protein vaccine against COVID-19 in which protein is as an antigen. As with live vaccines and killed vaccines, recombinant protein vaccines have been used for decades, proving their efficacy and safety in humans. While the safety of EuCorVac-19 is expected to be excellent in that its antigen is made of protein, rather than genetic materials, protein antigen itself might not be able to ensure sufficient potency unlike live vaccines or killed vaccines. To overcome this issue, immune boosters were added to the protein antigen thereby enhancing the potency of the vaccine. EcML, a TLR4 agonist monophosphoryl lipid A that facilitates humoral and cellular immunity, is used as an immune booster in the form of a liposome; to enhance the water solubility of lipophilic EcML, EcML is inserted between the double membranes of the liposome. Besides EcML, CoPoP is newly added to EuCorVac-19 for further improvement in antibody production as CoPoP facilitates antigen presentation and particularization on the surface of the liposome. Along with EcML, CoPoP is inserted between the double membranes of the liposome to which the histidine-tag antigen protein is bound, resulting in the antigen displayed on the surface of the liposome. Antigen particularization on the liposomal surface enables more efficient antigen presenting in

comparison with simply mixing antigens, ultimately enhancing the production of binding antibodies and neutralizing antibodies.

Target antigens for the COVID-19 vaccine are usually spike proteins of SARS-CoV-2. Among the spike proteins, RBD, a virus domain that directly binds to human angiotensin-converting enzyme 2 (ACE2) for infection, was selected by EuBiologics Co., Ltd. as an antigen of EuCorVac-19.

1.2.2 Rationale for dose selection

EuBiologics' COVID-19 recombinant protein vaccine, EuCorVac-19, has been developed to prevent COVID-19 in adults aged 19 to 75. The amount of the active ingredient of EuCorVac-19 is 10μg or 20μg per dose (0.5mL); the optimal dose will be selected via clinical trials. Phase 3 clinical trial will be conducted in adults and children after the results of this Phase 1/2 study are secured.

As for the immune boosters of this vaccine, 10 μg or 20 μg of EcML and 20 μg or 40 μg of CoPoP are to be included in proportion to the amount of the antigen. Results from a study on neutralizing antibody titers after RBD antigen particularization by CoPoP, an immunogenicity and challenge study in hACE-2 mice, and an animal immunogenicity study in mice showed that a low concentration of EuCorVac-19 (0.1 μg/dose or 0.2 μg/dose) was still effective in terms of IgG enzyme-linked immunosorbent assay (ELISA) antibody titer, neutralizing antibody titer, cellular immune response, and protective effect, with a significant dose-dependent increase in responses. Of particular note was 5 μg/dose that demonstrated complete protection in the animal challenge model. Details of the formulation of the investigational product (IP) by component and rationale for dose selection are described in the Investigator's Brochure[6)].

1.2.3 Potential risk-benefit assessment

This study is planned to evaluate the safety and immunogenicity of EuCorVac-19 as an IP in healthy adults. As this is a first-in-human trial, the IP has never been used in humans. Major adverse drug reactions (ADRs) collected from clinical trials in adults treated with COVID-19 vaccine candidates in development by various companies including Novavax, AstraZeneca, Moderna, CanSino, and Pfizer are pain, tenderness, fatigue, headache, and fever. [(7-11)]

Based on the results of non-clinical studies, two doses of EuCorVac-19 given in 3-week interval to healthy adults are unlikely to cause serious safety concerns such as serious AEs (SAEs). Given that the safety and efficacy of the IP in humans cannot be established based on non-clinical studies, the safety and efficacy of EuCorVac-19 will be evaluated in this study. Solicited AEs after IP dosing including potential risk are anticipated to be manageable by standard of care at the discretion of the investigator.

2. Study objectives

2.1 Part A

1) Primary Objective
To evaluate the safety and tolerability of EuCorVac-19 at all dose levels in healthy adults aged 19 to 50.

2) Secondary Objective
To evaluate the immune response of EuCorVac-19 at all dose levels in healthy adults aged 19 to 50.

2.2 Part B

1) Primary Objective
To evaluate the immune response of EuCorVac-19 against SARS-COV-2 in healthy adults aged 19 to 75.

2) Secondary Objective
To evaluate safety and tolerability of EuCorVac-19 against SARS-CoV-2 in healthy adults aged 19 to 75.

3. Overall study design and plans

3.1 Study design

This study consists of two parts: Part A to evaluate the safety and tolerability of EuCorVac-19 in healthy adults and Part B to evaluate the safety, tolerability, and immune response of EuCorVac-19 in healthy adults.

Amongst individuals who voluntarily provide written informed consent to participation in this study, those eligible based on the inclusion/exclusion criteria will be randomized to a dose level to receive two doses of the IP with 21 days apart and be assessed for safety, tolerability, and immunogenicity according to the following schedules; the study schedules for both Part A and B are same.

<table>
<tr><th>Visit 1</th><th>Visit 2</th><th>Visit 3</th><th>Visit 4</th><th>Visit 5</th><th>Visit 6</th><th>Visit 7</th><th>Visit 8</th><th>Visit 9</th><th>Visit 10</th><th>Visit 11</th></tr>
<tr><td>Day -7~-1</td><td>Day 0</td><td>Day 2~5</td><td>Day 7</td><td>Day 21</td><td>Day 2~5 after the 2nd dose</td><td>Day 7 after the 2nd dose</td><td>Day 21 after the 2nd dose</td><td>Day 28 after the 2nd dose</td><td>Week 26 after the 2nd dose</td><td>Week 52 after the 2nd dose</td></tr>
<tr><td>-</td><td>-</td><td>-</td><td>+3D</td><td>+5D</td><td>-</td><td>+3D</td><td>+5D</td><td>+7D</td><td>±2W</td><td>±4W</td></tr>
<tr><td rowspan="3">Screening</td><td rowspan="2">Randomization 1st dose</td><td colspan="6">Main study</td><td rowspan="2">End of main study visit</td><td rowspan="2">Follow-up interim visit</td><td rowspan="2">End of follow-up visit</td></tr>
<tr><td>Telephone visit</td><td>Interim visit</td><td>2nd dose</td><td>Telephone visit</td><td>Interim visit</td><td>Interim visit</td></tr>
<tr><td>Safety and immunogenicity assessment</td><td colspan="2">Safety assessment</td><td>Safety and immunogenicity assessment</td><td colspan="2">Safety assessment</td><td>Safety and immunogenicity assessment</td><td>Safety assessment</td><td colspan="2">Safety and immunogenicity assessment</td></tr>
</table>

3.1.1 Part A

Part A consists of two dose levels(low dose, high dose) of EuCorVac-19. Each dose group consists of 15 subjects in test group and 10 subjects in placebo group.

The first 5 subjects will be randomized and enrolled to the low dose group (Sentinel group: 3 subjects in Test group, 2 subjects in Placebo group); once the safety of the IP is confirmed in these subjects over 7 days after the 1st dose of the IP, the study will proceed to enroll the rest of 20 subjects to the low dose group. The rest of 20 subjects will be randomized to 12 subjects in Test group and 8 subjects in Placebo group. Also, the first 5 subjects will be randomized and enrolled

to the high dose group (Sentinel group: 3 subjects in Test group, 2 subjects in Placebo group); If no Grade ≥3 adverse drug reactions (ADRs) are reported within 7 days (Day 0 ~ Day 6) after the 1st dose of the IP, the rest of 20 subjects to the high dose group will be enrolled. The 20 subjects in the high dose group will be randomized to 12 subjects in Test group and 8 subjects in Placebo group.

If Grade ≥3 ADRs are reported within 7 days after the 1st dose of the IP in any of the first 5 subjects(Sentinel group) in each dose groups, the Data and Safety Monitoring Board (DSMB) will review the safety of the IP and determine whether to continue the study. If applicable, subject enrollment will be stopped and additional dosing in the existing subjects suspended until the decision is made on study continuation.

Once the safety results up to 28 days after the 2nd dose are collected from all subjects in Part A, the DSMB will review the safety data from all subjects enrolled in the high and low dose groups in Part A. The DSMB will assess if the event of ADRs in all subjects meet the criteria for study suspension and figure out the relationship between ADR and IP, based on which proceeding to Part B will be decided.

An interim analysis will be conducted using the immunogenicity data collected up to 21 days after the 2nd dose and the safety data collected up to 28 days after the 2nd dose in all subjects enrolled in Part A. Additional interim analysis will be performed using the safety and immunogenicity data collected up to 26 weeks after the 2nd dose in all subjects enrolled in Part A.

3.1.2 Part B

Once the safety of the IP is confirmed in Part A, the study will proceed to Part B consisting of 2 dose levels of EuCorVac-19 and placebo. A total of 230 eligible subjects based on the inclusion/exclusion criteria will be randomized to the low dose group, the high dose group, and the placebo group in a ratio of 100 subjects: 100 subjects: 30 subjects.

An interim analysis will be conducted using the immunogenicity and safety data collected up to 21 days and 28 days, after the 2nd dose in all subjects in Part B. A final analysis will be performed on the immunogenicity and safety data collected up to 52 weeks after the 2nd dose in Part A and Part B.

3.2 Participating institutions

This study will be conducted at institutions located in Republic of Korea. The full list of study institutions is provided in Attachment 3. Study institutions and the sponsor's organizations.

3.3 Assigning to treatment groups and assigning procedures

This study will use Interactive Web Response System (IWRS) for randomization, and pharmacist will identify the random number of subject via IWRS to release appropriate IP.

3.3.1 Part A

Individuals who provide written informed consent to participation in Part A will be given a screening number in the order of written informed consent. The screening number will consist of [2-digit number representing the study institution]-SA-[3-digit serial number]. Afterwards, those who meet the inclusion/exclusion criteria will be randomized to each dose group in the order of enrollment and given a unique randome number consisting of [2-digit number representing the study institution]-RA[1-digit number representing the dose group*]-[3-digit serial number].(*Dose group number '1': low dose sentinel group, '2': low dose group, '3': high dose sentinel group, '4': high dose group)

The first 5 subjects in each dose group for safety evaluation will be randomized to Test group and Placebo group in 3:2 ratio; once the safety is confirmed in the first 5 subjects in low dose group, the first 5 subjects in high dose group and the rest 20 subjects in low dose group will proceed to enroll. After the safety is confirmed in the first 5 subjects in high dose group, the rest 20 subjects in high dose group will proceed to enroll. The rest 20 subjects in each dose group will also be randomized to Test group and Placebo comparator group in 3:2 ratio.

3.3.2 Part B

Subjects will be assigned to each treatment group in a ratio of 10:10:3 (low dose group: high dose group: placebo comparator group) and stratified by age (19 to 50 years of age versus 51 to 75 years of age). The randomization manager will randomly choose a block size amongst the multiples of the number of treatment groups and generate randomization tables using the SAS

(version 9.4 or above) program.

Individuals who provide written informed consent to participation in Part B will be given a screening number in the order of written informed consent. The screening number will consist of [2-digit number representing the study institution]-SB-[3-digit serial number]. Afterwards, those who meet the inclusion/exclusion criteria will be randomized by central randomization plans in which subjects are stratified by age. In the order of enrollment as determined by the investigator, the subjects will be given a unique randomization number consisting of [2-digit number representing the study institution]-RB[1-digit stratification code*]-[3-digit serial number].
(*stratification code ‘1’: 19 to 50 years of age, ‘2’: 51 to 75 years of age)

4. Study assessment methods and procedures

4.1 Assessment methods

4.1.1 Safety assessment

In the safety assessment tests, clinically significant abnormalities identified prior to the 1st dose of the IP (Visit 2) will be collected as medical history while clinically significant abnormalities identified after IP dosing will be collected as AEs provided that they meet the definition of AEs.

4.1.1.1 Adverse events

Information on AEs will be collected via interviews and spontaneous reports during regular and/or additional visits, and subjects will be instructed to complete subject diaries without missing data. For definition, collection, recording, assessment, and reporting procedures for AEs, see Section 8 'Adverse events' in this protocol.

As immediate AEs may occur after IP dosing, subjects will be monitored for 30 minutes post-dose for immediate AEs (anaphylaxis associated). The study institution should ensure that drugs and medical equipment are readily available as appropriate for the identification and treatment of immediate AEs. To ensure that immediate AEs can be identified and treated, the investigator or a designated person should be present during the overall dosing procedures and post-dose monitoring.

4.1.1.2 Clinical laboratory tests

To evaluate overall health status, clinical laboratory tests will be performed on all subjects by a laboratory at the institution according to the Schedule of Assessment. Collection, management, and handling of samples will be performed in compliance with the laboratory-specific standard operating procedures (SOPs).

Besides the tests specified in the Schedule of Assessment, additional tests may be performed if deemed necessary by the investigator.

For the visits in which blood sampling is scheduled for clinical laboratory tests, the subject should fast, if possible, before the visit (except for water, any beverage or food will be prohibited within

8 hours before testing). For screening (Visit 1) tests, test results obtained within 4 weeks prior to the screening visit may be used instead, if available. During the screening period, one re-test may be performed at the discretion of the investigator. If a re-test is done, the re-test result should be used for the final assessment of the inclusion/exclusion criteria. Virus and blood coagulation tests will be performed only at Visit 1.

- Hematology: RBC, Hemoglobin, Hematocrit, Platelets count, WBC, WBC Differential count (neutrophil, lymphocyte, monocyte, eosinophil, basophil)
- Blood chemistry: Glucose, BUN, Creatinine, Uric acid, Total protein, Total bilirubin, Albumin, ALP, ALT, AST, GGT (γ-GT), Total Cholesterol, Ca, Na, P, Cl, K
- Urinalysis: Protein (Albumin), Glucose, WBC, Ketone, Blood (RBC)
- Blood coagulation: PT, aPTT
- Virus test: HBsAg, HIV Ag/Ab, anti-HCV

4.1.1.3 Vital signs

For vital signs, blood pressure, pulse, respiratory rate, and tympanic temperature will be measured. If possible, vital signs will be measured prior to any planned tests according to the Schedule of Assessment. Measurements will be taken in a sitting position after 5 minutes of resting.

4.1.1.4 Physical examination

To assess health status and AEs, physical examination will be performed according to the Schedule of Assessment on each subject by means of visual inspection, palpation, percussion, and auscultation.

Physical examination includes examination of appearance, skin, head/neck, chest/lungs, heart, abdomen, urinary/reproductive system, limbs, musculoskeletal system, nervous system, and lymph nodes.

4.1.2 Immunogenicity assessment

Subject has to keep fasting (prohibition of food or beverage except water for 8 hours) before blood

sample collection for immunogenicity assessment. At least 28mL at pre-dose (Visit 2), at least 25mL on Day 21 after the 1st dose (Visit 5), at least 25mL on Day 21 after the 2nd dose (Visit 8), and at least 5mL on Week 26 (Visit 10), and Week 52 (Visit 11) after the 2nd dose of blood samples will be collected. Each of 20mL of blood samples collected at Visit 2, Visit 5 and Visit 8 will be stored in appropriate container and at appropriate temperature to be sent to the central laboratory (Vaccine/Bio Research Institute at the Catholic University of Korea, College of Medicine).
Besides, blood samples collected at other visits will be stored at room temperature for at least 30 minutes to 2 hours for blood coagulation reaction. After the coagulation, blood will be separated to achieve serum by a centrifuge. Separated serum will be split into several and appropriate containers and stored at appropriate temperature before sending to the central laboratory (International Vaccine Institute).

Humoral immunity will be tested by the International Vaccine Institute, and cellular immunity will be tested by the Vaccine/Bio Research Institute at the Catholic University of Korea, College of Medicine. Samples for immunogenicity testing will be sent to the central laboratory designated for each test for collective measurement and analysis.

- **Humoral immunity testing (total volume of blood to be collected: at least 8 mL pre-dose (Visit 2), at least 5 mL at other visits)**

1) Antigen protein-specific antibody test using ELISA: approximately 3 mL of blood
   - To assess the titer of the antibody specifically binding to the RBD of the spike protein induced by the IP in the serum of the subject treated with the IP.
   - After coating the recombinant protein containing antigen protein receptor binding domain(RBD) in 96-well plate for ELISA, each well will be washed with washing solution and then filled with blocking buffer and stored at room temperature. Then blocking buffer will be removed, each well will be washed with washing solution once, and serum of each study subjects will undergo serial dilution. Blocking buffer will be removed from the plate, washed with washing solution, and then HRP conjugated anti human IgG will be added to each well and the plate will be washed. TMB substrate will be added, and TMB stop solution will be added depends on color development, and absorbance will be measured.
   - Geometric mean titer (GMT) and geometric mean fold rise (GMFR) will be calculated for antigen protein-specific IgG on the relevant day versus pre-dose of the IP. A serum is determined positive when mean absorbance in the serum of the same dilution rate on

the relevant day is greater than “mean absorbance +3x standard deviation (SD)” in the pre-dose serum; antibody titer is defined as the reciprocal number of the value with the greatest dilution rate.

- Seroconversion is defined as a ≥4-fold increase in GMT post-dose of the IP from pre-dose, which is ≥4-fold of GMFR.

2) Neutralizing antibody test using FRNT (Wild-type SARS-CoV-2): approximately 2 mL of blood
   - To measure the amount of SARS-CoV-2-specific neutralizing antibodies induced by the vaccine, a focus reduction neutralization test (FRNT) will be performed using wild-type SARS-CoV-2.
   - Vero cells will be cultured to 96 well plate by seeding, and serum of study subjects which was diluted consecutively from 1/20 to 2-fold will be mixed with SARS-CoV-2 and induced reaction. Reacted SARS-CoV-2-serum mixture will be cultured by seeding into vero cell, then cell culture media will be removed from mixture, and cells will be fixed with formalin and antibody in cells will be stained with methanol. The fixed cells will be treated with SARS-CoV-2 nucleocapsid protein (NP) specific antibody, and the amount of foci which are created by color development will be counted by CTL reader.
   - Once diluted serum is treated, the amount of focus which is produced when only SARS-CoV-2 is treated will be counted and dilution concentration of half of this focus amount will be calculated as FRNT50 (If the amount of focus at the initial serum dilution concentration of 1/20 is not below 50% compared to the amount of focus at cells treated only with SARS-CoV-2, FRNT50 is defined as 10.) Responder decision is defined as ≥4-fold increase in FRNT50 post-dose of the IP from pre-dose.

- **Cellular immunity testing (total volume of blood to be collected: at least 20 mL)**

Ex vivo IFNγ and IL-4Enzyme-Linked Immunospot (ELISpot) T cell immune response marker test:

- Cellular response will be assessed in the vaccine treatment groups by analyzing the vaccine antigen-specific T cell count in peripheral blood mononuclear cells (PBMCs) (without proliferation by additional culture) in the blood of the participants treated with SARS-CoV-2 spike RBD vaccine antigens (low dose group, high dose group, placebo group).
- After separation of PBMC from blood samples of study subjects (treated with

heparin), 2.5 X 105 cell will be split into each well of 96 well plate which is coated with anti-human IFNγ or anti IL-4 monoclonal antibody, and then SARS-CoV-2 S1 peptide pool (Total 166 peptide) will be treated in RPMI(100units/mL penicillin, 1 mg/mL streptomycin, 10% heat inactivated FC). Then, detection monoclonal antibody and streptavidin alkaline phosphatase will be cultured and treated with BCIP/NBT substrate, and expression will be measured. Spot forming cells (SFC)/106 cell will be counted in each samples by ELISpot counter.

- Assay controls
  - Negative control: PBMC of identical study subjects which is not treated with SARS-CoV-2 S1 peptide pool
  - Positive control: anti-CD3 monoclonal antibody (Mabtech, Stockholm, Sweden) group
- Cellular response is determined “positive” when the mean value of each sample is at least 5 SFC/10^5 cell or when the post-dose value is increased at least 2-fold from the pre-dose value. Cellular response outcome will later be compared with specific Ab titer or neutralization Ab titer.

Cellular immunity testing will be done on all subjects in Part A, and only 45 subjects who signed separated Human-Derived Material ICF in Part B.

Except for some samples that might be needed in the future for re-analysis, the remaining unused samples will be collected and destroyed upon the end of study according to applicable procedures defined by EuBiologics Co., Ltd. All data related to samples obtained from the study subjects will be identified with subject identification number and remain anonymized throughout the study period. For the samples for cellular immunity testing, additional testing with remnants can be performed at the Vaccine/Bio Research Institute at the Catholic University of Korea, College of Medicine, once separate Human-Derived Material ICF is collected.

4.1.3 Others

4.1.3.1 Baseline information

Prior to entry into the study, details of the study objectives and contents will be explained to, and written informed consent will be obtained from the subjects. In the order of written informed consent, screening number will be assigned to these subjects, and their baseline information will be collected. Information to be documented includes status of written informed consent and date

of consent, gender, date of birth, age, and drinking history.

Drinking history will be assessed based on the volume of alcohol consumed per week within the last 1 month (30 days). Alcohol abuse is defined as alcohol consumption exceeding 21 units* per week.

* 1 unit: alcohol 8~12 g = one glass of soju
  - One bottle of 21% soju = 6.7 glasses of soju, 21 units = approximately 3 bottles of soju
  - One 500 cc glass of beer (4%) = 1.8 glasses of soju, 21 units = approximately 11 glasses of beer
  - One big glass of wine = 2 glasses of soju, one small glass of wine = 1 glass of soju, 21 units = approximately 10.5 ~ 21 glasses

4.1.3.2 Medical history taking

Details of medical history will be collected via interviews and past medical records and documented. For past medical history including surgical history and allergies within 1 month prior to screening as well as current medical history, time of onset (year and/or month of onset) and status of continuation at the time of screening will be recorded. The following medical history will be collected regardless of time of onset: history of SARS-CoV, MERS-CoV and/or SARS-CoV-2 infection, history of hypersensitivities to vaccinations such as Guillain-Barré syndrome, history of allergy or hypersensitivity reactions to any of the components of the IP, history of SAEs with and/or allergy to antibiotics and/or non-steroidal anti-inflammatory drugs, and history of thrombocytopenia or other coagulation disorders.

4.1.3.3 Medication history taking

Medication history within 1 month prior to screening (prior drugs and concomitant drugs) and details of medication (dosage and administration, duration of treatment, etc.) will be collected. As part of the exclusion criteria, history of vaccination against SARS-CoV, MERS-CoV, or SARS-CoV-2 will be collected regardless of time of vaccination. As for history of treatment with other IPs and/or other investigational devices, antipsychotics, and opioid analgesic dependence, information within 6 months will be collected. As for history of systemic steroids, immunosuppressants/immune modifying drugs, anticancer therapy, and immunoglobulin or other blood transfusions, information within 3 months will be collected.

Any change in medication history from screening will be checked; details of any change in concomitant medication must be recorded in the source document.

4.1.3.4 Height and weight measuring

Height and body weight will be measured at screening. Before measuring body weight, the subject will be instructed to take off outer clothing and shoes and to empty pockets.

4.1.3.5 Chest X-ray and electrocardiogram

At the screening visit, posterior-anterior X-ray and electrocardiogram (ECG) will be performed. During the screening period, one re-test may be performed at the discretion of the investigator.

4.1.3.6 COVID-19 diagnostic test and antibody test

At the screening visit and Visit 2, subjects will be tested for COVID-19 for the inclusion/exclusion criteria checking. Using upper respiratory tract sampling (oropharyngeal/ nasopharyngeal swab) and lower respiratory tract sampling (an additional sputum test will be performed in the presence of symptoms such as cough and sputum), reverse transcription-polymerase chain reaction (RT-PCR) diagnostic test and COVID-19 antibody test (positivity to IgM and IgG; 20 μL of blood) will be performed to determine past and/or current COVID-19 infection. During screening, antibody test will be performed as clinical laboratory test and at Visit 2, the test may be performed by the institution or the COVID-19 diagnostic kit provided by the sponsor.

Subjects who have the following symptoms of COVID-19 infection after randomization should follow the guideline on COVID-19 by ‘Central Disease Control Headquarters · Central Disaster Management. Headquarters’ and inform the symptoms to the investigator. As much as possible, the subjects should be instructed to visit the institution and have medical treatment and diagnosis.

- ≥37.5°C fever or chills, fatigue, myalgia or body aching, headache, sore throat, anosmia and ageusia, nasal obstruction or nasal discharge, nausea or vomiting, diarrhea, cough, respiratory distress or respiratory disturbance

4.1.3.7 Pregnancy test

At the screening visit, prior to the 2nd dose of the IP (Visit 5), at the end of main study visit (Visit 9), and at the end of follow-up visit (Visit 11), all women of childbearing potential, except for surgically sterile women and post-menopausal women (including surgical menopause), will be tested for pregnancy by urine or serum hCG (approximately 5 mL of blood) testing. Women of childbearing potential are defined as those who had menarche and are not surgically sterile (hysterectomy, bilateral tubal ligation, or bilateral ovariectomy) or postmenopausal. Menopause is defined as having no menses for ≥12 months from the final menstrual period without an alternative cause.

4.1.3.8 Re-screening

One re-screening can be performed on those who fail in screening. For re-screening, a new written informed consent will be obtained, a new screening number will be granted, and all of the Visit 1 procedures will be carried out.

4.2 Procedures by visit

The same procedures will be applied to Part A and Part B. Subjects participating in this study must comply with 「Guidelines for the prevention of COVID-19」 presented by Korea Centers for Disease Control & Prevention.

4.2.1 Visit 1 (screening, Day -7 ~ Day -1)

Once prior-consenting procedures are completed and written informed consent is obtained, the subject will be granted a screening number and assessed as follows. Written informed consent can be obtained prior to Visit 1; if all tests are not done at Visit 1, the subject may have another visit for the tests before the randomization at Visit 2.

- Demographic information and medical history (past medical history including surgical history and current medical history) as well as prior and concomitant medications will be taken/documented.

- Vital signs will be measured.
- Physical examination will be conducted.
- Height and weight will be measured.
- Chest X-ray and ECG will be performed.
- Clinical laboratory tests (hematology, blood chemistry, urinalysis, blood coagulation, virus) will be performed.
- Pregnancy test will be performed for women of childbearing potential.
- RT-PCR diagnostic test and antibody test for COVID-19 will be performed.
- The inclusion/exclusion criteria will be checked.
- The next visit day will be scheduled.

4.2.2 Visit 2 (1st dose of the IP, Day 0)

This visit will take place within 7 days after the initial visit. Procedures to be carried out during this visit are as follows. For those who meet all of the inclusion/exclusion criteria at screening, Visits 1 and 2 can simultaneously be conducted with overlapping procedures done only once. In this case, baseline test results will be replaced with screening test results for the purpose of analysis.

- Any change from the last visit in medical history and prior/concomitant medications will be checked.
- Vital signs will be measured.
- Physical examination will be conducted.
- RT-PCR diagnostic test and antibody test for COVID-19 will be performed.
- Based on overall results of all tests and evaluations performed up to this point including clinical laboratory tests, a final decision will be made on the eligibility of the subject according to the inclusion/exclusion criteria.
- Subjects will be randomized in the order of enrollment and given a randomization number.
- Prior to dosing, a blood sample will be collected for immunogenicity testing and the time of blood sampling will be recorded.
- The IP will be administered and the date and time of administration will be recorded
- The subject will be monitored for 30 minutes post-dose for immediate AEs (anaphylaxis associated).
- The subject will be provided with a ruler and a digital thermometer (for tympanic temperature). The subject will also be given a subject diary and instructions on how to

complete the diary in order to collect and monitor solicited and unsolicited AEs.
- The next visit day will be scheduled.

4.2.3 Visit 3 (telephone visit, 2 ~ 5 days after the 1st dose)

This visit will take place by a telephone call made by a trained sub-investigator to the subject between 2 days and 5 days after the 1st dose of the IP.

- Any AEs and any change in concomitant medications since the last visit will be checked.
- The subject will be instructed to record any solicited and unsolicited AEs.
- The next visit day will be scheduled.

4.2.4 Visit 4 (interim visit for the main study, 7 + 3 days after the 1st dose)

This visit will take place on Day 7 after the 1st dose of the IP. Procedures to be carried out during this visit are as follows.

- Any change from the last visit in concomitant medications will be checked.
- The subject diary will be retrieved for status and details of AEs.
- Vital signs will be measured.
- Physical examination will be conducted.
- Clinical laboratory tests (hematology, blood chemistry, urinalysis) will be performed.
- The subject will be given a subject diary and instructions on how to complete the diary.
- The next visit day will be scheduled.

4.2.5 Visit 5 (2nd dose of the IP, 21 + 5 days after the 1st dose)

This visit will take place on Day 21 after the 1st dose of the IP. Procedures to be carried out during this visit are as follows.

- Any change from the last visit in prior and concomitant medications will be checked.
- The subject diary will be retrieved for status and details of AEs.
- Vital signs will be measured.
- Physical examination will be conducted.
- Pregnancy test will be performed for women of childbearing potential.

- Prior to dosing, a blood sample will be collected for immunogenicity testing and the time of blood sampling will be recorded.
- Contraindications for the 2nd dose of the IP will be checked. If the subject meet the criteria of contraindications for the 2nd dose of the IP, subjects will be treated in accordance with section 5.4 'Criteria for subject discontinuation and withdrawal' and proceeded with the procedure of Visit 9.
- The IP will be administered and the date and time of administration will be recorded.
- The subject will be monitored for 30 minutes post-dose for immediate AEs (anaphylaxis associated).
- The subject will be given a subject diary and instructions on how to complete the diary.
- The next visit day will be scheduled.

If the following symptoms are observed in a subject planned to have the 2nd dose, dosing can be rescheduled within the visit window.

- ≥37.5°C fever
- Acute infectious disease requiring delayed IP dosing in the opinion of the investigator

4.2.6 Visit 6 (telephone visit, 2 ~ 5 days after the 2nd dose)

This visit will take place to the subject between 2 days and 5 days after the 2nd dose of the IP via phone call made by a trained sub-investigator.

- Any AEs and any change in concomitant medications since the last visit will be checked.
- The subject will be instructed to record any solicited and unsolicited AEs.
- The next visit day will be scheduled.

4.2.7 Visit 7 (interim visit for the main study, 7 + 3 days after the 2nd dose)

This visit will take place on Day 7 after the 2nd dose of the IP. Procedures to be carried out during this visit are as follows.

- Any change from the last visit in concomitant medications will be checked.
- The subject diary will be retrieved for status and details of AEs.
- Vital signs will be measured.
- Physical examination will be conducted.

- Clinical laboratory tests (hematology, blood chemistry, urinalysis) will be performed.
- The subject will be given a subject diary and instructions on how to complete the diary.
- The next visit day will be scheduled.

4.2.8 Visit 8 (interim visit for the main study, 21 + 5 days after the 2nd dose)

This visit will take place on Day 21 after the 2nd dose of the IP. Procedures to be carried out during this visit are as follows.

- Any change from the last visit in concomitant medications will be checked.
- The subject diary will be retrieved for status and details of AEs.
- Vital signs will be measured.
- Physical examination will be conducted.
- A blood sample will be collected for immunogenicity testing.
- The subject will be given a subject diary and instructions on how to complete the diary.
- The next visit day will be scheduled.

4.2.9 Visit 9 (end of main study visit, 28 + 7 days after the 2nd dose)

This visit will take place on Day 28 after the 2nd dose of the IP. Procedures to be carried out during this visit are as follows. The following procedures are also applicable to those who discontinue the study prior to Visit 9. Subsequently, if consent is obtained from the subject, telephone visits will be conducted by a trained sub-investigator who will call the subject at the time points of Visit 10 (Week 26 after the 2nd dose of the IP) and Visit 11 (Week 52 after the 2nd dose of the IP) for serious ADRs (SADRs) and AEs of special interest (AESIs).

- Any change from the last visit in concomitant medications will be checked.
- The subject diary will be retrieved for status and details of AEs.
- Vital signs will be measured.
- Physical examination will be conducted.
- Clinical laboratory tests (hematology, blood chemistry, urinalysis) will be performed.
- Pregnancy test will be performed for women of childbearing potential.
- The next visit day will be scheduled.

4.2.10 Visit 10 (interim visit for follow-up, 26 ± 2 weeks after the 2nd dose)

This visit will take place at Week 26 after the 2nd dose of the IP. Procedures to be carried out during this visit are as follows.

- Any SAEs and AESIs since last visit will be checked. If the subject experienced SAEs and AESIs, concomitant medications given for these events will also be documented.
- Vital signs will be measured.
- A blood sample will be collected for immunogenicity testing.
- The next visit day will be scheduled.

4.2.11 Visit 11 (end of follow-up visit, 52 ± 4 weeks after the 2nd dose)

As the final study visit, this visit will take place at Week 52 after the 2nd dose of the IP. Those who are withdrawn after Visit 9 will perform the following procedures except for blood sampling for immunogenicity testing, and if consent is obtained from the subject, telephone visits may be conducted by a trained sub-investigator who will call the subject at the time points of Visit 10 (Week 26 after the 2nd dose of the IP) and Visit 11 (Week 52 after the 2nd dose of the IP) for SADRs and AESIs.

- Any SAEs and AESIs since last visit will be checked. If the subject experienced SAEs and AESIs, concomitant medications given for these events will also be documented.
- Vital signs will be measured.
- Pregnancy test will be performed for women of childbearing potential.
- A blood sample will be collected for immunogenicity testing.

4.2.12 Unscheduled visits

Besides scheduled visits, additional visits may be conducted any time upon the request of subjects or their representatives or whenever deemed necessary by the investigator. The investigator will remind the subjects of this during their regular study visits and instruct them to immediately contact the investigator in the event of AEs. Unscheduled visits should not affect the schedules of regular visits. During unscheduled visits, tests can be done as necessary at the discretion of the investigator.

5. Subject selection and withdrwawl criteria

5.1 Inclusion criteria

To be eligible for enrollment, individuals must meet all of the criteria described below.

1) Individuals who voluntarily decide to participate in this study and provide written informed consent:
   - Healthy male and female adult at the age of 19 to 50 years (Part A)
   - Healthy male and female adult at the age of 19 to 75 years (Part B)
2) Individuals who, after receiving detailed explanations about the study, voluntarily decide to participate in this study and provide written informed consent
3) Individuals who are available for all visit procedures including telephone visits during the study period

5.2 Exclusion criteria

Individuals who meet any of the criteria described below will be excluded from enrollment.

1) COVID-19 positive based on RT-PCR using upper respiratory tract (oropharyngeal, nasopharyngeal) or lower respiratory tract (sputum) sampling or COVID-19 antibody (IgM and/or IgG) positive
2) History of SARS-CoV, MERS-CoV or SARS-CoV-2 infection
3) Increased risk of exposure to SARS-CoV-2 (e.g., healthcare workers in direct contact with patients with COVID-19)
4) History of vaccination against SARS-CoV, MERS-CoV, or SARS-CoV-2
5) Immune system disorders including immunodeficiency disease
6) Planned blood donation or transfusion during the study period
7) Planned administration of other vaccines from 4 weeks before the 1st dose to 4 weeks after the 2nd dose of the IP
8) Height and/or weight measurements at screening:
   ① Body weight <40 kg or > 100 kg
   ② Body mass index <18 kg/m^2 or > 30 kg/m^2
9) Clinically significant abnormalities in clinical laboratory test, ECGs and chest X-ray during screening in the opinion of the investigator (e.g., Wolff-Parkinson-White syndrome)

10) Any planned surgery during the study period
11) Fever (≥ 37.5°C) within 3 days prior to screening or serious acute (acute fever, cough, respiratory distress, chills, myalgia, headache, sore throat, anosmia, or ageusia) or chronic infection within 7 days prior to screening (requiring systemic antibiotics or antivirals)
12) Evidence or history of serious acute, chronic, or progressive disease (e.g., cancer, diabetes mellitus, chronic pulmonary disease, acquired immune deficiency syndrome (AIDS), blood dyscrasias, or immune system, urinary system, mental, musculoskeletal system, cardiovascular system, respiratory system, endocrine, nervous system, hepatobiliary system, renal disorders, etc.) which, in the opinion of the investigator, makes the individual ineligible for the study
13) Positive serum tests during screening [type B hepatitis, human immunodeficiency virus (HIV), type C hepatitis]
14) History of treatment with antipsychotics or opioid analgesic dependence within 6 months prior to IP dosing
15) History of severe allergic reactions (e.g., anaphylaxis, Guillain-Barré Syndrome) or severe hypersensitivity reactions to the IP or any of its components
16) History of therapy that might affect immunity: treatment with immunosuppressants or immune modifying drugs, anticancer therapy, or radiotherapy within 3 months prior to screening
17) History of systemic steroids (prednisone ≥10mg/day for > 14 consecutive days) within 3 months prior to screening. Topical, inhaled, and intranasal corticosteroids are allowed regardless of dose.
18) Past treatment within 3 months prior to screening, or planned treatment during the study period, with immunoglobulin or blood derivatives
19) Individual with thrombocytopenia or other coagulation disorders for whom intramuscular (IM) injections are contraindicated or individual who is on anticoagulant therapy*
    * Anticoagulant therapy: continuous use of anticoagulants such as coumarin/warfarin or new oral anticoagulants/antiplatelets
20) History of excessive alcohol consumption or drug addiction
21) Women of childbearing potential who do not agree to use medically allowed methods of contraception* or to be heterosexually inactive until 60 days after the last dose of the IP
    *Hormonal contraceptive, intrauterine device(IUD(intrauterine device) or IUS(intrauterine system)), tubal ligation, double-blocking method(condom for male and female, cervical cap or diaphragm, complex method such as contraceptive sponge), single-blocking method using spermicides
22) Pregnant or breastfeeding woman

23) Treatment with other IPs within 6 months prior to participation in this study
24) The investigator who is directly related to this study or sub-investigator/study coordinator who is supervised by investigator or their family member
25) Other reasons including medical reasons based on which the individual is considered to be ineligible for this study in the opinion of the investigator

5.3 Contraindications for the 2nd dose

Subjects who meet any of the following criteria cannot have the 2nd dose of the IP and will be treated in accordance with section 5.4 'criteria for subject discontinuation and withdrawal'.

1) The subject meets any of the criteria below after the 1st dose of the IP based on which the subject is ineligible for the 2nd dose in the opinion of the investigator.
   ① COVID-19 positive based on RT-PCR diagnostic testing or SARS-CoV-2 infection positive based on antibody testing
   ② Requiring prohibited drugs
   ③ Development of thrombocytopenia or other coagulation disorders
2) Anaphylaxis or unexpected generalized hypersensitivity reaction after the 1st dose of the IP
3) SAEs relate to the IP
4) Pregnancy
5) Other clinically significant condition due to which the subject is ineligible for the 2nd dose in the opinion of the investigator

5.4 Criteria for subject discontinuation and withdarwawl

Those who are randomized but cannot participate in the study for the entire period for any reason will be classified as dropouts. Subjects may choose to discontinue the study at any time for any reason by requesting withdrawal. Or subjects may also be discontinued at any time for safety, behavioral, or administrative reasons at the discretion of the investigator or the sponsor. The investigator should ask about reasons for withdrawal and ask the subject to return for the last visit. If applicable, the investigator should make every effort to follow up on unresolved AEs.

For the following reasons, subjects may ask for withdrawal or may be discontinued or withdrawn

upon discussion with the investigator and the sponsor. In this case, the reasons and actions taken for the discontinuation and withdrawal should be recorded on the relevant document as well as on the CRF:

① The subject or his/her representative withdraws informed consent.
② Violation of the inclusion/exclusion criteria is identified during the course of the study.
③ The subject corresponds to exclusion criteria for 2nd dose.
④ The subject or his/her representative wants to discontinue the study due to unsatisfactory therapeutic effects during the study period.
⑤ The subject does not comply with instructions of the principal investigator or the sub-investigator.
⑥ Lost to follow-up.
⑦ The subject becomes pregnant during the study period.
⑧ Study participation is inappropriate for other reasons based on the judgment of the sponsor or the investigator

If possible, subjects who are withdrawn after Visit 2 and before Visit 9 (end of main study visit) will return to the institution for Visit 9 procedures. If possible, those who are withdrawn after Visit 9 will return to the institution for Visit 11 (end of follow-up visit) procedures except for blood sampling for immunogenicity testing. For safety follow-ups for dropouts, if consent is obtained from the subject, telephone visits will be conducted by a trained sub-investigator who will call the subject at the time points of Visit 10 (Week 26 after the 2nd dose of the IP) and Visit 11 (Week 52 after the 2nd dose of the IP) for SADRs and AESIs.

5.5 Criteira for study suspension

During the study period, subjects will be monitored for the following ADRs that serve as criteria for study suspension. In the event of ADRs that are the criteria for study suspension, additional enrollment and treatment will be stopped at this time, and the DSMB will meet to decide study continuation.

1) Part A study: Grade ≥3 ADRs occurring within 7 days after the 1st dose in the first 5 subjects
2) Part A and Part B studies:
   ① Grade ≥3 ADRs occurring within 3 days (Day 0 ~ Day 2) from the respective dosing day and lasting for >1 day despite active intervention for resolution in ≥15% of all

subjects planned to be enrolled to each study group (Part A: ≥8/50 subjects, Part B: ≥ 35/230 subjects)

② SADRs or Grade ≥4 ADRs occurring within 28 days after the respective dose of the IP

5.6 Study compliance and handling of protocol deviations

To prevent protocol deviations, the principal investigator and the sub-investigator should be fully familiar with, and thoroughly comply with the protocol. To comply with IP dosing and testing schedules in this study, the sub-investigator will take adequate measures to ensure that subjects return for all outpatient visits by, for instance, issuing a written notice of the next visit time or performing telephone monitoring. Should protocol deviations occur, they will be notified to the responsible clinical research associate (CRA) or the sponsor and reported within the timeline as appropriate according to the Institutional Review Board (IRB)-specific regulations.

6. Investigational product

6.1 Overview of investigational product

6.1.1 Test drug

6.1.1.1 Low dose group

- Code name: EuCorVac-19 (COVID-19 recombinant protein vaccine)
- Appearance and formulation: clear, yellow-green liquid in a clear, colorless vial for injection
- Storage condition: to be refrigerated at 2~8℃
- Shelf-life: up to 12 months from the date of manufacture (to be extended based on the stability test results if available)
- Drug substance and its content:

per 1 vial(0.5 mL)

Purpose	Drug substance	Specification	Content	Unit
**Active ingredient**				
Main raw material	RBD Antigen	In-house	10	μg
**Excipients**				
Solubilizing agent	EcML	In-house	10	μg
Solubilizing agent	CoPoP	In-house	20	μg
Solubilizing agent	DOPC	In-house	0.4	mg
Solubilizing agent	Cholesterol	EP	0.1	mg
Buffer	Disodium hydrogen phosphate heptahydrate	USP	0.36	mg
Buffer	Potassium dihydrogen phosphate	EP	0.11	mg
Isotonic agent	Sodium chloride	EP	4.50	mg
Solvent	Water for injection	EP	q.s.	

6.1.1.2 High dose group

- Code name: EuCorVac-19(COVID-19 recombinant protein vaccine)
- Appearance and formulation: clear, yellow-green liquid in a clear, colorless vial for injection
- Storage condition: to be refrigerated at 2~8℃
- Shelf-life: up to 12 months from the date of manufacture (to be extended based on the stability test results if available)
- Drug substance and its content:

In one vial

Purpose	Drug substance	Specification	Content	Unit
**Active ingredient**				
Main raw material	RBD Antigen	In-house	20	μg
**Excipients**				
Solubilizing agent	EcML	In-house	20	μg
Solubilizing agent	CoPoP	In-house	40	μg
Solubilizing agent	DOPC	In-house	0.8	mg
Solubilizing agent	Cholesterol	EP	0.2	mg
Buffer	Disodium hydrogen phosphate heptahydrate	USP	0.36	mg
Buffer	Potassium dihydrogen phosphate	EP	0.11	mg
Isotonic agent	Sodium chloride	EP	4.50	mg
Solvent	Water for injection	EP	q.s.	

6.1.2 Placebo (for Part B only)

- Product name: Dai Han Isotonic Sodium Chloride Inj. (sodium chloride)
- Appearance and formulation: clear, colorless, slightly salty solution in a clear, colorless a plastic) container of water for injection
- Storage condition: store in a sealed container at ambient temperature (1~30℃)
- Shelf-life: 36 months from manufacturing date
- Composition(per 20mL):Sodium chloride(active ingredient) 180 mg, water for injection(excipient)

6.2 Dosage, method, and duration of treatment

Subjects will receive 0.5mL of either the low or the high dose of the IP(EuCorVac-19) or placebo(Dai Han isotonic sodium chloride inj.) on the deltoid muscle for two times. The 2nd dose will be given 21 days after the 1st dose.

The IP will be prepared according to the IP manual for administration to the subject. Prior to dosing, the appearance of the IP will be examined; the IP with any abnormalities will not be administered. If the filled volume of the IP and placebo exceeds the recommended dosage, the remaining substances will be discarded in accordance with the regulation of the institution or the guidance provided by the sponsor.

6.3 Manufacturing, packaging, and labeling of the investigational product

The IP will be manufactured or purchased by the sponsor and supplied to the pharmacist at the study institution.

The label of the IP will be prepared according to "Regulations for the Manufacturing and Quality Control of Medicinal Products, Attachment 11. Manufacturing of Investigational Products" and bear the following information.

1. For clinical investigation only (e.g., "for clinical trials")
2. Name or identification of the IP
3. Batch no. or code no. to identify the content and packaging
4. Sponsor (IND holder)'s name, address, and telephone number
5. Expiry date (shelf-life)
6. Storage conditions
7. Reference code to identify the study
8. If necessary, subject identification no., treatment no., visit no., investigator's name, method of application

If the primary package is too small to bear all of the above-mentioned information, minimum information (for clinical trials/name of the IP or identification no./batch no. or code

no./sponsor/reference code/study drug no./shelf-life) will be labeled on the primary package, and all information will be labeled from the secondary package according to Appendix 11 of Regulations for the Manufacturing and Quality Control of Medicinal Products.

6.4 Management of the investigational product

- The test drug will be stored at 2~8°C, and the comparator (placebo) will be stored at ambient temperature (1~30°C). The study drug cannot be used without the instruction (prescription) of the principal investigator and the sub-investigator.
- The sponsor should supply the IP directly to the pharmacist upon agreement with the principal investigator and should maintain the receipt.
- The pharmacist should ensure that the IP is not used for any purpose other than the clinical trial by properly storing and managing the IP. The pharmacist is responsible for receiving the IP, maintaining the inventory, dispensing the IP to each subject, and returning the IP. The pharmacist should document relevant records and inform the principal investigator on a regular basis.
- During the course of the study, the sponsor should check the quantity and storage conditions of the IP and take necessary actions for appropriate study conduct.
- In the event of suspension or termination of the study or any issues with the IP or expiration of the IP, the sponsor will retrieve and destroy any unused IPs. In this case, the pharmacist should return all unused IPs to the sponsor and maintain the return receipt.
- During the study, the container of the IP dispensed to the subject and used will be immediately destroyed on site upon use according to site-specific regulations and guidelines provided by the sponsor, and relevant document should be maintained.

6.5 Mantaining and breaking the blind

Part B is an observer-blind study. To maintain the quality of blinding, unblinded personnel belonging to the institution (person in charge of preparation for IP administration, person in charge of IP administration, pharmacist, CRA) will be designated. The unblinded personnel should perform only pre-defined tasks related to the study. In addition, a separate unblinded CRA will check the quantity and dispensing of the IP at the institution. The responsible unblinded CRA should perform only pre-defined tasks related to the study such as pharmacy monitoring and manufacturing and administration monitoring and should document the independent tasks. As a result, other investigators and sub-investigators, except for the unblinded personnel, will remain

blinded during the study period. The unblinded personnel in charge of preparation for IP administration and in charge of IP administration will prepare the IP in a place where the subject is not present and ask the subject to turn away at the time of IP administration so that the subject remains blinded during IP administration.

Allocation of unique codes by group will be managed by IWRS at the central enrollment center. In principle, randomization codes must remain blinded until the data lock for the analysis of the main study until the end of study and completion of analyses by the central laboratory so that assignment of each subject to relevant treatment group remains blinded. See Section 7.3.7 in this protocol for maintaining the blind during interim analysis.

If it is necessary to access a code during the study due to an SADR or the sponsor decides it is essential to access the code for the safety and right of the subject, the double blind may be broken, and blinding will be managed to ensure that only the unique code of the relevant subject is accessed.

Unblinding must be considered case by case and only in the event of a serious medical emergency in which knowledge of the study drug administered is necessary for appropriate treatment. In general, blind must be broken only if information on the treatment group may affect the subject's treatment. If the principal investigator or the sponsor considers that unblinding is necessary, the blind will be broken after contacting the sponsor or the principal investigator and obtaining their agreement. All unblinding must be clearly accounted for and documented. Subjects whose randomization codes are unblinded can no longer participate in the study.

6.6 Concomitant medication and concomitant therapy

For drugs administered concomitantly from Visit 1 to Visit 9 for treatment and diagnosis of other diseases or AEs, details of concomitant medication (product name, purpose of administration, dose and unit, duration of treatment [start date, stop date], route of administration, etc.) should be recorded in the case report form (CRF). During the period from after Visit 9 to Visit 11, only drugs administered for SAEs or AESIs will be documented.

Information on concomitant treatment (concomitant medication or therapy) given for treatment and diagnosis of other diseases or AEs during the study period should be recorded in the CRF.

For concomitant medication, product name, purpose of administration, dose and unit, duration of treatment (start date, stop date), and route of administration will be documented. For concomitant therapy, name of therapy, purpose, and duration (start date, stop date) will be documented.

Drugs listed below are prohibited for concomitant use during the period from Visit 1 to Visit 9.

(1) Vaccines within 28 days before and after IP dosing
(2) Immunosuppressants or immune modifying drugs
  - Azathioprine, cyclosporine, interferon, G-CSF, tacrolimus, everolimus, sirolimus, cyclophosphamide, 6-mercaptopurine, methotrexate, rapamycin, leflunomide, etc.
  - Long term use of steroids: prednisone ≥10 mg/day for ≥14 consecutive days (Topical or inhaled steroids, intranasal spray, and eye drops are allowed regardless of dose.)
(3) Blood or blood derivatives (including immunoglobulin)
(4) Anticoagulant therapy: continuous use of anticoagulants such as coumarin/warfarin or new oral anticoagulants/antiplatelets
  - New oral anticoagulants (direct oral anticoagulant [DOAC]): dabigatran, rivaroxaban apixaban, edoxaban
  - New antiplatelets: prasugrel, ticagrelor, cangrelor

Drugs listed below are prohibited for concomitant use throughout the study period (Visit 1 to Visit 11).

(1) Other coronavirus vaccines
(2) Other IPs

Other concomitant drugs for treatment or examination of other diseases that are considered to have no impact on the interpretation of study results will be allowed at the discretion of the investigator.

7. Statistical considerations

7.1 Sample size and rationale for sample size calcuiaton

7.1.1 Sample size

7.1.1.1 Part A

To obtain 25 subjects per dose group who have at least one dose of the IP after randomization, at least 50 eligible volunteers based on the inclusion/exclusion criteria will be enrolled.

	**Low dose group**	**High dose group**
Number of subjects in test group	15 subjects	15 subjects
Number of subjects in placebo comparator group	10 subjects	10 subjects
Total number of subjects	25 subjects	25 subjects

7.1.1.2 Part B

To obtain 195 subjects who have at least one dose of the IP after randomization and are available for immune response analysis, at least 230 eligible volunteers based on the inclusion/exclusion criteria will be enrolled considering the drop-out rate of 15%. At least 15% (35 subjects) of overall subjects will be recruited from those between 51 and 75 years of age.

	**Low dose group**	**High dose group**	**Placebo comparator group**
Number of immune response analyses	85 subjects	85 subjects	25 subjects
Total number of subjects considering drop-out rate of 15%	100 subjects	100 subjects	30 subjects

7.1.2 Rationale for sample size calculation

Because this is the first time that the IP is administered to humans and this exploratory study is not intended to test a statistical hypothesis, sample size was not statistically calculated.

7.1.3 Replacement of Study Subject

If the first 5 subjects in each dose group in Part A for safety assessment are decided to discontinue or withdraw the study without administration of IP after randomization, additional subject can be enrolled for replacement of discontinued or withdrawn subjects. These additional subjects will be allocated in identical group in which the subject who decided to discontinue the study was randomized using IWRS.

7.2 Endpoints

7.2.1 Safety endpoints

- AEs
  ① Immediate AEs occurring within 30 minutes after each IP dosing
  ② Solicited local and systemic AEs occurring for 7 days(Day 0~ Day 6) after each IP dosing
    ✓ Local AEs : pain, tenderness, erythema/redness, induration/swelling, itchiness
    ✓ Systemic AEs : fever, fatigue/malaise, chills/rigors, headache, myalgia, joint pain, diarrhea, vomiting, abdominal pain, mucocutaneous reaction/rash, cough, acute bronchospasm, respiratory distress
  ③ Unsolicited AEs occurring within 28 days after the last IP dosing
  ④ SAEs occurring within 52 weeks after the last IP dosing
  ⑤ AESIs occurring within 52 weeks after the last IP dosing
- Clinical laboratory tests (hematology/blood chemistry, urinalysis)
- Vital signs
- Physical examination

7.2.2 Immunogenicity endpoints

7.2.2.1 Part A

- GMT and GMFR of antigen protein-specific antibodies as measured by ELISA at pre-dose (Visit 2), on Day 21 (Visit 5) after the 1st dose, and on Day 21 (Visit 8), Week 26 (Visit 10), and Week 52 (Visit 11) after the 2nd dose of the IP
- Proportion of subjects with seroconversion to antigen protein-specific antibodies as measured

by ELISA on Day 21 (Visit 5) after the 1st dose and on Day 21 (Visit 8), Week 26 (Visit 10), and Week 52 (Visit 11) after the 2nd dose of the IP (Seroconversion rate [SCR]) †

† Seroconversion is defined as a ≥4-fold increase in GMT post-dose of the IP from pre-dose.

- GMT and GMFR compared to the baseline of neutralizing antibodies to SARS-CoV-2 as analyzed by Focus Reduction Neutralization Test (Wild-type SARS-CoV-2) at pre-dose (Visit 2), on Day 21 (Visit 5) after the 1st dose, and on Day 21 (Visit 8), Week 26 (Visit 10), and Week 52 (Visit 11) after the 2nd dose of the IP
- Proportion of subjects with seroconversion to SARS-CoV-2 neutralizing antibodies as analyzed by Focus Focus Reduction Neutralization Test (Wild-type SARS-CoV-2) at pre-dose (Visit 2), on Day 21 (Visit 5) after the 1st dose, and on Day 21 (Visit 8), Week 26 (Visit 10), and Week 52 (Visit 11) after the 2nd dose of the IP (Seroconversion rate [SCR])‡

  ‡ Seroconversion is defined as a ≥4-fold increase in GMT post-dose of the IP from pre-dose

7.2.2.2 Part B

- GMT and GMFR of antigen protein-specific antibodies as measured by ELISA at pre-dose (Visit 2), on Day 21 (Visit 5) after the 1st dose, and on Day 21 (Visit 8), Week 26 (Visit 10), and Week 52 (Visit 11) after the 2nd dose of the IP
- Proportion of subjects with seroconversion to antigen protein-specific antibodies as measured by ELISA on Day 21 (Visit 5) after the 1st dose and on Day 21 (Visit 8), Week 26 (Visit 10), and Week 52 (Visit 11) after the 2nd dose of the IP (seroconversion rate [SCR])†

  † Seroconversion is defined as a ≥4-fold increase in GMT post-dose of the IP from pre-dose.
- GMT and GMFR compared to the baseline of neutralizing antibodies to SARS-CoV-2 as analyzed by Focus Reduction Neutralization Test (Wild-type SARS-CoV-2) at pre-dose (Visit 2), on Day 21 (Visit 5) after the 1st dose, and on Day 21 (Visit 8), Week 26 (Visit 10), and Week 52 (Visit 11) after the 2nd dose of the IP
- Proportion of subjects with seroconversion to SARS-CoV-2 neutralizing antibodies as analyzed by Focus Focus Reduction Neutralization Test (Wild-type SARS-CoV-2) at pre-dose (Visit 2), on Day 21 (Visit 5) after the 1st dose, and on Day 21 (Visit 8), Week 26 (Visit 10), and Week 52 (Visit 11) after the 2nd dose of the IP (Seroconversion rate [SCR])‡

  ‡ Seroconversion is defined as a ≥4-fold increase in GMT post-dose of the IP from pre-dose

7.2.2.3 Exploratory assessment (Part A & Part B)

- T cell immune response markers as analyzed by Ex vivo IFNγ ELISpot at pre-dose (Visit 2), on Day 21 (Visit 5) after the 1st dose, and on Day 21 (Visit 8) after the 2nd dose of the IP

7.3 Statistical analysis plan

7.3.1 Analysis sets

Safety data obtained from the study subjects will primarily be analyzed in the safety set while immunogenicity data will be analyzed primarily in the per-protocol set (PPS) and additionally in the full analysis set (FAS).

Analysis sets are defined as follows.

(1) Safety Set:
Includes all subjects who had at least one dose of the IP.

(2) FAS (Full Analysis Set):
Includes all subjects who had at least one dose of the IP, pre-dose immunogenicity data and at least one post-dose immunogenicity endpoint data available. Hence, the number of subjects may vary depending on the number of immunogenicity endpoints.

(3) PPS (Per-Protocol Set):
Includes all subjects in the FAS who completed the study through to Visit 9 (end of main study visit) according to the protocol. In the follow-up analysis, those who completed the study through to the end of follow-up visit (Visit 11) will separately be analyzed. Subjects with any of the following protocol deviations may be excluded from the PPS.

<u>Major protocol deviations</u>
① Informed consent form (ICF) not obtained
② Violation of the inclusion/exclusion criteria
③ Concomitant treatment with prohibited drugs during the study period
④ Baseline and Visit 8 immunogenicity tests not done
⑤ Randomization errors

For subjects who are considered to have any major protocol deviations other than the above listed,

a blind meeting will be held prior to database (DB) lock in which a decision will be made on whether to exclude the subjects from the PPS based on comprehensive assessment of the impact of the deviations on the study.

7.3.2 General principles of statistics

For continuous data, descriptive statistics (number of subjects, mean, SD, minimum, median, maximum) will be presented. For categorical data, frequency and percentage will be presented, and if necessary, 95% two-sided confidence interval (CI) will also be calculated.

7.3.3 Analysis of demographics and baseline characteristics

In terms of demographics and baseline characteristics, mean, SD, minimum, and maximum will be presented by treatment group for continuous data; and frequency and percentage will be presented by treatment group for categorical data.

7.3.4 Safety analysis (Part A & Part B)

Results from Part A and Part B will be presented by phase of study.

7.3.4.1 Adverse events

Summary descriptions will be provided as below for details of treatment emergent AEs (TEAEs).

Frequency, incidence, and number of events will be presented for solicited local and systemic AEs observed for 7 days(Day 0~Day 6) after each IP dosing, immediate AEs (anaphylaxis related) occurring within 30 minutes after each IP dosing, and unsolicited AEs occurring within 28 days after the last IP dosing. By treatment group, type of AEs will be classified by severity, causal relationship, outcome, etc., and frequency, incidence, and number of events will be presented by category.

Additionally, frequency, incidence, and number of events will be presented for SAEs and AESIs collected within 52 weeks after the 2nd dose of the IP.

All AEs except for solicited local and systemic AEs will be classified by system organ class and preferred term of Medical Dictionary for Regulatory Activities.

7.3.4.2 Clinical laboratory tests

For hematology/blood chemistry as part of clinical laboratory tests, descriptive statistics will be presented by item and by visit; and for each item, descriptive statistics will be presented for change from before the 1st dose of the IP (screening) at each visit post-dose.

For each clinical laboratory test (hematology/blood chemistry/urinalysis), subjects who had a shift from normal/not clinically significantly (NCS) abnormal findings before the 1st dose of the IP (screening) to clinically significantly (CS) abnormal findings post-dose of the IP at least once will be summarized as frequency and percentage.

7.3.4.3 Vital signs

Descriptive statistics will be presented by visit. In addition, descriptive statistics will also be presented for change from before the 1st dose of the IP (Visit 2, baseline) at each time point post-dose.

7.3.4.4 Physical examination

Based on the assessment of physical examination results at each visit versus before the 1st dose of the IP (Visit 2) by treatment group, frequency and percentage will be presented by treatment group for subjects who had a shift from normal/NCS abnormal findings pre-dose to CS abnormal findings post-dose of the IP at least once.

7.3.5 Immunogenicity analysis

7.3.5.1 Part A

(1) GMT and GMFR compared to the baseline(Visit 2) of antigen protein-specific antibodies as measured by ELISA at pre-dose (Visit 2), on Day 21 (Visit 5) after the 1st dose, and on Day 21 (Visit 8), Week 26 (Visit 10), and Week 52 (Visit 11) after the 2nd dose of the IP

GMT of antigen protein-specific antibodies as measured by ELISA at each time point post-dose versus pre-dose (Visit 2) will be calculated, and 95% two-sided CIs of GMT ratio post-dose versus pre-dose as well as GMFR will be presented by treatment group.

(2) Proportion of subjects with seroconversion to antigen protein-specific antibodies as measured by ELISA at pre-dose(Visit 2), on Day 21 (Visit 5) after the 1st dose and on Day 21 (Visit 8), Week 26 (Visit 10), and Week 52 (Visit 11) after the 2nd dose of the IP (seroconversion rate [SCR])

Post-dose SCR to antigen protein-specific antibodies as measured by ELISA at each point as well as its 95% two-sided CI will be presented by treatment group.

(3) GMT and GMFR compared to the baseline of neutralizing antibodies to SARS-CoV-2 as analyzed by Focus Reduction Neutralization Test (Wild-type SARS-CoV-2) at pre-dose (Visit 2), on Day 21 (Visit 5) after the 1st dose, and on Day 21 (Visit 8), Week 26 (Visit 10), and Week 52 (Visit 11) after the 2nd dose of the IP

GMT of neutralizing antibodies to SARS-CoV-2 as analyzed by Focus Reduction Neutralization Test (Wild-type SARS-CoV-2) at each time point post-dose versus pre-dose (Visit 2) will be calculated, and GMT ratio post-dose versus pre-dose as well as its 95% two-sided CI will be presented by treatment group.

(4) Proportion of subjects with seroconversion to neutralizing antibodies to SARS-CoV-2 as analyzed by Focus Reduction Neutralization Test (Wild-type SARS-CoV-2) at pre-dose(Visit 2), on Day 21 (Visit 5) after the 1st dose and on Day 21 (Visit 8), Week 26 (Visit 10), and Week 52 (Visit 11) after the 2nd dose of the IP (seroconversion rate [SCR])

Post-dose SCR to neutralizing antibodies to SARS-CoV-2 as analyzed by Focus Reduction Neutralization Test (Wild-type SARS-CoV-2) at each point as well as its 95% two-sided CI will be presented by treatment group.

7.3.5.2 Part B

(1) GMT and GMFR compared to the baseline(Visit 2) of antigen protein-specific antibodies as measured by ELISA at pre-dose (Visit 2), on Day 21 (Visit 5) after the 1st dose, and on Day

21 (Visit 8), Week 26 (Visit 10), and Week 52 (Visit 11) after the 2nd dose of the IP

GMT of antigen protein-specific antibodies as measured by ELISA at each time point post-dose versus pre-dose (Visit 2) will be calculated, and 95% two -sided CIs of GMT ratio post-dose versus pre-dose as well as GMFR will be presented by treatment group.

(2) Proportion of subjects with seroconversion to antigen protein-specific antibodies as measured by ELISA on Day 21 (Visit 5) after the 1st dose and on Day 21 (Visit 8), Week 26 (Visit 10), and Week 52 (Visit 11) after the 2nd dose of the IP (SCR)

Proportion of subjects with seroconversion to antigen protein-specific antibodies as measured by ELISA at each time point post-dose of the IP and its 95% two-sided CI will be presented by treatment group.

(3) GMT and GMFR compared to the baseline of neutralizing antibodies to SARS-CoV-2 as analyzed by Focus Reduction Neutralization Test (Wild-type SARS-CoV-2) at pre-dose (Visit 2), on Day 21 (Visit 5) after the 1st dose, and on Day 21 (Visit 8), Week 26 (Visit 10), and Week 52 (Visit 11) after the 2nd dose of the IP

GMT of neutralizing antibodies to SARS-CoV-2 as analyzed by Focus Reduction Neutralization Test (Wild-type SARS-CoV-2) at pre-dose (Visit 2) and at each time point post-dose will be calculated, and post-dose to pre-dose ratio as well as its 95% two-sided CI will be presented by treatment group.

(4) Proportion of subjects with seroconversion to neutralizing antibodies to SARS-CoV-2 as analyzed by Focus Reduction Neutralization Test (Wild-type SARS-CoV-2) at pre-dose(Visit 2), on Day 21 (Visit 5) after the 1st dose and on Day 21 (Visit 8), Week 26 (Visit 10), and Week 52 (Visit 11) after the 2nd dose of the IP (SCR)

Post-dose SCR to neutralizing antibodies to SARS-CoV-2 as analyzed by Focus Reduction Neutralization Test (Wild-type SARS-CoV-2) at each point as well as its 95% two-sided CI will be presented by treatment group.

7.3.5.3 Exploratory assessment (Part A & B)

(1) T cell immune response markers as analyzed by Ex vivo IFNγ and IL-4 ELISpot at pre-dose and (Visit 2) on Day 21 (Visit 5) after the 1st dose, and on Day 21(Visit 8) after the 2nd dose of the IP

For T cell immune response markers as analyzed by Ex vivo IFNγ ELISpot at pre-dose (Visit 2) on Day 21(Visit 5) after the 1st dose, and on Day 21 (Visit 8) after the 2nd dose of the IP, number of subjects, mean, SD, median, minimum, and maximum will be presented, and change from pre-dose on Day 21 after the1st and 2nd dose of the IP as well as its 95% two-sided CI will be presented by treatment group.

7.3.6 Handling of drop-outs or missing data

In this study, imputation will not be applied to missing data.

7.3.7 Planned interim analysis and safety monitoring

Upon completion of data collection from all subjects in Part A up to Day 28 after the 2nd dose of the IP (end of main study, Visit 9), DB lock will be carried out for a data analysis based on which interim study results will be presented.

Also, upon completion of data collection from all subjects in Part A up to Week 26 after the 2nd dose of the IP (Visit 10), DB lock will be carried out on those collected data and the interim study results will be presented.

The data from all subjects in Part B up to Day 28 (end of main study, Visit 9) will be collected and DB lock on these data will be carried out for a data analysis. The interim study results will be presented.

Subsequently, once all subjects in Part A and Part B complete the study (end of follow-up, Visit 11), DB lock will be carried out, and the final results will be presented.

For the purpose of safety monitoring, the principal investigator will serve as the protocol safety manager based on the level of risk involved in this study. During the study period, the principal investigator will monitor according to the protocol the safety of subjects participating in the study at the applicable institution.

Whenever an SAE and/or an ADR occurs, the principal investigator will collect and review the data of the relevant subject as well as relevant safety information. In the event of an AE, unexpected issue, or a protocol non-compliance, the principal investigator will report to the sponsor (including the CRO), the IRB, and if necessary, the Ministry of Food and Drug Safety (MFDS) according to the protocol, the IRB-specific regulations, Good Clinical Practice (GCP), and other applicable regulations. In addition, if efficacy and safety information that might have a significant impact on the continuation of the study according to Section 9.11. 'Early termination or suspension of the study' in the protocol becomes known, the principal investigator will report to the sponsor in a timely manner, and in turn, the sponsor will discuss and decide on study continuation according to the sponsor's SOP.

7.3.8 Data and safety monitoring board

To review study data, the DSMB will be established by the sponsor. Members of the DSMB should ensure the integrity of the study and should not be affected by any factor except when necessary to maintain the safety of study subjects. Individuals who clearly have the financial or intellectual ownership of the IP or applicable procedures or any related conflicts of interest cannot be the members of the DSMB. The DSMB consists of a total of 4 individuals: 3 DSMB members including a chair and 1 administrative secretary. Of those, the 3 DSMB members will have the right to vote: 2 infectiology specialists and 1 statistician. The DSMB administrative secretary will perform administrative tasks for the operation of the DSMB and will not have the voting right. For detailed procedures and information, see 'The Charter of Data and Safety Monitoring Board'.

To determine dose escalation and enrollment status of remaining subjects in each low and high dose group in Part A, 'solicited/unsolicited AEs collected for 7 days after the IP' in the first 5 subjects in the low dose group will be assessed after the end of their Day 7 Visit following the 1st dose of the IP, based on which enrollment of the rest of subjects to the low dose group as well as enrollment to the high dose group will be determined.

In addition, upon the end of Day 28 Visit after the 2nd dose of the IP in the last subject in Part A, 'solicited/unsolicited AEs collected for 28 days after the 2nd dose of the IP' and other safety data obtained from all subjects in Part A will be assessed, verified, and analyzed by the DSMB. The DSMB members will review the adverse events of Part A subjects and assess where those AEs are considered as the criteria for discontinuation of the study. The relation between AE and IP will

also be analyzed and whether to proceed to Part B will be determined.

If DSMB meeting is necessary for review of ADR and confirmation of Enhanced disease in the event of Adverse Event of Special Interest (AESI), DSMB members may analyze the relationship between occurred AESI and IP and confirm whether AESI is Enhanced disease or not.

In the event of AEs that meet the ‘criteria for study suspension’ during the course of Part A and Part B, the DSMB will meet to assess the safety data and decide on study continuation. However, the placebo group is not subject to review by the DSMB.

8. Adverse events

8.1 Definition of adverse events

(1) AEs

An AE is any unfavorable and unintended sign (including laboratory test abnormalities), symptom, or disease occurring to a subject treated with the IP, which does not necessarily have to have a causal relationship to the relevant IP.

(2) ADRs

An ADR is any unfavorable and unintended event occurring at any dose of the IP for which causal relationship to the IP cannot be ruled out.

(3) Unexpected ADRs (UADRs)

A UADR is any ADR that differs from available drug information in the profile or the extent of risk of the event in light of the investigator’s brochure or the package insert.

(4) SAEs/SADRs

An SAE/SADR is any AE/ADR occurring at any dose of the IP that meets any of the followings.

① resulting in death or life-threatening
② requiring hospitalization or prolonged hospitalization
③ leading to persistent or significant incapacity or incapacity
④ leading to congenital anomaly or birth defect
⑤ other important medical event except for the above ① to ④, such as development of blood dyscrasias or drug dependence/abuse.

In this study, hospitalization for any of the following reasons will not constitute an SAE:

- Hospitalization that was planned prior to study
- Hospitalization for medical checkup or recuperation or for cosmetic reasons
- Emergency room visit for no longer than 24 hours. However, an emergency room visit for no longer than 24 hours can be considered an SAE at the discretion of the investigator.

For an event that is not one of the situations listed above but may have a significant medical impact on the health and well-being of the patient, a decision will be made as to the status of seriousness based on the medical judgment of the attending physician and relevant specialists,

and appropriate actions will be taken accordingly.

8.2 Collection and documentation of adverse events

- During the study, AEs will be collected from after the 1st dose (Visit 2) to Day 28 after the 2nd dose of the IP (Visit 9). All medical events occurring before the 1st dose of the IP will be documented as medical history.
- SAEs and AESIs will be collected from after the 1st dose (Visit 2) of the IP to the end of follow-up (Visit 11).
- Once AEs grade ≥3 are occurred during the course of 1st dose (Visit 2) to the end of follow-up (Visit 11), subjects should inform study institution or investigator through telephone call with reference to severity assessment of adverse events in subject diary, and investigator should primarily check symptoms via telephone call. Subjects who requires treatment of AEs by investigator will perform unscheduled visit for treatment with medication.
- Subjects who have the symptoms of COVID-19 infection such as fever or respiratory symptoms should inform investigator about their symptoms as followed by COVID-19 guidelines of Central Disease Control Headquarters·Central Disaster Management Headquarters, and will be instructed to visit study institution to be treated, observed and assessed. Investigator or study coordinator should follow up the AEs in subjects who are not available to visit study institution via telephone call.
- An AE report should include the following information: term of AE, duration (date of onset and date of resolution), severity, causal relationship to the IP, action taken for the IP, outcome, corrective treatment for the AE, and status of immediate AE/SAE.
- The investigator should document AEs as comprehensive diagnostic terms or symptoms using standard medical terminology rather than recording individual symptoms or signs.
- AEs occurring during the study should be followed up until the event is resolved or stabilized or the subject is lost to follow-up. Follow-up of AEs of which date of resolution is unknown as of Day 28 after the 2nd dose (Visit 6) will be determined at the discretion of the investigator.
- AEs occurring after the end of follow-up (Visit 11) will spontaneously be reported by subjects; if a subject reports to the investigator an AE that is considered to be an SADR, the investigator will report to the sponsor.

8.3 Assessment of adverse events

8.3.1 Severity assessment

Severity of solicited or unsolicited AEs in subjects enrolled in this study will be classified as follows based on the assessment of severity of local and systemic AEs in healthy adults and adolescents in 「MFDS, Guidelines for the assessment of severity of adverse events in vaccine trials」[2].

8.3.1.1 Solicited local adverse events

Grade / Item	Mild (GRADE 1)	Moderate (GRADE 2)	Severe (GRADE 3)	Potentially life-threatening (GRADE 4)
**Pain**	No interference with activities	Repeated use of non-opioid analgesics for > 24 hours or some interference with daily activities	Use of opioid analgesics or severe interference with daily activities	Emergency room visit or hospitalization
**Tenderness**	Mild discomfort to touch	Discomfort to exercise	Considerable discomfort at rest	Emergency room visit or hospitalization
**Erythema/redness ***	2.5~5cm	5.1~<10cm	≥10cm	Necrosis or exfoliative dermatitis
**Induration/swelling ***	2.5~5cm and no interference with activities	5.1~<10cm or some interference with activities	≥10cm or severe interference with daily activities	Necrosis
**Itchiness**	Slight itchiness at the injection site	Considerable itchiness at the injected limb	Itchiness at the injected limb and other areas of the body	Itchiness all over the body

* Measured local reactions will be graded on the basis of the single longest diameter.

8.3.1.2 Solicited systemic adverse events

Grade / Item	Mild (GRADE 1)	Moderate (GRADE 2)	Severe (GRADE 3)	Potentially life-threatening (GRADE 4)
**Fever ※**	38.0~38.4℃	38.5~38.9℃	39.0~40.0℃	>40.0℃

Grade / Item	Mild (GRADE 1)	Moderate (GRADE 2)	Severe (GRADE 3)	Potentially life-threatening (GRADE 4)
**Fatigue/malaise** ※※	Easily tolerable and no interference with activities	Some interference with daily activities, making it difficult to perform several activities	Considerable; severe interference with daily activities resulting in loss of abilities to perform several activities	Emergency room visit or hospitalization
**Chills/rigors**	Easily tolerable and no interference with activities	Some interference with daily activities, making it difficult to perform several activities	Severe interference with daily activities resulting in loss of abilities to perform several activities	Emergency room visit or hospitalization
**Headache**	Easily tolerable and no interference with activities	- Repeated use of non-opioid analgesics for > 24 hours<br>-Interference with brain function<br>- Some interference with daily activities	Use of opioid analgesics or severe interference with daily activities	Persistence leading to emergency room visit or hospitalization
**Myalgia**	Easily tolerable and no interference with activities	Interference with muscle function or some interference with daily activities	Severe interference with daily activities resulting in loss of abilities to perform several activities	Emergency room visit or hospitalization
**Joint pain**	Mild pain that does not interfere with function (with inflammation, redness, or joint swelling)	Moderate pain interfering with function but no interference with daily activities (with inflammation, redness, or joint swelling)	Severe pain interfering with daily activities (with inflammation, redness, or joint swelling)	Emergency room visit or hospitalization
**Diarrhea**	2 to 3 loose stools or < 400 g during a 24-hour period	4~5 loose stools or 400~800 g during a 24-hour period	≥6 loose stools or ≥800 g during a 24-hour period or requiring fluid therapy on an outpatient setting	Emergency room visit or hospitalization
**Vomiting**	No interference with activities or 1~2 times/24	Some interference with activities or >2 times/24	Severe interference with daily activities or	Emergency room visit or hospitalization for

Grade / Item	Mild (GRADE 1)	Moderate (GRADE 2)	Severe (GRADE 3)	Potentially life-threatening (GRADE 4)
	hours	hours	requiring fluid therapy on an outpatient setting	hypotensive shock
**Abdominal pain**	Weak	Moderate (no treatment required)	Moderate (treatment required)	Severe (hospitalization)
**Mucocutaneous reaction/rash**	Erythema (itchiness) or limited macular rash	Extensive, maculopapular rash, dry desquamation	Vesication or wet desquamation or ulceration	-
**Cough**	Temporary (no treatment required)	Persistent cough (treatment required)	Paroxysmal cough (not controlled by treatment)	-
**Acute bronchospasm**	- Temporary (no treatment required)<br>-70-80% of FEV_1 at peak expiratory flow	- Treatment required; recovery to normal with bronchodilator<br>- 50-69% of FEV_1 at peak expiratory flow	-No recovery to normal with bronchodilator<br>- 25-49% of FEV_1 at peak expiratory flow; or chest depression	-
**Respiratory distress**	Respiratory distress during exercise	Respiratory distress during normal activities	Respiratory distress at rest	-

※ Fever is defined as an increase in body temperature to ≥38℃ based on at least one measurement of the core temperature. Once fever is detected, body temperature should be measured at least twice a day (morning and evening) or at clinically appropriate time points until 2 consecutive measurements of <38℃ are obtained.

※※ Interference of individual activities refers to deterioration of the individual's function to perform his/her daily activities at work or school or his/her social or personal activities.

8.3.1.3 Unsolicited adverse events

Severity of unsolicited AEs including immediate AEs (anaphylaxis related) observed within 30 minutes after IP dosing will be classified as follows based on the assessment of severity of local and systemic AEs in healthy adults and adolescents in 「MFDS, Guidelines for the assessment of severity of adverse events in vaccine trials」[2).]

Grade / Item	Mild (GRADE 1)	Moderate (GRADE 2)	Severe (GRADE 3)	Potentially life-threatening (GRADE 4)
**AE term**	- Temporary or mild discomfort (<48 hours); no medical intervention/treatment required<br>- No impact on daily activities	- Mild to moderate restriction on daily activities (able to perform ≥50% of usual activities)<br>- May require some help (no or minimal medical intervention/treatment required)	- Marked restriction on daily activities (able to perform <50% of usual activities)<br>- Usually requires some help (medical intervention/treatment required, hospitalization possible)	- Life-threatening or serious restriction on activities<br>- Requires considerable help (medical intervention/treatment required, hospitalization highly likely)<br>- Serious or life-threatening AE ¥

¥ A clinical AE considered by the investigator to be serious or life-threatening must be regarded as a grade 4 AE. Serious or life-threatening AEs include, but are not limited to the followings: convulsion, coma, tetany (symptoms of hypocalcemia such as spasticity or contraction), diabetic ketoacidosis, disseminated intravascular coagulation, petechiae, paralysis, acute psychosis, severe depression.

It should not be confused that AEs or SAEs can both be assessed as severe based on the above definition whereas severe AEs are not necessarily also serious.

8.3.2 Adverse events of special interest

AESIs are clinically important, unexpected medical reactions that are known to occur with the IP or are considered to be the potential risks of the IP. With 「MFDS, Considerations in COVID-19 vaccines development (2020.09.28)」[1] taken into account, AESIs to be collected in this study are as follows.

System organ class	AESIs
**Immune**	Enhanced disease*
	Multisystem inflammatory syndrome in children
**Respiratory system**	Acute respiratory distress syndrome

**Cardiovascular system**	Acute cardiac injury including
	• Microangiopathy
	• Heart failure and cardiogenic shock
	• Stress cardiomyopathy
	• Coronary artery disease
	• Arrhythmia
	• Myocarditis, pericarditis
**Blood**	Coagulation disorder
	• Deep vein thrombosis
	• Pulmonary embolus
	• Cerebrovascular stroke
	• Limb ischemia
	• Hemorrhagic disease
**Nervous system**	Guillain-Barré Syndrome
	Anosmia, ageusia
	Meningoencephalitis
**Skin**	Chilblain-like lesions
	Single organ cutaneous vasculitis
	Erythema multiforme
**Other**	Acute kidney injury
	Liver injury

* Enhanced disease refers to a phenomenon that those vaccinated have more serious AEs of viral infection than those unvaccinated. This was identified in the course of development of vaccines such as inactivated respiratory syncytial virus vaccine and dengue vaccine. Enhanced disease is thought to be associated with humoral immune response (antibody-dependent enhancement [ADE]) and cellular immune response (enhanced respiratory disease [ERD]). ADE refers to a phenomenon that non-neutralizing antibodies generated by the vaccine antigens facilitate viral infection via Fcγ receptors (FcγRs) on the cells. ERD refers to excessive invasion of immune cells (e.g., eosinophil) into the lungs of vaccinated individuals due to excessive cellular responses by Th2 (Type 2 Help T).

In the event of AESIs in subjects treated with the IP, additional tests may be conducted as necessary for assessment at the discretion of the investigator, and if necessary, the DSMB may be asked to additionally review the case for status of ADR and enhanced disease.

8.3.2 Assessment of causal relationship

In the event of an AE, association with the IP will be assessed by the investigator according to 2-

level criteria: ‘related’ or ‘not-related’.

(1) Not-related

An AE that has a temporal relationship to IP dosing which makes a causal relationship improbable, or that can be reasonably explained by other factors rather than IP dosing.

(2) Related

All AEs that are not applicable to not-related.

For ‘related’ AEs, a sub-level of causal relationship can additionally be assessed as necessary.

① **Definitely related**

- Evidence of IP dosing and an AE occurring in reasonable time sequence to IP dosing.
- The event can be most reasonably explained by IP dosing above all possible causes.
- Disappearance of the event upon IP withdrawal and reappearance of the event upon rechallenge.
- The profile of the event is consistent with known information on the IP or drugs of the same class.

② **Probably related**

- Evidence of IP dosing and an AE occurring in reasonable time sequence to IP dosing.
- The event can be more reasonably explained by IP dosing than any other causes.
- Disappearance of the event upon IP withdrawal.

③ **Possibly related**

- Evidence of IP dosing and an AE occurring in reasonable time sequence to IP dosing.
- The event is attributable to IP dosing as much as to other possible causes.
- Disappearance of the event upon IP withdrawal.

④ **Probably not related**

- Evidence of IP dosing.
- There are other more plausible causes of the AE.
- Disappearance of the event or an ambiguous outcome upon IP withdrawal.
- No reappearance of the event or an ambiguous outcome upon rechallenge.

⑤ **Unassessable/unclassifiable**

- The event cannot be judged because information is insufficient or contradictory and cannot be supplemented or verified.

8.4 Reporting serious adverse events

All SAEs occurring from after the 1st dose of the IP (Visit 2) to the end of study (Visit 11) should be reported, regardless of relationship to the IP, to the sponsor via telephone/fax/email within 24 days from when the event is known to the investigator and to the IRB at the institution within the period defined by the IRB.

AEs occurring after the end of study (Visit 11) will spontaneously be reported by subjects; the investigator will report to the sponsor only serious events that are related to the IP.

To protect subject privacy, the investigator should report SAEs to the sponsor using subject identification codes instead of personal information such as the subject's name, resident registration number, and address. If copies of some medical records are required, personal information except for the identification code will be blocked.

If the investigator obtains additional information about the reported SAE, he/she should submit a follow-up report. The investigator should follow up the SAE on a regular basis until the event is resolved or stabilized or the subject is lost to follow-up and should report to the sponsor and the IRB.

8.5 Reporting suspected unexpected serious adverse reactions and actions to be taken

If suspected unexpected serious adverse reactions (SUSAR) occur during the study period, the sponsor will report to the investigator, Minister of Food and Drug Safety, and if necessary, the IRB. For details, see Attachment 4. Good Clinical Practice and Guidance for Civil Petitioners 'Considerations for safety assessment and reporting by clinical trial sponsors', MFDS Rules on the Safety of Medicinal Products, etc.

③ UADRs that result in death or are life-threatening should be reported via telephone or fax

or in writing as soon as possible but no later than 7 days from when the event was reported or known to the sponsor. If all information according to ADR reporting is not reported, an additional detailed report should be submitted within 15 days from the relevant ADR was initially reported or known.

④ All other SUSARs will be reported within 15 days from when the event was reported or known to the sponsor.

In the event of “SUSARs” during the study, the principal investigator and the sub-investigator should make every effort to ensure the safety of subjects and take appropriate actions to minimize AEs in a timely manner. If there is additional information on the reported ADR, the sponsor should report until the relevant ADR is resolved (i.e., the ADR disappears or the subject is lost to follow-up).

If the study is conducted at multiple institutions, the sponsor should promptly notify to the institutions through the principal investigator.

8.6 Pregnancy

Pregnancy in a female subject during the study period is not considered an AE, and hospitalization for elective abortion without complications (therapeutic abortion is not applicable) or for normal delivery of a healthy infant is also not considered an AE.

However, if a female subject becomes pregnant during the study period (Visit 1 to Visit 11), the subject should be withdrawn from the study, and the investigator should complete a pregnancy report and submit it to the sponsor within 24 hours from when the pregnancy is known. Even if the subject stops participating in the study or withdraws from the study, the investigator should follow up and report the course of the pregnant woman and the fetus until childbirth.

Serious complications to the mother, spontaneous abortion, ectopic pregnancy, stillbirth, and death or congenital malformation of the infant will be considered SAEs and should be reported by the investigator according to procedures specified in Section 8.4.

9. Ethical considerations and administrative procedures

9.1 Protocol compliance

The investigator should conduct the study in compliance with the protocol. Except when necessary to remove immediate risks to subjects, the study should not be deviated from the protocol. Any protocol deviations should be documented along with reasons for the deviations. Even if the investigator determines that a protocol deviation can improve the study conduct, it should not be implemented before the amendment is agreed upon by the sponsor and approved by the IRB (including the Minister of Food and Drug Safety, if necessary).

9.2 Protocol approval and amendment

To obtain approval for the study or to amend the approved study, the protocol of the applicable development phase or the protocol amendment should be approved by the IRB, and if necessary, by the Minister of Food and Drug Safety. Subjects are not allowed to participate in the study prior to approval. The investigator will not implement any amendment of the study without written approval from the IRB, with an exception of a situation where it is necessary to remove clear, immediate risks to subjects.

9.3 Consenting procedures

The subject information sheet and ICF may be used after they are approved by the IRB. The investigator should obtain informed consent from the subjects in compliance with GCP and ethical principles laid down in the Declaration of Helsinki. Prior to any of the study related procedures, the investigator must obtain written informed consent from the subjects after providing the subjects (or their representatives) with full explanations about the study. The investigator should retain the original signed ICF in the investigator's file and provide the subject (or his/her representative) a copy of the signed ICF and the subject information sheet used for explanations.

If it is impossible for the subject to provide consent due to lack of ability to understand and express himself/herself, consent should be obtained from the subject's representative. According to Article 34-2, Paragraph 3, Subparagraph 3 of Pharmaceuticals Affairs Act, the representative should be a legally acceptable representative, or in the absence of a legally acceptable representative, the

representative should be in the order of spouse, lineal ascendant, and lineal descendant; if there are more than one lineal ascendants or lineal descendants, a decision will be made based on agreement; if agreement is not reached, the older will be the representative.

If both the subject and the subject's representative are illiterate, a witness should be present throughout the consenting procedures. After the subject and his/her representative give oral consent to study participation, and if possible, sign the ICF, the witness will verify, by signing the ICF, that the information on the ICF was accurately explained and understood.

If amendments are to be made to the subject information sheet and ICF, all amendments should be re-approved by the IRB before the relevant form is implemented, and reconsent should also be obtained from the subjects (or their representatives) who are already participating in the study. In this case, the investigator should record those who are notified as well as date and content of the notification in the source documents.

9.4 Measures to protect the safety of subjects

The investigator should conduct the study in compliance with the Declaration of Helsinki and by considering the rights and well-being of study subjects. Investigators participating in this study should be familiar with GCP as well as the protocol. The investigator will allocate a sufficient amount of time to interviewing and testing each subject to thoroughly assess their eligibility and status of AEs.

The principal investigator will report AEs, study progress, situation, and outcomes to the sponsor on a regular basis, and the sponsor will manage study progress on a regular basis.

9.5 Measures to protect vulnerable subejcts for study enrollment

'Vulnerable Subjects' refer to subjects whose voluntary decision to study participation may be affected by expected benefits from study participation or concerns about penalties from their superiors according to the hierarchy of the organization for refusal to study participation (students of college of medicine, Korean medicine, pharmacy, dentistry, and nursing; employees of medical research centers; employees of pharmaceutical companies; soldiers, etc.), patients with terminal

illness, individuals institutionalized in group facilities according to Article 27 ('Rules on the Safety of Medicinal Products'), the unemployed, the poor, patients in emergency situations, ethnic minorities, vagrants, the homeless, refugees, minors, and subjects who cannot voluntarily provide informed consent.

To enrol vulnerable subjects, it should be checked whether the subject has sufficient abilities to provide informed consent and if additional consenting procedures are needed. Of particular note, the importance of voluntary participation should be stressed, and the subject should be ensured to voluntarily decide whether to participate in the study or not. Study participation must not be coerced in any form.

If an employee of the study institution, the investigator, or the sponsor or a student of the investigator who qualifies the definition of vulnerable subjects and is eligible for study enrolment wishes to participate in the study, the investigator must abide by the followings:

1) The investigator must not have direct interactions with the employee for the purpose of subject recruitment.
2) If possible, study recruitment or consenting procedures should be carried out in the absence of the employee's superior.
3) The employee should be informed that he/she can decide not to participate in the study and that this decision will not affect his/her employment or job evaluation.
4) Procedures should be in place that can ensure that the employee will not be unfairly affected or forced and that his/her privacy will be respected.
5) Action should be taken to ensure that the employee's refusal to study participation is not disclosed to his/her superior.
6) If possible, study activities should be performed in the absence of other employees (superiors, colleagues, etc.).
7) If the sponsor's employee is to be recruited, measures should be taken to protect the employee's identity by removing all personal information from all data or summaries to be provided to the sponsor.

9.6 Criteria for post-study medical examination and treatment for subjects

Subjects who completed the study may be treated at any time as instructed by the investigator for

possible unexpected AEs. If a subject has an injury from study participation which is subject to compensation from the sponsor according to indemnification provisions for subjects, the treatment expenses may be compensated even after the end of study depending on the persistence of the injury.

9.7 Indemnification provisions for subjects

In terms of damages from AEs caused by the IP and/or from the process of corrective treatment of AEs, subjects will be compensated by the sponsor for damages that are directly caused by the IP according to indemnification provisions for subject.

9.8 Study related documents and record keeping

9.8.1 Case report forms and source documents

Study data will be collected according to FDA 21 CFR part 11 and/or MFDS Guidelines for handling of electronic data in clinical trials by using electronic-CRFs (e-CRFs). Investigators and/or designees who are to use e-CRFs should receive relevant trainings and be assigned a unique user account for the electronic data capture system. If the investigator and/or the designee is to no longer participate in the study, the investigator will inform the sponsor (including the CRO) to have the relevant investigator’s access inactivated. The investigator is responsible for the accuracy, completeness, clearness, and timeliness of information in e-CRFs.

An e-CRF will be completed for each subject; the investigator and/or the designee should enter collected information in the e-CRF as soon as possible. Data in e-CRFs should be consistent with those in source documents. The investigator will check all e-CRFs completed at the relevant institution and electronically sign them.

The CRA will compare CRFs with source documents; if any discrepancies are noted, the CRA will inform the investigator and ask him/her to make corrections as appropriate. Only the investigator and/or the designee may enter and correct CRFs and source documents.

9.8.2 Access to source documents

The sponsor, the CRA, and the auditor involved in this study may access the records of subjects for the purpose of monitoring and auditing of the study as well as management of study progress. The investigator should understand that, upon completion of the study contract, the sponsor's or the CRO's CRA and auditor may access and review the charts and CRFs of subjects in order to verify the relevant documents. These data should be kept in confidence, and facilities and SOPs for confidential storage should be in place. The investigator should ensure that support will be available as needed for the CRO and the sponsor.

The investigator should allow authorized representatives of the IRB, regulatory authorities, and the sponsor to have direct access to the original medical records of subjects for verification of study related procedures and data.

9.8.3 Archiving study data

The investigator should safely store data and records related to study conduct in a secure area in which the data will be stored for 3 years from the date of approval of the IP or the date of discontinuation of the development program. Once the CSR is prepared, the study documents should be transferred to a person in charge of storage; if the investigator is to destroy or relocate the study documents, this should be notified to the sponsor in advance. If it is instructed by the Minister of Food and Drug Safety or deemed necessary by the sponsor, the duration of storage may be extended. The sponsor should notify in writing the necessity and duration of data storage to the investigator and the head of the institution. If it is decided that the data no longer needs to be stored, this should be notified in writing by the sponsor to the principal investigator and the head of the institution.

9.8.4 Audits and inspections

To ensure and GCP and all other relevant regulations are complied with, the sponsor or a designee may perform quality assurance and auditing, and the MFDS may conduct inspections for the study. Upon receipt of an appropriate notice, the investigator should cooperate with audits or inspections, allow the auditor or the inspector to have direct access to all study related documents, and agree to allocate time to discussing all findings and other related matters.

9.9 Confidentiality of study documents and subject records

All study results and documents are considered to be confidential. The investigator, the CRO, and the sponsor's personnel must not disclose study related information without the sponsor's signed approval.

Records in which subjects are identifiable with their names and medical record numbers will be kept in confidence by storing them in a separate locked space with limited access under the responsibility of the principal investigator. In all study related documents including CRFs, subjects will be recorded and identified not by their names but by subject identification codes. Subject identities will remain confidential in the publication of study results.

9.10 Monitoring of institutions

To protect the rights and well-being of study subjects, to examine the accuracy, completeness, and verifiability of data by checking the reported study related data against the relevant source documents, and to ensure that the study is conducted in compliance with the approved protocol, Article 30 and Attachment 4. Good Clinical Practice, Rules on the Safety of Medicinal Products, etc., monitoring will be conducted on a regular basis.

A CRA designated by the sponsor will monitor the study on a regular basis by on-site and telephone visits. The CRA will assess study progress and check whether the investigator is conducting the study in compliance with the protocol and applicable regulations. During the on-site visits, the CRA will check the storage of original subject records, CRFs, drug management records, and study related data; if any discrepancies or other issues are identified in the study records, the CRA will discuss them with the investigator.

9.11 Early termination or suspension of the study

9.11.1 Sponsor

If the institution, the investigator, or the sponsor's designee does not comply with essential obligations including GCP, the protocol, the contract, and applicable laws and regulations, the sponsor will immediately take actions for rectification. If any of the followings occur, the sponsor

may decide to prematurely discontinue the study as whole or at a specific institution.

1) Failure to enroll the target number of subjects as whole or at a specific institution.
2) Efficacy and safety information that might have a significant impact on the continuation of the study becomes known.
3) The extent of violation of GCP, the protocol, the contract, and applicable laws and regulations by the institution or the investigator raises an issue in the continuation of the study.
4) Other administrative reasons that might have a significant impact on the continuation of the study
5) The DSMB recommends that the study should be prematurely terminated or suspended.

If the study is prematurely terminated or suspended, the sponsor should report in writing the fact and its reasons to the principal investigator and the Minister of Food and Drug Safety in a prompt manner; for multicenter studies, the sponsor should notify in writing the fact and its reasons to the principal investigators at other institutions as well.

9.11.2 Investigator

If the study is prematurely terminated or suspended by the sponsor, the principal investigator should immediately inform the IRB and submit to the IRB a detailed explanatory statement for the early termination and suspension and should also immediately inform the subjects and ensure that appropriate actions are taken including follow-ups.

If the principal investigator is to prematurely terminate or suspend the study, he/she should inform the sponsor of the fact in advance, immediately inform the IRB of the decision and its reasons, and submit to the IRB a detailed explanatory statement for the early termination and suspension.

9.12 Clinical study report and publication

Upon completion of all analyses of data from the institutions, the sponsor will prepare a CSR and notify the study results to the investigator.

The sponsor owns all data and results derived from this study and has the right to publish the study results at any time. The investigator should not publish, present, or disclose any information

related to the study without prior written approval from the sponsor and should ensure that this is also complied with by the sub-investigator. To ensure that only accurate and verified data are used, it is essential that the investigator should provide the sponsor with any drafts for publications or any manuscripts for presentations prior to publications and presentations and discuss them with the sponsor and should put presentations on hold until written approval is obtained from the sponsor.

For multicenter trials, the investigator agrees that he/she will not publish the results from his/her institution or from some institutions before the results from all institutions are published. It is exceptional however if such publication is officially acknowledged by the sponsor and the principal investigators of all institutions.

10. References

1) Ministry of Food and Drug Safety, Considerations in COVID-19 vaccines development (2020.09.28)
2) Ministry of Food and Drug Safety, Guidelines for the assessment of severity of adverse events in vaccine trials [Guidance for civil petitioners](2011.12)
3) Ministry of Food and Drug Safety, Guidelines for the establishment and operation of independent data monitoring committees (2009)
4) Korean Society for Laboratory Medicine, Q&A for COVID-19 testing, version 4(20200630)
5) Diagnosis and Management Team, Central Disaster and Safety Countermeasures Headquarters, Korea Centers for Disease Control & Prevention, Guidelines for collecting samples to test new corona virus (2020.01.23)
6) EuBiologics Co., Ltd., Investigator's Brochure for EuCorVac-19 V1.0, 11DEC2020
7) C. Keech, G. Albert, I. Cho, A. Robertson, P. Reed, S. Neal, J. S. Plested, M. Zhu, S. Cloney-Clark, H. Zhou, G. Smith, N. Patel, M. B. Frieman, R. E. Haupt, J. Logue, M. McGrath, S. Weston, P. A. Piedra, C. Desai, K. Callahan, M. Lewis, P. Price-Abbott, N. Formica, V. Shinde, L. Fries, J. D. Lickliter, P. Griffin, B. Wilkinson, and G. M. Glenn (2020). Phase 1-2 Trial of a SARS-CoV-2 Recombinant spike protein nanoparticle vaccine. The New England Journal of Medicine, September 3, 2020. DOI: 10.1056/NEJMoa2026920
8) Pedro M Folegatti, Katie J Ewer, Parvinder K Aley, Brian Angus, Stephan Becker, Sandra Belij-Rammerstorfer, Duncan Bellamy, Sagida Bibi, Mustapha Bittaye, Elizabeth A Clutterbuck, Christina Dold, Saul N Faust, Adam Finn, Amy L Flaxman, Bassam Hallis, Paul Heath, Daniel Jenkin, Rajeka Lazarus, Rebecca Makinson, Angela M Minassian, Katrina M Pollock, Maheshi Ramasamy, Hannah Robinson, Matthew Snape, Richard Tarrant, Merryn Voysey, Catherine Green, Alexander D Douglas, Adrian V S Hill, Teresa Lambe, Sarah C Gilbert, and Andrew J Pollard (2020). Safety and immunogenicity of the ChAdOx1 nCoV-19 vaccine against SARS-CoV-2: a preliminary report of a phase 1/2, single-blind, randomized controlled trial. The Lancet. Published online July 20, 2020. https://doi.org/10.1016/S0140-6736(20)31604-4
9) Feng-Cai Zhu, Yu-Hua Li, Xu-Hua Guan, Li-Hua Hou, Wen-Juan Wang, Jing-Xin Li, Shi-Po Wu, Bu-Sen Wang, Lei Wang, Si-Yue Jia, Hu-Dachuan Jiang, Ling Wang, Tao Jiang, Yi Hu, Jin-Bo Gou, Sha-Bei Xu, Jun-Jie Xu, Xue-Wen Wang, Wei Wang, Wei Chen (2020). Safety, tolerability, and immunogenicity of a recombinant adenovirus type-5 vectored COVID-19 vaccine: a dose-escalation, open-label, non-randomsed, first-in-human trial. The Lancet. Published online May 22, 2020. https://doi.org/10.1016/S0140-6736(20)31208-3.
10) L.A. Jackson, E.J. Anderson, N.G. Rouphael, P.C. Roberts, M. Makhene, R.N. Coler, M.P.

McCullough, J.D. Chappell, M.R. Denison, L.J. Stevens, A.J. Pruijssers, A. McDermott, B. Flach, N.A. Doria-Rose, K.S. Corbett, K.M. Morabito, S. O'Dell, S.D. Schmidt, P.A. Swanson II, M. Padilla, J. R. Mascola, K.M. Neuzil, H. Bennett, W. Sun, E. Peters, M. Makowski, J. Albert, K. Cross, W. Buchanan, R. Pikaart-Tautges, J.E. Ledgerwood, B.S. Graham, and J.H. Beigel (2020). An mRNA Vaccine against SARS-CoV-2 – Preliminary report. The New England Journal of Medicine. Published on July 14, 2020. DOI: 10.1056/NEJMoa2022483

11) Mark J. Mulligan, Kirsten E. Lyke, Nicholas Kitchin, Judith Absalon, Alejandra Gurtman, Stephen Lockhart, Kathleen Neuzil, Vanessa Raabe, Ruth Bailey, Kena A. Swanson, Ping Li, Kenneth Koury, Warren Kalina, David Cooper, Camila Fontes-Garfias, Pei-Yong Shi, Ozlem Tureci, Kristin R. Tompkins, Edward E. Walsh, Robert Frenck, Ann R. Falsey, Philip R. Dormitzer, Wiliam C. Gruber, Ugur Sahin and Kathrin U. Jansen (2020). Phase 1/2 study of COVID-19 RNA vaccine BNT162b1 in adults. Nature. https://doi.org/10.1038/s41586-020-2639-4.

12) Ministry of Food and Drug Safety, Guidelines for the establishment and operation of independent data monitoring committees (2009)

11. List of attachments

Attachment 1. Subject information sheet and informed consent form - Part A (Phase 1)

Attachment 2. Subject information sheet and informed consent form - Part B (Phase 2)

Attachment 3. Study institutions and the sponsor's organizations

Attachment 4. Indemnification provisions for subjects

Attachment 5. Solicited side effects of investigational products and precautions for use

Attachment 6. Human-derived material ICF – Part A(Phase I)

Attachment 7. Human-derived material ICF – Part B(Phase II)

CONFIDENTIAL
